# Lipoyl deglutarylation by ABHD11 regulates mitochondrial and T cell metabolism

**DOI:** 10.1038/s41589-025-01965-6

**Published:** 2025-07-15

**Authors:** Guinevere L. Grice, Eleanor Minogue, Hudson W. Coates, Mekdes Debela, Richard J. Stopforth, Niek Wit, Zongyu Li, Joseph P. Crowley, Arthur Kaser, Nicole Kaneider-Kaser, P. Robin Antrobus, Marcia C. Haigis, Randall S. Johnson, James A. Nathan

**Affiliations:** 1 Cambridge Institute of Therapeutic Immunology & Infectious Disease (CITIID), Jeffrey Cheah Biomedical Centre, Department of Medicine, University of Cambridge, Cambridge, UK; 2 Department of Physiology, Development and Neuroscience, University of Cambridge, Cambridge, UK; 3 Department of Cell Biology, Blavatnik Institute, Harvard Medical School, Boston, MA USA; 4 Cambridge Institute for Medical Research, University of Cambridge, Cambridge, UK; 5 Department of Cell and Molecular Biology, Karolinska Institute, Stockholm, Sweden

**Keywords:** Post-translational modifications, Enzyme mechanisms, Immunology, Metabolic pathways

## Abstract

Glutarate is an intermediate of amino acid catabolism and an important metabolite for reprogramming T cell immunity. Glutarate exerts its effects either by directly inhibiting metabolite-dependent enzymes or through conjugation to substrates. Intriguingly, glutarylation can occur on protein and nonprotein substrates, but our understanding of these distinct glutaryl modifications is in its infancy. Here we uncover ABHD11 as a noncanonical deglutarylating enzyme critical for maintaining the tricarboxylic acid (TCA) cycle. Mechanistically, we find ABHD11 removes glutaryl adducts from lipoate—an essential fatty acid modification required for the TCA cycle. Loss of ABHD11 results in the accumulation of glutaryl–lipoyl adducts that drive an adaptive program, involving 2-oxoglutarate accumulation, that rewires mitochondrial metabolism. Functionally, this role of ABHD11 influences the metabolic programming of human CD8^+^ T cells. Therefore, our findings reveal lipoyl glutarylation as a reversible modification that regulates the TCA cycle.

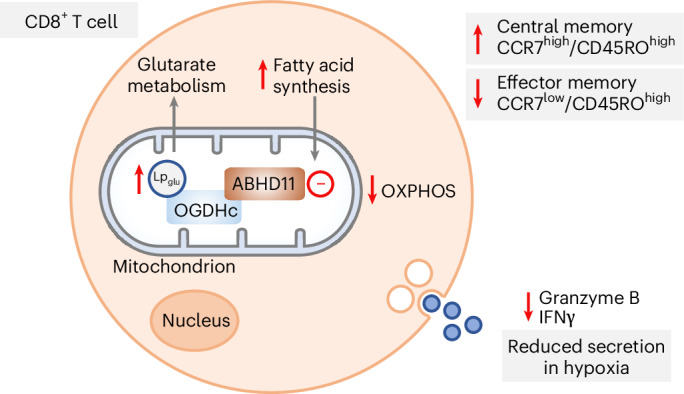

## Abstract (web summary)

Glutarate rewires cell metabolism through conjugation to proteins or fatty acids, but how glutarylation is regulated is unclear. ABHD11 was identified as a lipoyl-deglutarylase to maintain the TCA cycle and mitochondrial metabolism.

## Main

Post-translational modifications by small-molecule metabolites are increasingly recognized as determinants of cellular metabolic reprogramming and cell fate decisions. Examples such as acetylation and succination highlight the established importance of metabolite modifications, with diverse roles ranging from chromatin regulation to immune effector responses^1–4^. Glutarate, a product of lysine or tryptophan catabolism, is a particularly interesting metabolite, as it can be conjugated to proteins or nonprotein substrates (for example, fatty acids), with diverse outcomes for mitochondrial function^5–7^, metabolism^8^ and T cell immunity^9^.

Glutarate is formed by the conversion of 2-oxoadipic acid to glutaryl-coenzyme A (glutaryl-CoA) within the mitochondria. Glutaryl-CoA can then generate free glutarate or be metabolized to acetyl-CoA. Human germline mutations in glutarate metabolism highlight its fundamental biological role, with loss of function mutations in glutaryl-CoA dehydrogenase (GCDH) resulting in glutarate accumulation and glutaric aciduria type 1 (GA1)—a rare autosomal disorder characterized by dystonia, developmental delay and often death in early childhood^10^.

Glutarylation on lysine residues (K_glu_) was first observed on mitochondrial proteins, where glutarylation of carbamoyl phosphate synthetase 1 reduced enzyme activity and altered ammonia clearance^5^. Subsequently, glutarylation has been observed on other proteins, including histones, where K_glu_ can alter chromatin structure and dynamics^11^. The potential regulatory nature of K_glu_ was supported by observations that dietary tryptophan supplementation alters K_glu_, and that SIRT5 can act as a protein deglutarylating enzyme^5,12^.

Recently, we discovered that glutarylation is a dynamic modification in CD8^+^ T cells, with glutarylation patterns changing in response to T cell receptor (TCR) activation^9^. Glutarate accumulation resulted in protein glutarylation as expected, but we also identified additional functions of glutarate. First, it acted as a competitive inhibitor of 2-oxoglutarate-dependent dioxygenase (2-OGDD) function, and secondly, glutarate accumulation impaired activity of the pyruvate dehydrogenase complex (PDHc). This latter finding is of particular interest, as rather than simply modifying the PDHc, glutarate formed a fatty acid conjugate that binds to the lipoylated catalytic arm of PDHc.

Lipoylation is an essential mitochondrial modification arising from conjugation of lipoic acid (lipoate), a redox-sensitive fatty acid, to lysine residues (K_Lp_)^13,14^. Five mitochondrial enzymes are known to require lipoylation for catalysis—the PDHc, 2-oxoglutarate dehydrogenase complex (OGDHc), 2-oxoadipate dehydrogenase complex (OADHc), branched-chain α-ketoacid dehydrogenase complex (BCKDHc) and the glycine cleavage system complex (GCVc). Lipoylation drives catalysis of these ketoacid dehydrogenase complexes via the cyclical oxidation and reduction of its thiol ring, described as the ‘swinging arm’ of the dehydrogenases. However, the reactive thiols of lipoate also render it susceptible to attack, resulting in lipoyl conjugates or adducts. We observed that glutarate can react with the thiols of lipoate, thereby blocking the enzymatic activity of PDHc^9^. Additionally, we and others have observed that other lipoyl adducts can be formed when lipid peroxidation products accumulate or following nitrosylation during macrophage activation^15–18^. However, whether these lipoate conjugates can be removed to repair normal lipoate redox cycling is not known.

Here we show that glutaryl–lipoyl adducts (Lp_glu_) are constantly formed on ketoacid dehydrogenases and that the serine hydrolase ABHD11 acts as a glutaryl–lipoyl thioesterase that removes these lipoyl adducts. The OGDHc is particularly sensitive to lipoyl glutarylation, with ABHD11 inhibition impairing normal OGDHc catalysis and perturbing 2-oxoglutarate (2-OG) metabolism. We go on to show that rewiring 2-OG metabolism through ABHD11 inhibition yields distinct outcomes, depending on the cell type. Within cancer cells, ABHD11 inhibition predominantly leads to inactivation of 2-OGDDs involved in hypoxia-inducible factor (HIF) signaling and DNA methylation. In contrast, in primary human cytotoxic CD8^+^ T lymphocytes, which express high levels of ABHD11, 2-OGDD activity is preserved. Instead, ABHD11 inhibition and impaired OGDHc function alter fatty acid metabolism and CD8^+^ T cell differentiation, increasing the central memory T cell (T_CM_) pool.

## Results

### Glutaryl–lipoyl adducts accumulate with ABHD11 deficiency

**Fig. 1:**
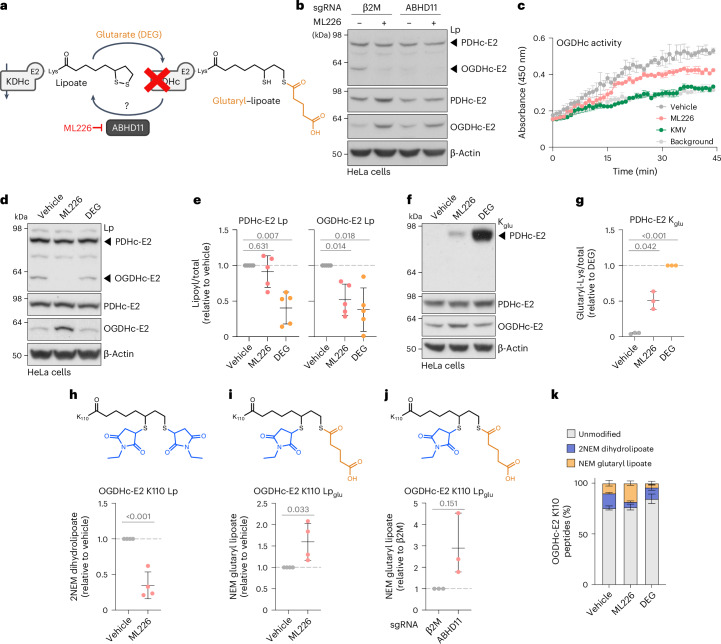
ABHD11 inhibition results in glutaryl-lipoylation of ketoacid dehydrogenases. **a**, Glutaryl adduct formation on the lipoylated E2 subunit of KDHc and its putative removal by ABHD11. **b**, Cas9-expressing HeLa cells were transduced with sgRNAs targeting β2M or ABHD11 for 11 days, then treated with 2.5 µM ML226 for 6 h. Lp modification of PDHc-E2 and OGDHc-E2 was determined using immunoblotting. Representative of *n* = 2 independent experiments. **c**, HeLa cells were treated with 2.5 µM ML226 for 6 h and OGDHc activity in whole lysates was assayed using a commercial kit in the presence or absence of 2.5 µM ML226 or 10 mM OGDHc inhibitor (KMV). Mean ± range of technical duplicates. Representative of *n* = 3 independent experiments. **d**–**g**, Lp and glutaryl modifications on KDHc. HeLa cells were treated with 1 µM ML226 or 1 mM DEG for 6 h (**d**,**e**) or 24 h (**f**,**g**), and Lp modification of PDHc-E2 and OGDHc-E2 was determined using immunoblotting (**d**). Visualization of glutaryl modifications using a K_glu_ antibody (**f**). Quantification of Lp (**e**) or K_glu_ (**g**) was normalized to total PDHc-E2 or OGDHC-E2 levels and adjusted to the vehicle condition or DEG. Mean ± s.d., *n* = 5 (**e**) and *n* = 3 (**g**) independent experiments; one-way ANOVA and Dunnett’s post-hoc test. **g**, Vehicle versus DEG, *P* < 0.0001. **h**–**k**, Effect of ABHD11 inhibition on OGDHc-E2 lipoylation. HeLa cells were treated with 2.5 µM ML226 for 6 h or 1 µM ML226 for 24 h (**h**,**i**,**k**), or Cas9-expressing HeLa cells were transduced with sgRNAs targeting β2M (control) or ABHD11 for 11 days (**j**). OGDHc-E2 was immunoprecipitated and K110 Lp (**h**) or K110 Lp_glu_ (**i**,**j**) were quantified using LC–MS/MS. Peptide abundance was normalized to the total abundance of OGDHc-E2 peptides and adjusted relative to the vehicle or β2M condition. Percentage of OGDHc-E2 K110 modified by Lp or Lp_glu_ (**k**). Mean ± s.d.; *n* = 3 (**j**,**k**) and *n* = 4 (**h**,**i**) independent experiments; unpaired *t*-test, two-tailed (**h**) *P* = 0.0004. KDHc, ketoacid dehydrogenases; Lp, lipoyl. Source data

ABHD11 is implicated in lipoylation of the OGDHc^16^, but how ABHD11 regulates this essential fatty acid modification is unclear. Given that glutaryl–lipoyl adducts form under conditions of glutarate excess on another TCA cycle ketoacid dehydrogenase, the PDHc^9^, we hypothesized that the major role of ABHD11 may be to reverse lipoyl glutarylation, maintaining TCA cycle integrity (Fig. 1a). We therefore explored whether ABHD11 inhibition or excess glutarate resulted in similar glutaryl and lipoyl modifications on TCA cycle ketoacid dehydrogenases.

We used ML226 as a selective and rapid inhibitor of ABHD11 (ref. ^19^). After just 6 h of ML226 treatment in HeLa cells, we were able to detect changes in lipoylation on ketoacid dehydrogenases, using an antibody that only detects the active, unmodified form of lipoate^16^. Direct comparison of ML226 treatment to lentiviral ABHD11 sgRNA depletion demonstrated that both pharmacological and genetic inhibition of ABHD11 reduced functional lipoylation on the E2 subunit of the OGDHc (dihydrolipoamide succinyltransferase (DLST), referred to as the OGDHc-E2) in preference to the E2 subunit of PDHc (dihydrolipoamide acetyltransferase (DLAT), referred to as the PDHc-E2; Fig. 1b). We also confirmed that 6 h of ML226 treatment in HeLa cells impaired OGDHc activity using a colorimetric assay (Fig. 1c), as previously reported for ABHD11 depletion^16^. Furthermore, 10 mM keto-β-methylvaleric acid (KMV) was used as a control to demonstrate complete inhibition of the OGDHc in vitro. Still, this compound was too toxic for use in cells to directly compare to ML226 treatment.

**Extended Data Fig. 1:**
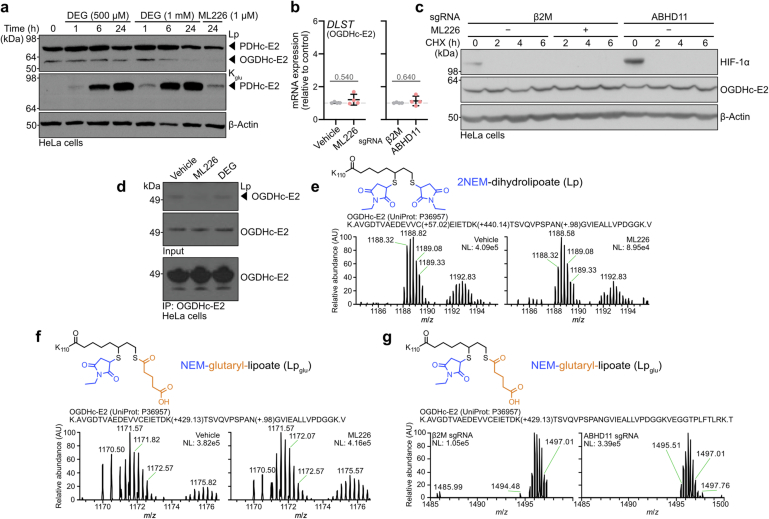
ABHD11 deficiency leads to the accumulation of glutaryl-lipoate adducts. **a**, Lp or K_glu_ immunoblot in HeLa cells treated with ML226 or DEG for the indicated times. Representative of *n* = 2 independent experiments. **b**, HeLa cells were treated with 1 µM ML226 for 24 h, or Cas9-expressing HeLa cells were transduced with sgRNAs targeting β2M (control) or ABHD11 for 11 days. *DLST* (OGDHc-E2) mRNA levels were determined using qRT-PCR. Transcript levels are normalized to β-actin *(ACTB)* and adjusted relative to the control condition. Mean ± s.d.; *n* = 4 independent experiments; one-way ANOVA and Dunnett’s post hoc test. **c**, Cas9-expressing HeLa cells were transduced as in (**b**) and treated with 10 µg/ml cycloheximide (CHX) with or without 2.5 µM ML226 for the indicated times. Levels of OGDHc-E2 were determined using immunoblotting, with HIF1α as a control. Representative of *n* = 4 independent experiments. **d**, OGDHc-E2 immunoprecipitation in HeLa cells for LC–MS/MS analysis of Lp modifications in Fig. 1h–k. Representative of *n* = 4 independent experiments. **e**–**g**, Representative chromatograms of OGDHc-E2 K110 Lp (**e**) in Fig. 1h; K110 Lp_glu_ (**f**) in Fig. 1i; and K110 Lp_glu_ (**g**) in Fig. 1j. Source data

We next compared whether ABHD11 inhibition or glutarate stress resulted in similar changes in lipoylation and glutarylation on ketoacid dehydrogenases. HeLa cells were treated with either ML226 or cell-permeable glutarate (diethylglutarate (DEG)), and lipoylation and glutaryl modifications were analyzed by immunoblot (Fig. 1d–g). Both ML226 and DEG treatment resulted in decreased functional lipoylation alongside detectable protein glutarylation, but they differed in their effects on the ketoacid dehydrogenases. DEG treatment decreased functional lipoylation on both the OGDHc-E2 and PDHc-E2 (Fig. 1d,e), with a corresponding increase in PDHc-E2 glutarylation (Fig. 1f,g). However, the effect of DEG treatment on these modifications differed over time, with 24 h DEG treatment required for substantive changes on lipoylation, although PDHc-E2 glutarylation was observed at 6 h (Extended Data Fig. 1a). ML226 treatment decreased OGDHc-E2 lipoylation with minimal effect on PDHc-E2 lipoylation (Fig. 1d,e). However, we still detected some PDHc-E2 glutarylation (Fig. 1f,g). ML226 sometimes appeared to increase total OGDH-E2 levels; however, we observed no effect of ML226 or ABHD11 depletion on expression of the OGDHc-E2 gene transcript (*DLST*), nor on OGDHc-E2 protein stability during a cycloheximide chase assay (Extended Data Fig. 1b,c).

Immunoblot detection of lipoylation and glutarylation relies on antibody specificity. While the detectable glutarylation following ML226 treatment suggested that ABHD11 may be involved in regulating this modification, the glutaryl antibody was raised against glutaryl-lysine, making it unlikely that it could reliably detect lipoyl-glutarylation species. Therefore, we used liquid chromatography–mass spectrometry (LC–MS)^9,16^ to detect whether ABHD11 inhibition altered protein (lysine glutarylation, referred to as K_glu_) or lipid (lipoyl-glutaryl, referred to as Lp_glu_) modifications. N-ethylmaleimide was used to block free thiols and preserve lipoyl moieties for detection.

**Extended Data Fig. 2:**
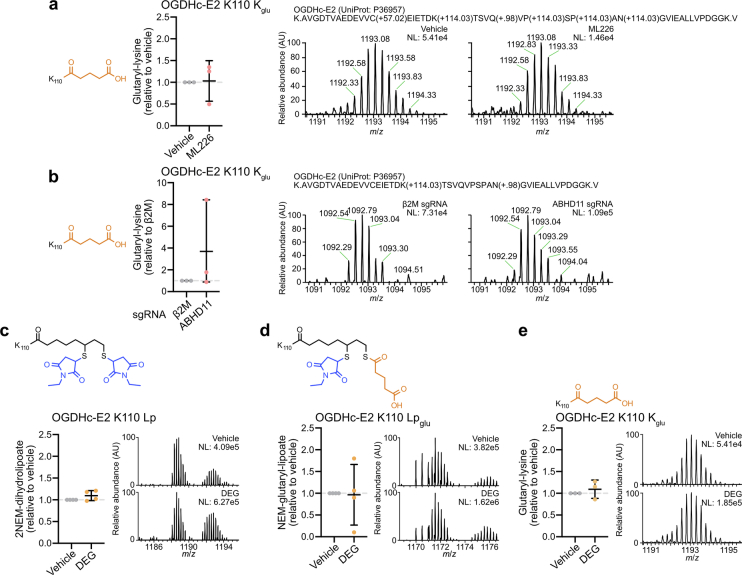
Lysine-glutarylation and lipoyl-glutarylation modifications following ABHD11 depletion or following glutarate pertubation. **a**,**b**, Quantification of OGDHc-E2 K110 lysine glutarylation (K_glu_) in ML226-treated cells from Fig. 1h,i (**a**), or in ABHD11 KO cells from Fig. 1j (**b**). Peptide abundance was normalized to the total abundance of modified peptides and adjusted relative to the vehicle or β2M condition. Chromatograms are representative. Mean ± s.d., *n* = 3 independent experiments. **c**–**e**, HeLa cells were treated with 1 mM DEG for 6 h or 24 h. OGDHc-E2 was immunoprecipitated and K110 Lp (**c**), K110 Lp_glu_ (**d**), or K110 K_glu_ (**e**) were quantified using LC–MS/MS. Control conditions are the same as in Fig. 1h,i and (**a**). Peptide abundance was normalized to the total abundance of OGDHc-E2 peptides and adjusted relative to the vehicle condition. Chromatograms are representative. Mean ± s.d., *n* = 3 (**e**) and *n* = 4 (**c**,**d**) independent experiments; unpaired *t*-test, two-tailed. Source data

We focused on the OGDHc-E2 given that the predominant effect of ABHD11 was on OGDHc activity, and we immunoprecipitated endogenous OGDHc-E2 from HeLa cells following treatment with ML226 for 6 h (Fig. 1h,i and Extended Data Fig. 1d). Lipoylation occurs on a single lysine residue within the OGDHc-E2, K110, and we observed that ML226 treatment decreased levels of the functional K110 lipoate moiety by LC–MS (Fig. 1h and Extended Data Fig. 1e). Conversely, K110 lipoyl-glutarylation (Lp_glu_) levels were increased by ML226 treatment (Fig. 1i and Extended Data Fig. 1f), as also seen with ABHD11 sgRNA-mediated depletion (Fig. 1j and Extended Data Fig. 1g, Supplementary Fig. 1a). In addition, ML226 inhibition increased Lp_glu_ on the PDHc-E2 (Supplementary Fig. 1c). LC–MS analysis of the PDHc is more complex, given the two lipoylated sites on the PDHc-E2 subunit, and we were only able to reproducibly detect lipoyl moieties on K259. Despite this limitation, we still observed a relative reduction in lipoylation and a concomitant increase in Lp_glu_ (Supplementary Fig. 1b,c). ABHD11 inhibition or depletion showed no reproducible effects on lysine glutarylation of the OGDHc-E2 (Extended Data Fig. 2a,b), consistent with ABHD11 regulating lipoyl glutarylation rather than lysine glutarylation.

Having demonstrated that ABHD11 regulated Lp_glu_, we examined the effects of glutarate excess on glutaryl modifications. We treated cells with DEG for 6 h and immunoprecipitated the OGDHc-E2 for analysis by LC–MS. We also asked if genetically increasing the glutaryl-CoA pool (the donor for glutarylation) altered glutaryl modifications and generated GCDH-deficient HeLa cells (Supplementary Fig. 1a). DEG did not substantially alter Lp, Lp_glu_ or K_glu_ levels after 6 h (Extended Data Fig. 2c–e), indicating that either DEG treatment for 6 h is insufficient to modify the OGDHc-E2, or that exogenous glutarate preferentially alters the PDHc, as previously reported^9^. K110-glutarylation and lipoyl glutarylation were detected in GCDH null cells, but the effects were variable, and GCDH depletion did not alter lipoylation or lysine glutarylation by immunoblot (Supplementary Figs. 1a and 2a–c). Moreover, the relative detection of K110_glu_ was low, even with DEG (Supplementary Fig. 2d). Therefore, although the K_glu_ antibody fortuitously detected a glutaryl signal following ML226 treatment (Fig. 1f), the major effect of ABHD11 inhibition is on glutaryl–lipoyl adducts rather than lysine glutarylation.

While LC–MS demonstrated that we could detect glutaryl-lipoylation, whether it reached levels to perturb the normal lipoate pool and inhibit OGDHc activity was unclear. We therefore used label-free quantification to analyze the relative abundance of lipoyl-modified or unmodified peptides spanning the OGDHc-E2 K110 residue with or without ML226 treatment (Fig. 1k). In untreated HeLa cells, the majority of OGDHc-E2 K110 peptide was not modified, and lipoylation only accounted for around 10–15% of the total peptide pool. Glutaryl-lipoylated K110 was detected in untreated cells, but following ML226 treatment, this increased to nearly 15% of the K110 peptide pool, with functional lipoylation reduced to around 5%. DEG treatment did not substantially change the functional lipoyl pool, which remained at around 10–15%. Therefore, the relatively low abundance of lipoylation in untreated cells suggests that lipoylation is rate-limiting for the OGDHc, and our findings are consistent with the accumulation of Lp_glu_ following ABHD11 inhibition, which reduces OGDHc activity (Fig. 1c).

### ABHD11 acts as a glutaryl–lipoyl thioesterase

The most likely explanation for increased Lp_glu_ following ML226 treatment was that the serine hydrolase activity of ABHD11 regulated the lipoyl adduct. We therefore developed several in vitro assays to determine if ABHD11 removed glutaryl adducts from the lipoylated OGDHc-E2.

**Fig. 2:**
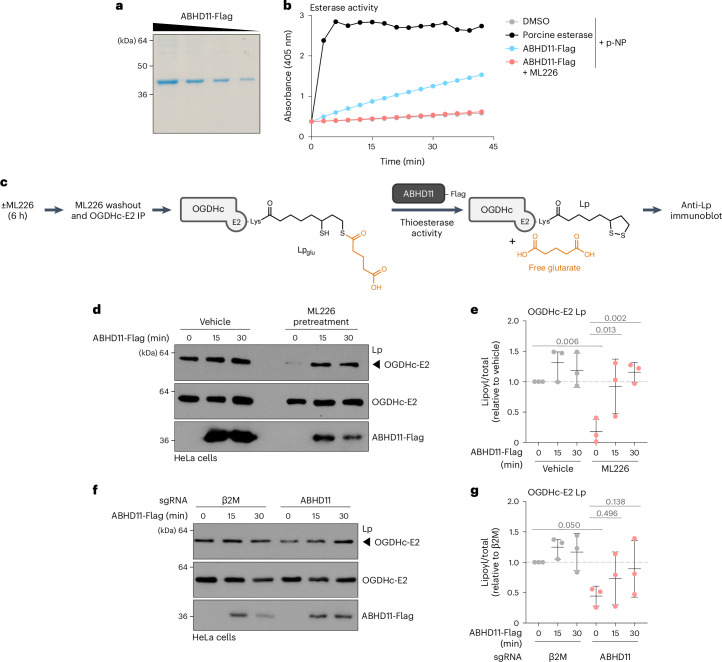
ABHD11 removes glutaryl adducts from lipoylated OGDHc-E2. **a**, Coomassie stain of ABHD11-Flag purified from HEK293T cells. Representative of *n* = 3 independent experiments. **b**, Generic esterase activity of ABHD11 using p-nitrophenyl acetate as a substrate. Porcine esterase was used as a control. Representative of *n* = 3 independent experiments. **c**–**g**, Purified ABHD11-Flag removes glutaryl–lipoate adducts from immunoprecipitated OGDHc-E2. Experimental method (**c**). HeLa cells were treated with 2.5 µM ML226 for 6 h (**d**,**e**), or Cas9-expressing HeLa cells were transduced with sgRNAs targeting β2M or ABHD11 for 11 days (**f**,**g**). OGDHc-E2 was immunoprecipitated and incubated with ABHD11-Flag for the indicated times, and Lp levels were measured using immunoblotting (**d**,**f**) and quantified by normalization to total OGDHc-E2 (**e**,**g**). Mean ± s.d.; *n* = 3 independent experiments; two-way ANOVA and Tukey’s post hoc test. Source data

We expressed and purified ABHD11-Flag in HEK293T cells and confirmed its hydrolase activity using a generic esterase substrate, p-nitrophenol acetate (Fig. 2a,b). We then measured whether purified ABHD11 could remove Lp_glu_ adducts from immunoprecipitated OGDHc, thereby restoring detection of functional lipoylation by immunoblot (Fig. 2c). We immunoprecipitated OGDHc-E2 Lp_glu_ adducts from HeLa cells treated with ML226, and then measured the levels of lipoate by immunoblot following short incubations of this immunoprecipitated OGDHc-E2 with purified ABHD11-Flag at 37 °C. ABHD11 restored lipoylation after 15 min, with full restoration by 30 min (Fig. 2d,e). Similar findings were observed following ABHD11 depletion using sgRNA (Fig. 2f,g).

**Extended Data Fig. 3:**
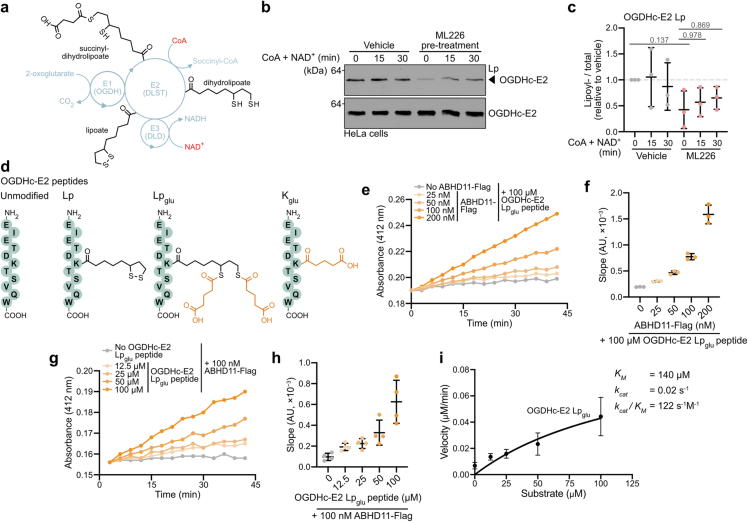
Glutaryl–lipoyl thioesterase activity of ABHD11. **a**, Schematic of OGDHc activity and lipoyl intermediates. **b**,**c**, Effect of NAD^+^ and CoA supplementation on functional lipoylation. HeLa cells were treated with 2.5 µM ML226 for 6 h. OGDHc-E2 was immunoprecipitated and incubated with 3 mM CoA and 0.5 mM NAD^+^ for the indicated times, and Lp levels were measured using immunoblotting (**b**) and quantified by normalization to total OGDHc-E2 (**c**). Mean ± s.d.; *n* = 3 independent experiments; two-way ANOVA and Tukey’s post-hoc test. **d**, Synthetic unmodified, Lp, Lp_glu_ or K_glu_ OGDHc-E2 peptides. **e**,**f**, Thioesterase activity of ABHD11 on OGDHc-E2 Lp_glu_ peptide (100 µM) at increasing enzyme concentrations. Mean ± s.d.; *n* = 3 independent experiments. **g**,**h**, Thioesterase activity of ABHD11 (100 nM) on OGDHc-E2 Lp_glu_ peptide at increasing substrate concentrations. Mean ± s.d.; *n* = 4 independent experiments. **i**, Michaelis–Menten kinetics of ABHD11 thioesterase activity on OGDHc-E2 Lp_glu_ peptide. Mean ± s.d.; *n* = 4 independent experiments. Source data

The OGDHc has an intrinsic capacity to remove lipoyl intermediates dependent on NAD^+^ and CoA (for example, succinyl-lipoate) (Extended Data Fig. 3a). We therefore tested whether supplementation with excess NAD^+^ and CoA restored lipoylation (Extended Data Fig. 3b,c). However, the addition of NAD^+^ and CoA did not restore functional lipoylation within 30 min, suggesting that while the intrinsic capacity of the OGDHc may resolve lipoyl adducts over time, ABHD11 restores functional lipoylation more rapidly.

**Fig. 3:**
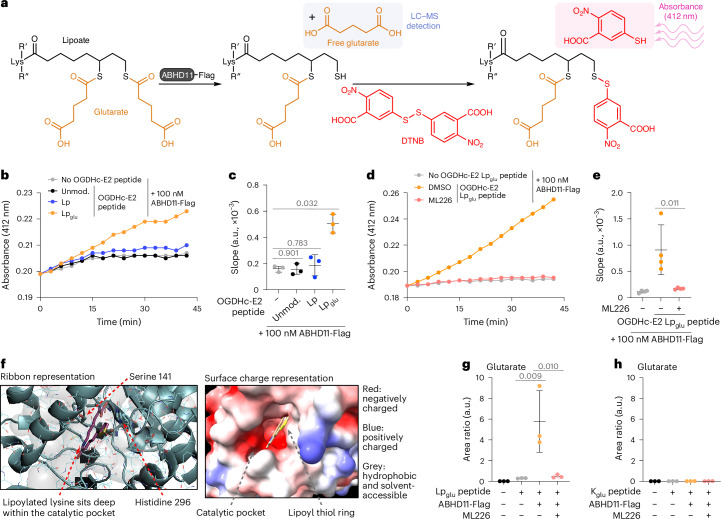
Glutaryl–lipoyl thioesterase activity of ABHD11. **a**, Schematic of assay to detect removal of glutaryl moieties from synthetic OGDHc-E2 peptides by ABHD11-Flag. Thioesterase activity is measured by the release of free thiols, which react with Ellman’s reagent (DTNB) and can be detected via absorbance at 412 nm. Alternatively, glutarate release is detected using LC–MS. **b**,**c**, Thioesterase activity of ABHD11-Flag (100 nM) on unmodified, Lp or Lp_glu_ OGDHc-E2 peptides (100 µM) was determined via DTNB assay. Mean ± s.d.; *n* = 3; one-way ANOVA and Dunnett’s post hoc test. **d**,**e**, Thioesterase activity of ABHD11-Flag (100 nM) on OGDHc-E2 Lp_glu_ peptide (100 µM) in the presence or absence of ML226 (2.5 µM) was determined via DTNB assay. Mean ± s.d., *n* = 4 independent experiments, one-way ANOVA and Tukey’s post hoc test. **f**, In silico modeling of ABHD11 to a marine α/β-hydrolase fold esterase (RCSB PDB ID: 7c4d.1; 2.03 Å) with lipoate moiety. Catalytic S141 and H296 indicated. Ribbon (left) and surface charge (right) models shown. **g**,**h**, Glutarate release from OGDHc-E2 Lp_glu_ (**g**) or K_glu_ (**h**) peptides (100 µM) incubated with ABHD11-Flag (100 nM) in the presence or absence of ML226 (2.5 µM) was determined using LC–MS. Mean ± s.d., *n* = 3 independent experiments, one-way ANOVA and Dunnett’s post hoc test. RCSB PDB, Research Collaboratory for Structural Bioinformatics Protein Data Bank. Source data

Next, to determine if ABHD11 acted directly on the Lp_glu_ adduct in an enzymatic manner, we measured the activity of purified ABHD11 against a recombinant Lp_glu_ OGDHc-E2 peptide encompassing the minimal residues around the lipoylated K110 (Extended Data Fig. 3d). Thioesterase activity was measured by the addition of Ellman’s reagent (5,5′-dithiobis-(2-nitrobenzoic acid) (DTNB)) at 37 °C. This reagent provides a colorimetric readout when it binds to exposed free thiols, thereby detecting the lipoate moiety if an adduct is removed (Fig. 3a). ABHD11 increased the availability of free thiols on the Lp_glu_ peptide compared to the vehicle control (Fig. 3b,c). ABHD11 had no effect on the unmodified peptide, nor a peptide with the lipoate thiol ring in an oxidized state (Fig. 3b,c and Extended Data Fig. 3d). The thioesterase activity of ABHD11 against the glutaryl moiety was dependent on enzyme and substrate concentration (Extended Data Fig. 3e–i) and was inhibited by ML226 (Fig. 3d,e).

**Extended Data Fig. 4:**
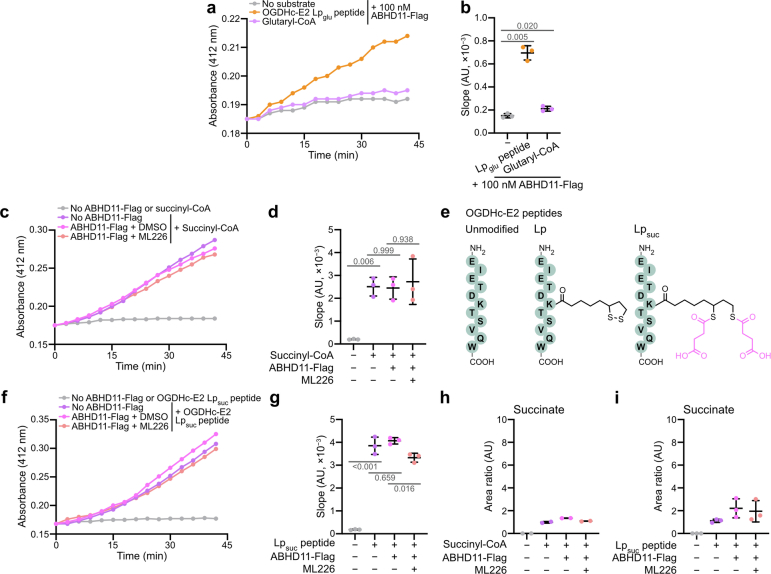
Thioesterase activity of ABHD11 on glutaryl and succinyl esters. **a**,**b**, Thioesterase activity of ABHD11-Flag (100 nM) on OGDHc-E2 Lp_glu_ peptide (100 µM) compared to glutaryl-CoA (100 µM) was determined via DTNB assay. Mean ± s.d.; *n* = 3 independent experiments, one-way ANOVA and Tukey’s post-hoc test. **c**,**d**, Thioesterase activity of ABHD11-Flag (100 nM) on succinyl-CoA (100 µM) in the presence or absence of ML226 (2.5 µM) was determined via DTNB assay. Mean ± s.d.; *n* = 3 independent experiments; one-way ANOVA and Dunnett’s post-hoc test. **e**, Synthetic Lp_suc_ OGDHc-E2 peptide. **f**,**g**, Thioesterase activity of ABHD11-Flag (100 nM) on OGDHc-E2 Lp_suc_ peptide (100 µM) in the presence or absence of ML226 (2.5 µM) was determined via DTNB assay. Mean ± s.d.; *n* = 3 independent experiments; one-way ANOVA and Dunnett’s post hoc test. (**g**) Column 1 vs column 2: *P* < 0.0001. **h**,**i**, Succinate release from succinyl-CoA (100 µM) (**h**) or OGDHc-E2 Lp_suc_ peptide (100 µM) (**i**) incubated with ABHD11-Flag (100 nM) in the presence or absence of ML226 (2.5 µM) was determined using LC–MS. Mean ± s.d.; *n* = 2 (**h**), *n* = 3 (**i**) independent experiments; one-way ANOVA and Dunnett’s post-hoc test. Source data

We then examined whether ABHD11 was selective for glutaryl–lipoyl adducts or could hydrolyze other related acyl esters. Glutaryl-CoA is the most abundant related glutaryl-ester in cells, but ABHD11 did not show any specificity against glutaryl-CoA (Extended Data Fig. 4a,b). We also measured ABHD11 activity against succinyl-CoA, but this compound underwent spontaneous hydrolysis in the DTNB assay (Extended Data Fig. 4c,d). Succinyl-CoA self-hydrolysis is well recognized^20^; however, the addition of ABHD11 had no additional effect on succinyl-CoA cleavage (Extended Data Fig. 4c,d). ABHD11’s specificity for lipoyl adducts was consistent with substrate docking predictions, where we modeled the enzymatic cleft of ABHD11 based on the crystal structure of a related marine α/β hydrolase^21^ (Fig. 3f). The lipoate moiety docked within the catalytic pocket, proximal to the catalytic serine-141 and histidine-296 residues (Fig. 3f). However, CoA could not be modeled within the enzymatic pocket. Lastly, we examined if ABHD11 could hydrolyze a different acyl chain from lipoate and used a synthetic succinyl-lipoyl (Lp_suc_) OGDHc-E2 peptide (Extended Data Fig. 4e). The Lp_suc_ peptide was unstable, similarly to succinyl-CoA, but there was a nonsignificant increase in hydrolysis with ABHD11 and some inhibition with ML226 treatment (Extended Data Fig. 4f,g).

While the DTNB assay confirmed that ABHD11 hydrolyzed the glutaryl–lipoyl bond, it was unclear whether glutarate was released by ABHD11 or transferred to another moiety. Therefore, we used LC–MS to measure if ABHD11 released glutarate from glutaryl–lipoyl adducts (Fig. 3a). ABHD11 released glutarate from the OGDHc-E2 Lp_glu_ peptide, and this was blocked with ML226 (Fig. 3g). No free glutarate was detected when ABHD11 was incubated with a glutarylated lysine OGDHc-E2 peptide (K_glu_; Fig. 3h and Extended Data Fig. 3d), consistent with ABHD11 specificity for lipoyl adducts. Lastly, we examined if we could detect succinate release following the addition of ABHD11 to succinyl esters (Lp_suc_ and succinyl-CoA). ABHD11 mildly increased the levels of free succinate, and this was slightly decreased with ML226 treatment (Extended Data Fig. 4h,i). However, these assays were difficult to interpret due to the high levels of spontaneous hydrolysis with the succinyl esters. Therefore, while it remains possible that ABHD11 can hydrolyze other acyl-lipoyl esters, the predominant effect we observe is against glutarylation of the OGDHc lipoate arm.

### Metabolic outcomes of ABHD11 inhibition in HeLa cells

We next examined the functional outcomes of ABHD11 inhibition in different cell types. Prior studies in cancer cell lines showed that ABHD11 deficiency leads to 2-OG accumulation, which drives the formation of the l-enantiomeric (*S)* form of 2-hydroxyglutarate (2-HG) and subsequent inactivation of 2-OGDDs involved in the HIF pathway (prolyl hydroxylases, PHDs), histone marks (for example, lysine demethylases, KDMs) and DNA methylation (for example, ten-eleven translocation,TET enzymes)^16,22,23^. However, the effects of perturbing ABHD11 function in primary cells are unexplored. Alternatively, in CD8^+^ T cells, excess glutarate has pleotropic effects on metabolism, altering Lp_glu_, K_glu_ and also competitively inhibiting 2-OGDDs^9^. However, whether glutarate treatment has similar outcomes in cancer cells is not known. Therefore, to experimentally compare the outcomes of ABHD11 inhibition to glutarate accumulation, we used HeLa cells as a well-studied cancer cell line, alongside primary human CD8^+^ T lymphocytes, where the effects of DEG have been well characterized^9^.

We first determined whether ML226 and DEG had similar effects on 2-OGDDs and examined PHD activity (HIF-1α stabilization), TET activity (DNA 5-hydroxymethylcytosine (5hmC) levels) and KDM activity (histone lysine methylation marks). Dimethyloxalyl glycine (DMOG) was used as a control for broad-spectrum 2-OGDD inhibition. We also used cell-permeable 2-OG (dimethyl 2-OG, DM-2OG), as a control for 2-OG accumulation driving 2-HG formation, as excess 2-OG has been previously shown to drive PHD inhibition via L-2-HG^22,24^.

**Fig. 4:**
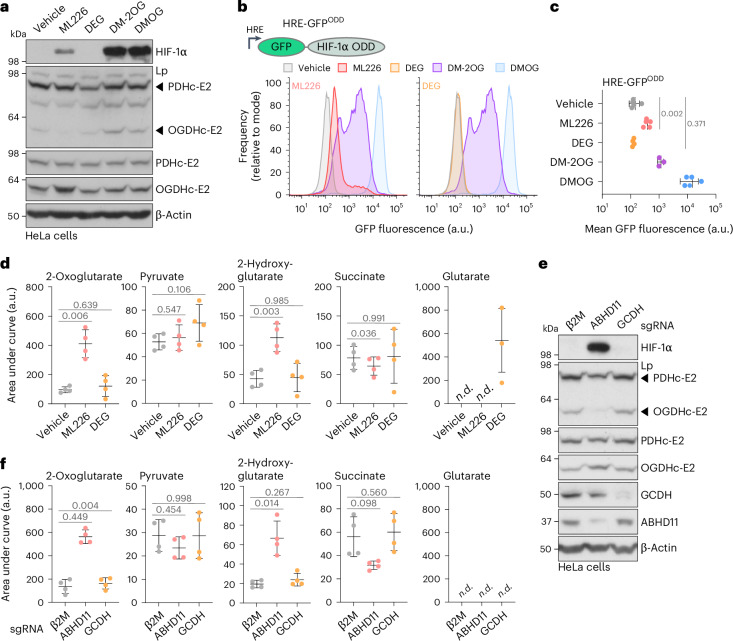
ABHD11 inhibition has distinct outcomes compared to perturbing cellular glutarate levels. **a**–**c**, Comparison of ML226 with DEG or DM-2OG treatment on HIF-1α stabilization and activity. **a**, HeLa cells were treated with 2.5 µM ML226, 1 mM DEG or 6 mM DM-2OG for 6 h. DMOG (1 mM) was used as a control. HIF-1α levels were determined by immunoblotting. Representative of *n* = 3 independent experiments. **b**,**c**, HeLa HRE-GFP^ODD^ cells were treated with 1 µM ML226, 1 mM DEG, 6 mM DM-2OG or 1 mM DMOG for 24 h and GFP levels were determined using flow cytometry. For visualization purposes, ML226 and DEG conditions are plotted separately. Quantification of mean GFP fluorescence (**c**). Mean ± s.d., *n* = 3 (DM-2OG), *n* = 4 (DEG) and *n* = 5 (ML226, DMOG) independent experiments; one-way ANOVA and Dunnett’s post-hoc test. **d**, HeLa cells were treated with 1 µM ML226 or 1 mM DEG for 24 h, and the indicated metabolites were quantified using LC–MS. Mean ± s.d., *n* = 4 independent experiments, one-way ANOVA and Dunnett’s post hoc test. **e**,**f**, Cas9-expressing HeLa cells were transduced with sgRNA targeting β2M, ABHD11 or GCDH for 11 days. **e**, HIF-1α and Lp modifications were determined using immunoblotting. Representative of *n* = 4 independent experiments. **f**, The indicated metabolites were quantified using LC–MS. Mean ± s.d., *n* = 4 independent experiments, one-way ANOVA and Dunnett’s post hoc test. n.d., not detected. Source data

**Extended Data Fig. 5:**
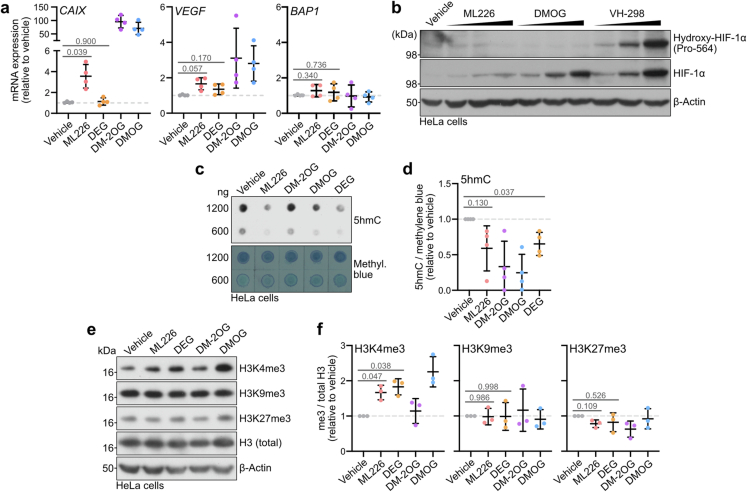
ABHD11 inhibition has distinct outcomes compared to perturbing cellular glutarate levels. **a**, HeLa cells were treated with 1 µM ML226, 1 mM DEG, 6 mM DM-2OG, or 1 mM DMOG for 24 h, and *CAIX*, *VEGF* and *BAP1* mRNA levels were determined using qRT-PCR. *BAP1* is non-HIF target gene. Transcript levels are normalized to *ACTB* and adjusted relative to the vehicle condition. Mean ± s.d.; *n* = 4 independent experiments; one-way ANOVA and Dunnett’s post-hoc test. **b**, HeLa cells were treated with increasing doses of ML226 (0.25 µM, 1 µM, 2.5 µM) for 6 h, or DMOG (100 mM, 250 mM, 500 mM) or VH-298 (2.5 µM, 10 µM, 25 µM) for 2 h. Levels of Pro-564-hydroxylated HIF1α and total HIF1α were determined using immunoblotting. Representative of *n* = 2 independent experiments*.* **c**,**d**, HeLa cells were treated with 2.5 µM ML226, 1 mM DEG, 6 mM DM-2OG, or 1 mM DMOG for 24 h. 5hmC levels were determined using dot blotting of genomic DNA (**c**), normalized to total DNA staining by methylene blue and adjusted relative to the vehicle condition (**d**). Mean ± s.d.; *n* = 4 independent experiments; one-way ANOVA and Dunnett’s post-hoc test. **e**,**f**, HeLa cells were treated as in (**c**,**d**) and H3K4me3, H3K9me3 and H3K27me3 levels were determined using immunoblotting (**e**). Methylation levels were normalized to total histone H3 levels and adjusted relative to the vehicle condition (**f**). Mean ± s.d.; *n* = 3 independent experiments; one-way ANOVA and Dunnett’s post-hoc test. Source data

ML226 treatment resulted in HIF-1α stabilization, upregulation of HIF-1 target genes (*CAIX* and *VEGF*), and activation of a HIF-1 fluorescent reporter (HRE-GFP^ODD^) in HeLa cells, similar to treatment with cell-permeable (DM-2OG; Fig. 4a–c and Extended Data Fig. 5a). ML226 also stabilized HIF-1α in a nonprolyl-hydroxylated form (Extended Data Fig. 5b), and decreased TET activity (indicated by lower levels of 5hmC; Extended Data Fig. 5c,d), consistent with 2-HG mediated inhibition of 2-OGDDs, as previously reported^22,24^. However, ABHD11 inhibition had mixed effects on KDM activity, and only histone 3 lysine 4 trimethylation (H3K4me3) was increased, indicating that the effect of ABHD11 inhibition on 2-OGDDs is not uniform across this enzyme group (Extended Data Fig. 5e,f).

Cell-permeable 2-OG treatment partially phenocopied the effects of ML226, consistent with perturbed OGDHc and 2-OG accumulation stabilizing HIF-1α and decreasing 5hmC levels (Fig. 4a–c and Extended Data Fig. 5a,c–f). However, DM-2OG did not have any substantial effects on histone marks. DEG treatment decreased 5hmC and increased H3K4me3, similarly to ML226 treatment, but had no effect on HIF-1α stabilization (Fig. 4a–c and Extended Data Fig. 5a,c–f). These findings suggested that DEG may not drive 2-HG formation, as occurs with decreased OGDHc function^22,24^. Indeed, when we undertook LC–MS analysis of TCA cycle metabolites, ML226 treatment resulted in the accumulation of 2-OG and 2-HG, but this did not occur following DEG treatment, which increased only free glutarate (Fig. 4d).

We also compared the metabolic and phenotypic consequences of genetically impairing 2-OG or glutarate metabolism, and depleted HeLa cells of ABHD11 or GCDH (Fig. 4e). LC–MS analysis of TCA cycle metabolites showed that ABHD11 loss resulted in 2-OG and 2-HG accumulation as expected^16,23^, and in line with the effects of ML226 (Fig. 4f). GCDH loss did not alter 2-OG metabolism, nor 2-HG levels. Moreover, free glutarate was not detected in GCDH-deficient cells, indicating that HeLa cells can metabolically adapt to avoid excess glutaryl-CoA. The effects of ABHD11 and GCDH depletion on 2-OGDD activity were mostly consistent with ML226 and DEG treatment. ABHD11 loss resulted in increased HIF-1α levels and decreased 5hmC, similarly to ML226 inhibition, whereas GCDH depletion only decreased 5hmC levels (Fig. 4e and Supplementary Fig. 3a,b). However, the effect of ABHD11 or GCDH depletion differed from ML226 and DEG with respect to histone marks. GCDH loss did not alter levels of histone methylation marks, but both H3K4me3 and H3K9me3 were decreased following ABHD11 depletion (Supplementary Fig. 3c,d), likely reflecting compensatory mechanisms with genetic perturbations compared to shorter-term drug treatments. Despite these differences, the consistent findings were that ABHD11 depletion or inhibition predominantly results in 2-OG and 2-HG accumulation and HIF-1 activation, which differed from conditions of glutarate excess.

### Metabolic outcomes of ABHD11 inhibition in CD8^+^ T cells

Metabolism is markedly different between immortalized cancer cells and primary cells, and while the major effect of ABHD11 inhibition in HeLa cells was on 2-OG metabolism and 2-OGDD activity, the downstream consequences of perturbing this glutaryl–lipoyl pathway in primary cells may differ from immortalized cancer cells. In particular, whether impaired OGDHc activity would drive inhibition of 2-OGDDs was not known in primary cells. Moreover, we sought to investigate how ABHD11 may influence cell function in other ways.

**Fig. 5:**
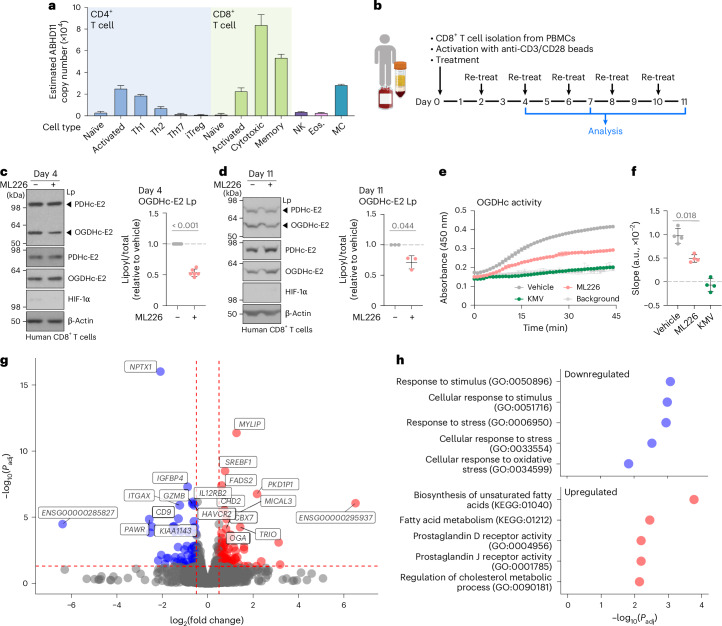
ABHD11 inhibition reduces OGDHc activity and alters fatty acid metabolism in CD8^+^ T cells. **a**, Protein copy number of ABHD11 in hematopoietic cell proteomes from the ImmPRes database. Mean + s.d. values obtained directly from ImmPRes database. **b**, Timeline of treatment. Human CD8^+^ T cells were activated with anti-CD3/CD28 Dynabeads and continuously treated with 1 µM ML226 for 11 days. Panel **b** is created with BioRender.com. **c**,**d**, Lp modifications on OGDHc-E2 after 4 (**c**) or 11 (**d**) days of ML226 treatment were determined using immunoblotting (representative blots shown). Quantification of Lp was normalized to total OGDHc-E2 levels and adjusted to the vehicle condition. Each data point represents one donor. Mean ± s.d., *n* = 6 (**c**) and *n* = 3 (**d**) donors; paired two-tailed *t*-test. **c**, *P* < 0.0001. **e**,**f**, OGDHc activity in whole lysates was assayed after 11 days using a commercial kit in the presence or absence of 2.5 µM ML226 or 10 mM KMV. Technical duplicates in a representative experiment (**e**) Mean ± range. Specific OGDHc activity (**f**), with each data point representing one donor. Mean ± s.d., *n* = 4 donors, paired two-tailed *t*-test. **g**, Volcano plot of differentially expressed genes between ML226-treated and untreated CD8^+^ T cells as determined by RNA-seq analysis (average of four independent donors). Differential expression analysis was performed with DESeq2. *P*-values obtained with the Wald test were adjusted for multiple comparisons using the procedure of Benjamini–Hochberg. **h**, Functional enrichment analysis of RNA-seq data using g:Profiler2 with sources GO:BP, GO:MF, KEGG and REAC. *P*-values were calculated and corrected from set intersections using the Set Counts and Sizes (g:SCS) method. NK, natural killer; Eos., eosinophil; MC, mast cell. Source data

CD8^+^ T cells were an obvious choice to explore the role of ABHD11, given the prior identification of glutarylation affecting CD8^+^ T cell function^9^. Moreover, using published hematopoietic cell proteomes, we noted that ABHD11 was dynamically expressed in T cells and particularly CD8^+^ T cells^25^, with low ABHD11 levels in naive CD4^+^ and CD8^+^ T cells, but high expression following T cell receptor activation (Fig. 5a). CD8^+^ T cells expressed higher ABHD11 than any CD4^+^ T cell population, and memory CD8^+^ T cells expressed less ABHD11 than cytotoxic CD8^+^ T cells. Therefore, we explored whether ABHD11 inhibition altered OGDHc activity in CD8^+^ T cells, as observed in cancer cell lines.

**Extended Data Fig. 6:**
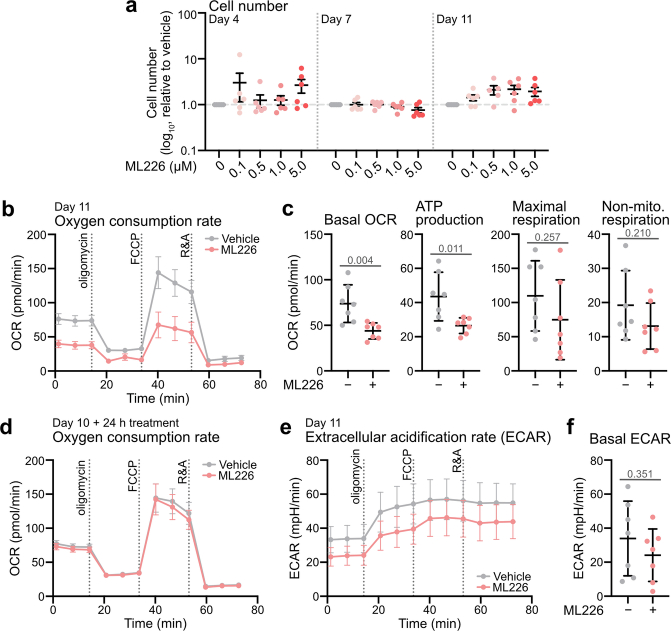
Effect of ABHD11 inhibition on CD8^+^ T cell metabolism. **a**–**c**, Human CD8^+^ T cells were isolated from healthy donor peripheral blood mononuclear cells, activated with anti-CD3/CD28 Dynabeads and continuously treated with 1 µM ML226 for 11 days. Cell numbers were quantified at 4, 7 and 11 days, and normalized to the vehicle control for each time point. Each data point represents one donor. Mean ± s.e.m.; *n* = 6 donors; two-way ANOVA and Dunnett’s post-hoc test. **b**,**c**, Oxygen consumption rate (OCR) after 11 days during a mitochondrial stress test (1 μM oligomycin, 1.5 μM FCCP, 100 nM rotenone + 1 μM antimycin) was quantified using a Seahorse XFe96 Extracellular Flux Analyzer (**b**). Mean ± s.e.m.; *n* = 6 donors. Quantification of basal OCR, ATP production, maximal respiration and nonmitochondrial respiration (**c**). Each data point represents one donor. Mean ± s.d.; *n* = 6 donors; unpaired two-tailed *t*-test. **d**, Activated CD8^+^ T cells were cultured for 10 days and treated with 1 µM ML226 for 24 h. OCR during a mitochondrial stress test (1 μM oligomycin, 1.5 μM FCCP, 100 nM rotenone + 1 μM antimycin). Mean ± s.e.m.; *n* = 4 donors. **e**,**f**, Activated human CD8^+^ T cells were continuously treated with 1 µM ML226 for 11 days. Extracellular acidification (ECAR) during a mitochondrial stress test (**e**). Mean ± s.e.m.; *n* = 6 donors. Quantification of basal ECAR (**f**). Each data point represents one donor. Mean ± s.d.; *n* = 6 donors; unpaired two-tailed *t*-test. Source data

CD8^+^ T cells were isolated from healthy human donor peripheral blood mononuclear cells, activated using anti-CD3/CD28 Dynabeads, and continuously cultured with ML226 (Fig. 5b). ML226 was well tolerated up to 5 µM, without affecting cell growth (Extended Data Fig. 6a). ML226 treatment demonstrated the expected outcomes on lipoylation and OGDHc activity, with reduced functional OGDHc-E2 lipoylation and impaired OGDHc activity (Fig. 5c–f).

We next examined how ML226 altered CD8^+^ T cell mitochondrial function. ML226 reduced oxidative phosphorylation, as expected, but prolonged inhibition of ABHD11 was required to induce this reduction, as short-term treatment of CD8^+^ T cells (24 h) did not alter the oxygen consumption rate (OCR; Extended Data Fig. 6b–d). We found no significant changes in extracellular acidification or glycolysis (measured by a standard glycolysis stress test) in CD8^+^ T cells treated with ML226 (Extended Data Fig. 6e,f and Supplementary Fig. 4a–d), indicating again that ML226 has a predominant effect on OGDHc activity rather than PDHc activity. ML226 treatment did not substantially alter mitochondrial content (MitoTracker), superoxide levels (MitoSox), or mitochondrial membrane potential (TMRM; Supplementary Fig. 5a–f).

**Extended Data Fig. 7:**
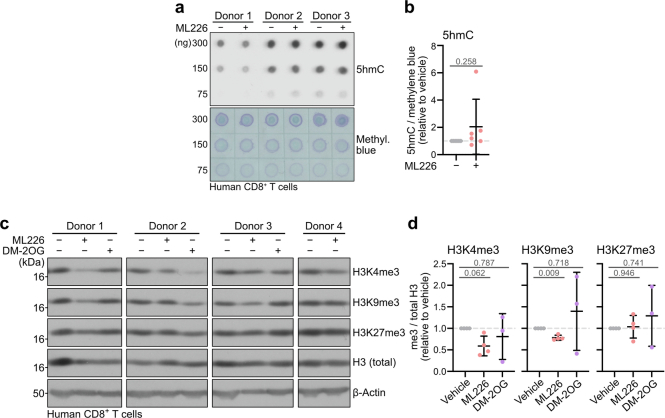
Effect of ABHD11 inhibition on DNA hydroxymethylation and selected histone marks. **a**,**b**, Human CD8^+^ T cells were isolated from healthy donor peripheral blood mononuclear cells, activated with anti-CD3/CD28 Dynabeads and continuously treated with 1 µM ML226 for 11 days. 5hmC levels were determined using dot blotting of genomic DNA (**a**), normalized to total DNA staining by methylene blue and adjusted relative to the vehicle condition (**b**). Each data point represents one donor. Mean ± s.d.; *n* = 6 donors; paired two-tailed *t*-test. **c**,**d**, Human CD8^+^ T cells were isolated from healthy donor peripheral blood mononuclear cells, activated with anti-CD3/CD28 Dynabeads and continuously treated with 1 µM ML226 or 1 mM DM-2OG for 11 days. H3K4me3, H3K9me and H3K27me3 levels were determined using immunoblotting (**c**). Methylation levels were normalized to total histone H3 levels and adjusted relative to the vehicle condition (**d**). Each data point represents one donor. Mean ± s.d.; *n* = 4 (ML226), *n* = 3 (DM-2OG) donors; one-way ANOVA and Dunnett’s post-hoc test. Source data

As the predominant effect of ML226 treatment was on OGDHc activity, we next asked if perturbing OGDHc function in CD8^+^ T cells indirectly inhibits 2-OGDDs, as observed in HeLa cells. However, no changes in 5hmC levels were observed (Extended Data Fig. 7a,b). Moreover, ML226 did not appear to alter HIF-1α levels or inhibit KDM activity (Fig. 5c,d and Extended Data Fig. 7c,d). Instead, we observed a slight reduction in H3K4me3 and H3K9me3 following 11 days of treatment, indicating that despite ABHD11 inhibition leading to decreased OGDHc activity, there was no evidence that 2-OGDDs were inhibited.

Given the differential effects of ML226 on 2-OGDD inhibition, we wanted to take an orthogonal approach to impairing OGDHc activity. We attempted direct inhibition with KMV, but this was toxic to CD8^+^ T cells at concentrations needed to inhibit the OGDHc, as also observed in HeLa cells. Instead, we used DM-2OG treatment to increase the cellular 2-OG pool as a surrogate for blocking the OGDHc. DM2-OG, like ML226 treatment, had no consistent effects on histone marks in CD8^+^ T cells (Extended Data Fig. 7c,d).

CD8^+^ T cells treated with ML226 appeared to be able to rewire 2-OG metabolism, without major effects on 2-OGDD activity. To better understand the effects of ABHD11 inhibition in CD8^+^ T cells, we undertook RNA-seq analysis following ML226 treatment. The major effect of ABHD11 inhibition was on fatty acid metabolism (Fig. 5g,h), with marked changes in the expression of genes involved in unsaturated fatty acid and cholesterol synthesis (Fig. 5g,h). Fatty acid synthesis and fatty acid oxidation are known to be important for memory T cell maintenance, with effector T cells being more reliant on glycolysis for ATP generation^26–28^. We also observed decreased mRNA expression of some T cell effector markers (for example, *GZMC*).

**Extended Data Fig. 8:**
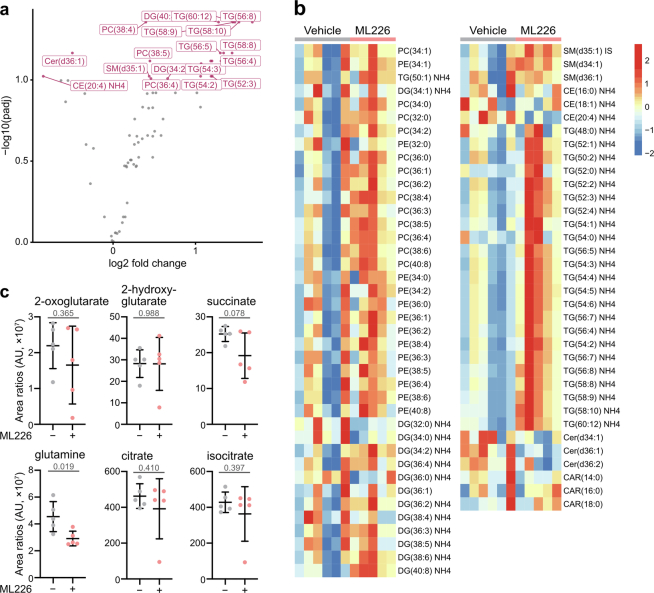
Metabolomic analyses in CD8^+^ T cells treated with ML226. **a**,**b**, Human CD8^+^ T cells were isolated from healthy donor peripheral blood mononuclear cells, activated with anti-CD3/CD28 Dynabeads and continuously treated with 1 µM ML226 for 11 days. Metabolites were extracted and quantified using LC–MS. **a**, Volcano plot of differentially expressed nonpolar metabolites between ML226-treated and untreated CD8^+^ T cells as determined by targeted LC–MS. *P*-values for the group effect were extracted from the model coefficients and significance was determined using false discovery rate correction (Benjamini–Hochberg method), with adjusted *P*-value < 0.05 and absolute log_2_ fold change > log_2_(1.25). **b**, Heatmap of metabolites in (**a**). **c**, Polar metabolite analysis. Mean ± s.d.; *n* = 5 donors; one-way ANOVA and Dunnett’s post-hoc test. Source data

We next used LC–MS to see if we could detect changes in fatty acid synthesis following ABHD11 inhibition in CD8^+^ T cells. After 11 days of ML226 treatment, we observed a marked increase in unsaturated tri- and diglycerides, with a downregulation of saturated glycerides (Extended Data Fig. 8a,b). It was therefore likely that by blocking the OGDHc, 2-OG was driving fatty acid synthesis into unsaturated tri- and diglycerides via reductive metabolism, rather than resulting in an accumulation of 2-HG. Indeed, LC–MS analysis of polar metabolites demonstrated no increase in 2-HG levels (Extended Data Fig. 8c), consistent with metabolic rewiring into fatty acid synthesis (via citrate), rather than 2-HG. The low levels of 2-HG also explained why 2-OGDD activity was preserved following ABHD11 inhibition in CD8^+^ T cells.

### ABHD11 influences human CD8^+^ T cell differentiation

**Fig. 6:**
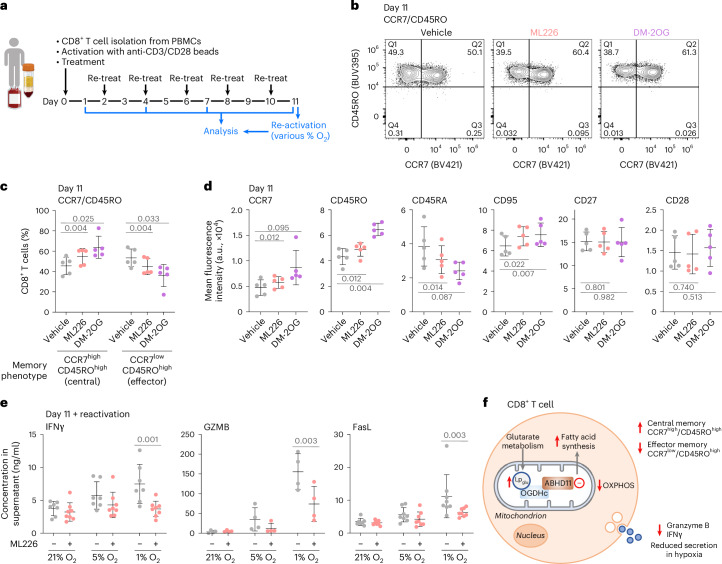
ABHD11 influences human CD8^+^ T cell differentiation. **a**–**d**, Human CD8^+^ T cells were isolated from healthy donor peripheral blood mononuclear cells, activated with anti-CD3/CD28 Dynabeads, and continuously treated with 1 µM ML226 or 1 mM DM-2OG for 11 days. **a**, Timeline of treatment. Panel **a** is created with BioRender.com. **b**,**c**, CCR7 and CD45RO levels after 11 days were determined using flow cytometry. **b**, Representative flow cytometry dot plots. **c**, Percentage of CCR7^high^CD45RO^high^ (central memory) and CCR7^low^CD45RO^high^ (effector memory). Each data point represents one donor. Mean ± s.d.; *n* = 5 donors; one-way ANOVA and Dunnett’s post-hoc test per subpopulation. **d**, CCR7, CD45RO, CD45RA, CD95, CD27 and CD28 levels after 11 days were determined using flow cytometry. Each data point represents one donor. Mean ± s.d., *n* = 5 donors, one-way ANOVA and Dunnett’s post hoc test. **e**, Following 11 days of culture with ML226, CD8^+^ T cells were counted, acclimatized to different oxygen tensions as indicated for 2 h, reactivated with anti-CD3/CD28 Dynabeads and cytokine levels in the media 4 h postreactivation were determined using a commercial multiplexed assay and flow cytometry. Mean ± s.d., *n* = 8 (IFNγ, FasL) and *n* = 4 (granzyme B) donors, two-way ANOVA and Dunnett’s post hoc test. **f**, Effect of ABHD11 inhibition on CD8^+^ T cells. ABHD11 inhibition reduces OGDHc activity via Lp_glu_ accumulation on the OGDHc-E2. This results in impaired OXPHOS and is associated with a phenotypic switch to central memory CD8^+^ T cells, with reduced secretion of IFNγ and granzyme B under hypoxic conditions. Source data

**Extended Data Fig. 9:**
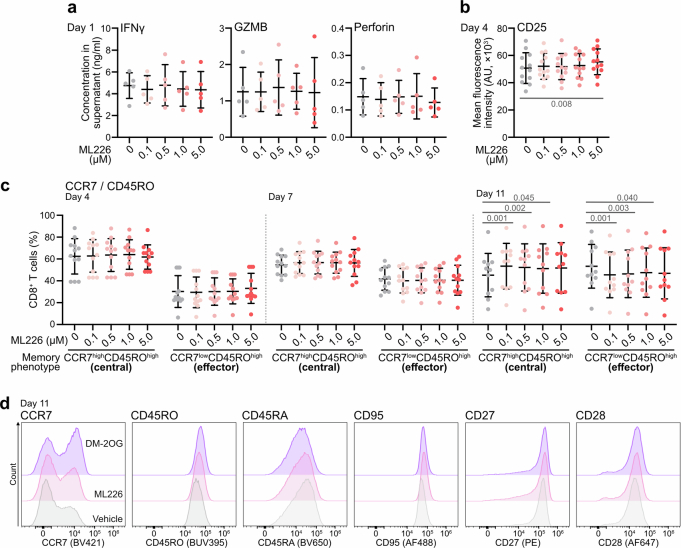
Effect of ABHD11 inhibition on CD8^+^ T cell differentiation. **a**–**d**, Human CD8^+^ T cells were isolated from healthy donor peripheral blood mononuclear cells, activated with anti-CD3/CD28 Dynabeads and continuously treated with the indicated concentrations of ML226 or 1 mM DM-2OG for 11 days. **a**, Cytokine levels in cell culture supernatants after 1 day were determined using a commercial multiplexed assay and flow cytometry. Each data point represents one donor. Mean ± s.d.; *n* = 5 donors; one-way ANOVA and Dunnett’s post-hoc test. **b**, CD25 levels after 4 days were determined using flow cytometry. Each data point represents one donor. Mean ± s.d.; *n* = 12 donors; one-way ANOVA and Dunnett’s post-hoc test. **c**, Percentages of CCR7 and CD45RO-expressing cells after 4, 7 or 11 days of treatment with increasing doses of ML226 were determined using flow cytometry. Each data point represents one human donor. Mean ± s.d.; *n* = 12 donors; one-way ANOVA and Dunnett’s post-hoc test for each timepoint. **d**, Representative flow cytograms for CCR7, CD45RO, CD45RA, CD95, CD27 and CD28 quantification in Fig. 6d. Source data

Given the pronounced effect ML226 treatment had on fatty acid metabolism, we went on to explore whether metabolic perturbations following ABHD11 inhibition influenced CD8^+^ T cell differentiation. Human CD8^+^ T cells were cultured continuously with ML226 treatment for up to 11 days (Fig. 6a). Short-term treatment with ML226 treatment did not alter T cell activation, as there was no change in the secretion of key cytokines (Extended Data Fig. 9a and Supplementary Fig. 6a) nor the expression of CD25, a key marker of T cell activation (Extended Data Fig. 9b). There were generally no significant changes in T cell exhaustion markers, except at the highest dose of ML226 at day 11 (Supplementary Fig. 6b,c). However, we did observe that ML226 drove a memory T cell phenotype, as after 11 days of treatment, there was an increase in the CCR7^high^CD45RO^high^ central memory T cell population (T_CM_), and a corresponding decrease in the CCR7^low^CD45RO^high^ effector memory T cell population (T_EM_; Fig. 6b,c and Extended Data Fig. 9c).

To confirm the phenotype changes were driven by OGDHc inhibition, we also treated cells with DM-2OG, mimicking impaired 2-OG metabolism by the OGDHc, and found that, similarly to ML226, DM-2OG increased the CCR7^high^CD45RO^high^ population, with a corresponding decrease in the CCR7^low^CD45RO^high^ population (Fig. 6b,c). CD8^+^ T cells treated for 11 days with ML226 or DM-2OG had increased levels of the memory associated markers CCR7, CD45RO and CD95 but not CD27 and CD28 (Fig. 6d and Extended Data Fig. 9d). CD45RA, which is expressed more highly in effector memory T cells re-expressing CD45RA (T_EMRA_) and terminal effector T cells (T_TE_) than T_CM_ and T_EM_, was reduced in ML226 and DM-2OG treated cells (Fig. 6d and Extended Data Fig. 9d).

**Extended Data Fig. 10:**
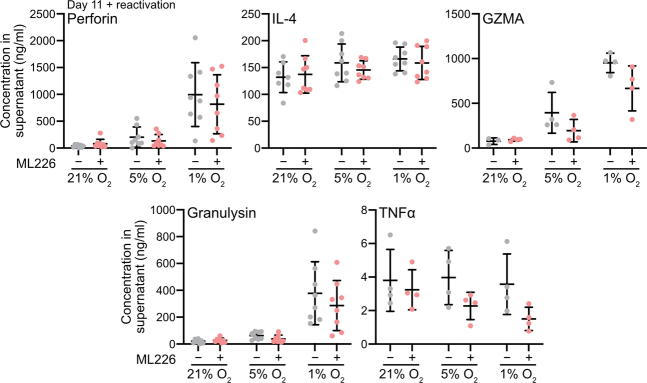
Cytokine release from CD8^+^ T cells treated with ML226. Levels of additional cytokines in cell culture supernatants from Fig. 6e were determined using a commercial multiplexed assay and flow cytometry. Each data point represents one human donor. Mean ± s.d.; *n* = 8 (perforin, IL-4, granulysin), *n* = 4 (GZMA, TNFα) donors; two-way ANOVA and Dunnett’s post-hoc test. Source data

Lastly, as CD8^+^ T cells have to function in low-oxygen environments, we examined the effects of ML226 at varying oxygen tensions. It is not possible to culture the T cells persistently in hypoxia for 11 days, as low oxygen levels can stop T cell proliferation. Therefore, we explored the effect of ABHD11 inhibition on T cell reactivation. CD8^+^ T cells were activated and treated with ML226 for 11 days, as previously described, incubated at 21%, 5% or 1% oxygen for 2 h, and then reactivated with anti-CD3/CD28 Dynabeads. No change in cytokine release (4 h after reactivation) was observed when the CD8^+^ T cells were incubated in 21% or 5% oxygen, but when reactivation was performed at 1% oxygen, levels akin to the tumor microenvironment or sites of infection, ML226-treated CD8^+^ T cells showed reduced secretion of interferon γ (IFNy), granzyme B and Fas ligand (FASL; Fig. 6e). TNF, perforin, granulysin and granzyme A showed a downward trend in 1% oxygen but this was not significant when compared to vehicle treated cells (Extended Data Fig. 10). Together, these findings support a role for ABHD11 in controlling CD8^+^ T cell differentiation, with implications for 2-OG metabolism and oxygen tensions in contributing to the phenotypic differences between T cell states (Fig. 6f).

## Discussion

Lipoylation and glutarylation are both recognized as dynamic post-translational modifications involved in mitochondrial metabolism, cell fate determination and immune regulation, but the enzymatic regulation of these modifications is only beginning to be appreciated^5,9,11,13–16,22,23,29–34^. By distinguishing between lipoyl and protein glutarylation, we were able to uncover a role for ABHD11 in removing Lp_glu_ adducts, thereby maintaining the activity of the OGDHc. Our studies highlight the selective regulation of an acyl-lipoyl conjugate (thioester bond), which is distinct from the role of Sirtuins (SIRTs) in cleaving acyl modifications from lysine residues (amide bonds). SIRT4 has reported lipoamidase activity^29^, and SIRT5 acts as the canonical K_glu_ deglutarylating enzyme^5^. However, both SIRT4 and SIRT5 have more widespread activity in removing other K_acyl_ chains and regulating additional mitochondrial enzymes^35–39^. In contrast, ABHD11 has not yet demonstrated amidase activity and has a predominant effect on Lp_glu_, consistent with a role for nonprotein deglutarylation.

Why the OGDHc-E2 is particularly susceptible to glutaryl–lipoyl adducts is not fully explained. The OGDHc and related OADHc have an intrinsic capacity to remove acyl-lipoyl modifications, as these intermediates are part of the catalytic function of the complexes. However, under conditions of ABHD11 depletion or inhibition, we observe that glutaryl–lipoyl adducts cannot be adequately removed. Potential explanations may relate to the hydrolysis of the acyl chain or result from the same E2 subunit being shared between the OGDHc and OADHc. Glutarate may form more stable adducts compared to succinate, as suggested by the self-hydrolysis observed with the Lp_suc_ peptide compared to the Lp_glu_ form. Alternatively, as Lp_glu_ is an intermediate formed by the OADHc that converts 2-oxoadipate to glutaryl-CoA, persistent glutarylation of the OGDHc-E2 would prevent the formation of glutaryl-CoA by the OADHc, and also render the OGDHc inactive. The recent identification of hybrid ketoacid dehydrogenase complexes between the E1 subunit of OADHc (DHTKD1) and the OGDHc^40^ indicates that more complex crosstalk exists between ketoacid dehydrogenases, and we speculate that these hybrid complexes may be more susceptible to acyl-lipoyl adduct formation. It will therefore be of future interest to understand how these hybrid complexes function and the nature of the lipoate intermediates that form.

In silico modeling of ABHD11 is consistent with the lipoyl domain docking within the catalytic pocket. We previously showed that serine-141 and histidine-296 are required for enzymatic activity^16^, and docking predicts that the thiol ring of lipoate is proximal to these residues. However, CoA does not fit well within the modeled catalytic pocket, consistent with our experimental findings that ABHD11 does not hydrolyze the glutaryl-CoA thioester. Other factors that drive ABHD11 substrate specificity remain to be determined. We detected minimal activity of ABHD11 against succinyl esters, but this analysis was hampered by the spontaneous hydrolysis of the succinyl compounds. It remains plausible that ABHD11 may remove other acyl chains, and it will be of interest to explore this in further work.

The role of ABHD11 in removing Lp_glu_ adducts provides evidence for the regulatory nature of lipoylation. While essential for maintaining key metabolic pathways, this study and other studies demonstrate that lipoyl adducts can serve roles more akin to the signaling occurring via other metabolite post-translational modifications. Lipoyl adducts formed by changes in oxidative or catabolic stress can alter TCA cycle function to allow the cell to adapt to these insults, as demonstrated for transient lipoyl nitrosylation during macrophage activation^15,33^. Lipoyl adducts are also implicated in a recently described cell death pathway, cuproptosis^41^. We now show that Lp_glu_ has a major effect on 2-OG metabolism. However, the downstream consequences of 2-OG accumulation differ according to cell type. In HeLa cancer cells, we find 2-OG accumulation drives 2-HG-mediated inhibition of several 2-OGDDs. Indeed, this association between 2-OG, 2-HG and 2-OGDD activity is well substantiated^16,22,24,42–44^. However, not all cells will respond similarly to 2-OG accumulation, and such differential effects are to be expected. For example, in mouse embryonic stem cells, excess 2-OG maintains pluripotency dependent on the 2-OG/succinate ratio by increasing TET and KDM activity^45^. In this stem cell context, 2-OG conversion to 2-HG does not seem to be phenotypically important. Here, we show that impaired OGDHc activity (via ABHD11 inhibition) in human CD8^+^ lymphocytes has distinct effects from those observed in cancer cells, with the perturbed TCA cycle driving synthesis of unsaturated fatty acids, rather than the accumulation of 2-HG that inhibits 2-OGDDs. The full details of how this metabolic rewiring occurs will require future work, but it is likely related to shunting 2-OG via reductive metabolism and acetyl-CoA to drive fatty acid synthesis. Interestingly, fatty acid esterification into triglycerides has previously been shown to support memory CD8^+^ T cell survival^46^, and these prior observations are in line with our findings with ABHD11 inhibition.

High levels of ABHD11 expression in immune cell populations^25^ highlight ABHD11’s likely role in immunity. In CD8^+^ T lymphocytes, our findings indicate a role for ABHD11 and 2-OG metabolism in differentiation, with ABHD11 inhibition increasing the pool of CD8^+^ T_CM_ cells—a T cell population that displays stem cell-like properties^47^. We also observed that in 1% oxygen, ABHD11 activity altered the secretion of cytokines involved in CD8^+^ T cell cytotoxicity. Further work is needed to understand the full extent of manipulating ABHD11 on CD8^+^ T cell biology. In particular, how ABHD11 influences the function and activity of other immune effector cells where it is highly expressed remains to be seen. However, our mechanistic understanding of how ABHD11 functions as a nonprotein deglutarylase provides the necessary foundation for exploring the potential utility of targeting ABHD11 in different immune cell populations.

## Methods

### Cell lines and reagents

HEK293T and HeLa cells were maintained in high-glucose DMEM (Sigma-Aldrich, D6429) containing 10% fetal calf serum (Sigma-Aldrich, P4333), 100 U ml^−1^ penicillin and 100 µg ml^−1^ streptomycin at 37 °C and 5% CO_2_. HEK293T cells were purchased from American Type Culture Collection and HeLa cells were a gift from the Lehner laboratory (University of Cambridge). All cell lines were authenticated by short tandem repeat profiling (Eurofins Genomics) and routinely tested for mycoplasma. Clonal HeLa cell lines expressing Cas9 (with a hygromycin selection cassette) or an HRE-GFP^ODD^ reporter construct were previously generated by the laboratory^22^. The reagents used in this study are listed in Supplementary Table 1.

### CD8^+^ T cell isolation and culture

Peripheral blood mononuclear cells (PBMCs) were isolated from anonymized healthy blood donors obtained from the National Health Service (NHS) Blood and Transplant (Addenbrooke’s Hospital) or the Blood Donor Center at Boston Children’s Hospital. Ethical approval was obtained from the East of England-Cambridge Central Research Ethics Committee (06/Q0108/281), and consent was obtained from all participants. PBMCs were obtained via density-gradient separation and were either used fresh or cryopreserved in FBS supplemented with 10% DMSO. Total CD8^+^ T cells were purified from PBMCs by magnetic-activated cell sorting using CD8 microbeads (Miltenyi, 130-045-201). Immediately after isolation, CD8^+^ T cells were activated with anti-human CD3/CD28 Dynabeads (Thermo Fisher Scientific, 11131D) at a 1:1 cell:bead ratio and cultured in complete RPMI 1640 supplemented with 10% FBS, 100 U ml^−1^ penicillin, 100 µg ml^−1^ streptomycin and 30 U ml^−1^ IL-2 (Sigma-Aldrich, 11147528001). For experiments involving oxygen control, purified CD8^+^ T cells were immediately acclimatized to the required oxygen tension (21%, 5% or 1% O_2_) for 2 h in complete RPMI 1640 supplemented with 10% FBS, 100 U ml^−1^ penicillin and 100 µg ml^−1^ streptomycin, before activation with anti-CD3/CD28 Dynabeads and 30 U ml^−1^ IL-2.

For experiments, CD8^+^ T cells were seeded at a density of 5 × 10^5^ cells per ml medium. Treatments with ML226 and DM-2OG (or 0.1% vol/vol DMSO vehicle) were performed at anti-CD3/CD28 activation unless otherwise indicated. Low-oxygen incubations (5% or 1% O_2_) were carried out in a Ruskinn SCI-tive workstation. Cell number was determined using a CellDrop Automated Cell Counter (DeNovix) or the Countess 3 Automated Cell Counter (Thermo Fisher Scientific).

The Immunological Proteome Resource (http://immpres.co.uk/) was used to examine ABHD11 protein expression in immune cell subsets. This is an open proteomics resource for the immunoproteome^25^. Mean and standard deviation values were obtained directly from the database.

### Plasmids

CRISPR–Cas9 sgRNA vectors targeting β2M and ABHD11 were previously generated by the laboratory^16^. Sequences targeting GCDH (RefSeq ID: 2639) were identified using VBC-Score, and the top three hits were cloned into the pKLV-U6sgRNA pGK Puro-2A-BFP vector (Addgene, 50946) using BbsI cloning. Plasmid identity was verified using Sanger sequencing, and the most efficient sgRNA (as determined by anti-GCDH immunoblotting) was used in subsequent experiments (sgRNA sequences are listed in Supplementary Table 1).

### Lentivirus preparation and transduction

To prepare lentivirus particles, 4 × 10^5^ HEK293T cells were seeded into six-well plates and transfected the next day with 0.66 µg expression plasmid, 0.44 µg pCMV-dR8.91 (gag/pol) and 0.88 µg pMD.G vesicular stomatitis virus G glycoprotein using 8 µl FuGENE HD transfection reagent (Promega). After 48 h, viral supernatants were collected, filtered (0.45 µm) and stored at −80 °C in single-use aliquots.

For lentiviral transduction, 0.2 × 10^6^ HeLa–Cas9 cells were seeded into 24-well plates containing 500 µl viral supernatant. After 24 h, cells were expanded into six-well dishes and selected in 1 µg ml^−1^ puromycin for 3 days, then further expanded in nonselective medium. Experiments were performed using mixed populations 11 days post-transduction.

### Immunoblotting

To profile protein lipoylation and lysine glutarylation, 0.5 × 10^6^ HeLa cells or 1 × 10^6^ T cells were lysed in SDS loading buffer (2% (wt/vol) SDS, 50 mM Tris (pH 7.4), 150 mM NaCl, 1 mM dithiothreitol, 10% (vol/vol) glycerol and 0.2% (vol/vol) benzonase nuclease (Sigma-Aldrich, E1014)) and heated at 90 °C for 5 min.

Samples were separated on SDS–PAGE gels, transferred to PVDF membranes and blocked in 2% fatty acid-free BSA and 5% skim milk powder in PBS containing 0.2% Tween-20 (PBST) for lipoate detection, or 5% skim milk powder in PBST for glutaryl-lysine detection. Blots were incubated in the appropriate primary antibodies (Supplementary Table 1) followed by HRP-conjugated secondary antibody in blocking buffer. Immunoblots were developed on film using HRP-conjugated secondary antibody and Pierce ECL Western Blotting Substrate (Thermo Fisher Scientific, 32106). To quantify total PDHc-E2 and OGDHc-E2, lipoate blots were stripped using Restore Western Blot Stripping Buffer (Thermo Fisher Scientific, 21059), reblocked in 5% skim milk in PBST and re-immunoblotted using appropriate primary antibodies. The signal was quantified using Image Studio Lite v5.5 (LI-COR Biosciences) or ImageJ. Other proteins were analyzed by re-immunoblotting with appropriate primary antibodies. Modification levels were normalized to total levels of the relevant protein, then adjusted relative to the control condition.

To profile histone methylation marks, immunoblotting was performed as described above using 12% SDS–PAGE gels and blocking in 5% skim milk in PBST. Membranes were stripped and reblocked between each primary antibody incubation. Methylation marks were normalized to total H3 levels, then adjusted relative to the control condition.

### OGDHc assay

OGDHc activity in whole cell lysates was measured using the α-Ketoglutarate Dehydrogenase Activity Assay Kit (Colorimetric; Abcam, ab185440). Briefly, 1 × 10^6^ cells were lysed in 100 µl assay buffer, freeze thawed on dry ice twice and homogenized by passing ten times through a 27-gauge needle. After centrifuging at 10,000*g* and 4 °C for 5 min, 10 µl of supernatant was used for the assay in technical duplicates. Absorbance was measured at 450 nm and 37 °C in a CLARIOstar Plus Microplate (BMG LABTECH) using kinetic mode with measurements every 1 min for 45 min. Background absorbance was quantified in samples without substrate addition. Lysates from untreated cells were assayed with or without 10 mM KMV in the assay buffer as a positive control for OGDHc inhibition. For cells treated with ML226, a final concentration of 2.5 µM was maintained throughout the assay to prevent washout. To quantify specific OGDHc activity, background absorbance was subtracted for each sample, and the slope was derived for the linear phase of the signal curve.

### Quantitative PCR

To analyze mRNA expression, total RNA was collected from cells using the PureLink RNA Mini Kit (Thermo Fisher Scientific), and 1 µg was reverse-transcribed using the ProtoScript II First Strand cDNA Synthesis Kit with oligo(dT) primers and murine RNase inhibitor (New England Biolabs). Quantitative PCR was performed in technical triplicate using 20 ng cDNA, 1× Power SYBR Green PCR Master Mix (Thermo Fisher Scientific) and 0.5 µM forward and reverse primers (Supplementary Table 1), using an ABI 7900HT Real-Time PCR System (Applied Biosystems). Target gene expression was calculated using QuantStudio v1.3 (Applied Biosystems), normalized to the *ACTB* housekeeping gene using the ΔΔC_T_ method, and adjusted relative to the control condition.

### Flow cytometry

To profile reporter stability in HeLa HRE-GFP^ODD^ cells, live cells were washed twice with cold PBS and immediately analyzed using an LSR II flow cytometer (BD Biosciences). GFP signal was detected by a 488 nm laser with 530/30 filter. Data analysis was performed using FlowJo v10.9 (BD Biosciences).

To quantify protein levels in CD8^+^ T cells, single-cell suspensions were stained using the LIVE/DEAD Fixable Near-IR Dead Cell Stain Kit (Thermo Fisher Scientific, 10119), followed by surface and intracellular staining with fluorochrome-labeled antibodies (Supplementary Table 1). Staining of cytoplasmic and nuclear antigens was performed using the Cytofix/Cytoperm Fixation/Permeabilization Kit (BD Biosciences, 554714) and Transcription Factor Buffer Set (BD Biosciences, 562725), respectively. Data were collected using an Aurora flow cytometer (Cytek Biosciences), and a representative gating strategy is included (Supplementary Fig. 7).

For mitochondria analysis, cells were washed twice in RPMI media (no FBS) loaded with 10 nmol ml^−1^ Mitotracker Green (Thermo Fisher Scientific), 25 nmol ml^−1^ TMRM (Thermo Fisher Scientific), or 5 mmol ml^−1^ MitoSox Red for 20 min at 37 °C in RPMI (no FBS). Cells were then washed twice and resuspended in PBS with 5% FBS before analysis. Cells were treated with 1.5 µM rotenone for 30 min at 37 °C before MitoSox staining. Data were collected using an Aurora flow cytometer (Cytek Biosciences), and the gating strategy is included (Supplementary Fig. 7). Data analyses were performed using FlowJo v10.9 and FlowJo v10.10 (BD Biosciences).

### DNA hydroxymethylation analysis

5hmC levels were quantified as previously described^16^. Briefly, genomic DNA was collected from 1 × 10^6^ cells using the Puregene Cell Kit (Qiagen). DNA was diluted to 100 ng µl^−1^ in 0.4 M NaOH and 10 mM EDTA (pH 8.0) and denatured by heating at 100 °C for 10 min. Serially diluted DNA was spotted in 3× 2 µl droplets onto a Hybond NX nylon membrane (Amersham) and allowed to dry. Membranes were incubated in 2× SSC buffer (Sigma-Aldrich, J60839.K2), air dried and UV-crosslinked at 120,000 µJ cm^−^^2^ for 150 s using a UV Stratalinker 2400 (Stratagene). For immunoblotting, membranes were blocked in 5% skim milk powder and 1% BSA in PBST for 1 h and incubated in anti-5-hydroxymethylcytosine antibody. Signal was developed on film using HRP-conjugated secondary antibody and SuperSignal West Dura Extended Duration Substrate (Thermo Fisher Scientific, 34075). For total DNA staining, membranes were incubated in 0.1% (wt/vol) methylene blue solution containing 0.5 M sodium acetate (pH 5.2) overnight at room temperature. After 3× 5 min washes with distilled water, membranes were air dried and scanned. Signal from 5hmC immunoblots was quantified using Image Studio Lite v5.5 (LI-COR Biosciences) and normalized to methylene blue signal, then adjusted relative to the control condition. Within each biological replicate, adjusted data were averaged among the serial dilutions of genomic DNA.

### Immunoprecipitation

To enrich PDHc-E2 or OGDHc-E2 for proteomics analysis, 5 × 10^6^ cells were lysed in 1.5 ml IP buffer (50 mM HEPES (pH 7.0), 150 mM NaCl, 1% (vol/vol) IGEPAL CA-630, 1 mM phenylmethylsulfonyl fluoride, 10 mM Tris(2-carboxyethyl)phosphine hydrochloride, 100 mM nicotinamide, 1× Roche Complete Protease Inhibitor Cocktail) and rotated at 4 °C for 30 min. Lysates were pelleted at 16,900*g* and 4 °C for 10 min, and 5% of the supernatant volume was collected as an input control. The remaining supernatant was mixed with 10 mM N-ethylmaleimide, rotated at 4 °C for 1 h and precleared with Pierce Protein G magnetic beads (Thermo Fisher Scientific, 88848) by rotating at 4 °C for 1 h. The supernatant was then mixed with 100 µl Protein G magnetic beads bound to 10 µl anti-PDHc-E2 or anti-OGDHc-E2 antibody and immunoprecipitated at 4 °C for 16 h. Beads were washed four times with IP wash buffer (50 mM Tris–HCl (pH 7.4), 150 mM NaCl, 0.1% (vol/vol) IGEPAL CA-630), then thrice with IP wash buffer lacking IGEPAL CA-630. Proteins were eluted by heating in SDS loading buffer at 90 °C for 5 min, and lipoylation phenotypes were verified by immunoblotting the eluate alongside the input control. The above procedure was scaled down for in vitro assays requiring smaller-scale immunoprecipitation from 10 × 10^6^ cells.

### Post-translational modification analysis

For MS analysis of PDHc-E2 or OGDHc-E2 modifications, immunoprecipitation eluates were separated on a pre-cast 4% to 12% NuPAGE gel (Thermo Fisher Scientific, NP0321BOX) and protein bands were stained using SimplyBlue SafeStain solution (Thermo Fisher Scientific, LC6060). After several washes with distilled water, bands corresponding to PDHc-E2 (~75 kDa) or OGDHc-E2 (~55 kDa) were excised using a sterile scalpel and transferred to Protein LoBind tubes (Eppendorf). Samples were reduced, alkylated and digested in-gel with either trypsin or AspN, and digested peptides were eluted with MeCN/water and two 5% FA washes. Peptides were pooled in 0.5 ml Protein LoBind tubes, dried almost to completion and resuspended in 20 µl solvent (3% MeCN, 0.1% TFA) with 7 µl analyzed by LC–MS/MS.

MS data (LC–MS/MS) were acquired as described previously^9^. An Orbitrap Fusion Lumos coupled to an Ultimate 3000 RSLC nano UHPLC equipped with a 100 µm inner diameter × 2 cm Acclaim PepMap Precolumn (Thermo Fisher Scientific) and a 75 µm inner diameter × 50 cm, 2 µm particle Acclaim PepMap RSLC analytical column was used. Loading solvent was 0.1% FA with analytical solvents A: 0.1% FA and B: 80% MeCN + 0.1% FA. Samples were loaded at 5 µl min^−1^ loading solvent for 5 min before beginning the analytical gradient. The analytical gradient was 3–40% solvent B over 42 min, rising to 95% solvent B by 45 min, followed by a 4 min wash at 95% solvent B and equilibration at 3% solvent B for 10 min. Columns were held at 40 °C. Data was acquired in a DDA fashion with the following settings: MS1—380–1500 Th, 120,000 resolution, 4 × 10^5^ AGC target, 50 ms maximum injection time. MS2—quadrupole isolation at an isolation width of *m*/*z* 1.6, HCD fragmentation (NCE 30) with fragment ions scanning in the Orbitrap from *m*/*z* 110, 5 × 10^4^ AGC target, 100 ms maximum injection time. Dynamic exclusion was set to ±10 ppm for 60 s. MS2 fragmentation was triggered on precursors with 5 × 10^4^ counts and above.

Raw files were processed using PEAKS 11 and PEAKS Studio (v8.0, Bioinformatics Solutions). Variable modifications include oxidation, carbamidomethylation, lipoylation, 1× and 2× NEM lipoylation, glutaryl lipoate, NEM glutaryl lipoate and 309 built-in modifications at PEAKS PTM stage. The area under the curve (AOC) for each peptide was extracted from the PEAKS peptide list, with AOCs being calculated by PEAKS (all raw data is included in Supplementary Data 1).

### ABHD11 in silico modeling

#### Homology model building

The 2.03 Å x-ray structure of marine esterase (α/β-hydrolase fold; Research Collaboratory for Structural Bioinformatics Protein Data Bank ID: 7c4d.1, www.pdb.org)^21^ was downloaded and homology similarity of ABHD11 assessed in Swiss-model software (proMod3 v3.3.0, www.swiss.org), in the server platform where the ABHD11 model was initially built. To refine the new output (model ABHD11), building locally, a Coot v0.9.8.8 (EL, CCP4i)^48^ command line and interactive molecular graphics interface software were used. The 7c4d.1 (reference) and ABHD11 coordinates, similarity and active site pocket residues were topologically overlaid onto corresponding positions in the reference coordinate and visualized in Coot^48^. The ABHD11 coordinate (ABHD11.mmcif) was then manually edited and superimposed onto the reference electron density map (.mtz file of 7c4d.1). The structure, active site residues, outliers of loops, side chains, or unreliable atomic coordinates were closely monitored. Particular attention was paid to a poorly resolved area of 50 amino acids at the N terminus, which was assigned to zero occupancy in the newly generated model.

#### Preparing the ligand coordinate

The (xx(lipoyl)lysine) ligand was sketched manually using molview.org for visualization, and the ligand atoms were then converted into a crystallographic information file (.cif), atomic coordinate format. The hydrogen bond distances were monitored/corrected in SHELX (CCP4i)^49^, and the output file was then read in Coot.

#### Ligand docking in the active site of ABHD11

Both the (xx(lipoly)lysine).cif and ABHD11.cif coordinates were submitted to the HADDOCK2.4 server^50^, where molecular docking for this experiment was performed based on 3D structures and ligand input. An output of 10 binding models from the system was released, based on different configurations and ligand coordinate interactions. The top-scoring complex coordinate output file was selected based on the atomic interaction, atomic distance/clashing, binding interface with ligand (xx(lipoyl)lysine) and surface charges in the active site pocket following display of each coordinate in Coot.

All 3D model figures, surface rendering and active site occupancy representations of the complex were created with PyMOL v3.0.4 and Pymol-plungin/command line script.

### ABHD11 purification

To purify ABHD11-Flag protein, 1 × 10^8^ HeLa cells expressing ABHD11-Flag were lysed in 1 ml lysis buffer (50 mM Tris–HCl pH 7.4, 150 mM NaCl, 1% Triton X-100, 1× Roche complete protease inhibitor cocktail) and rotated at 4 °C for 1 h. Lysates were pelleted at 16,900*g* and 4 °C for 10 min, and 5% of the supernatant volume was collected as an input control. Remaining supernatants were then precleared with CL4B resin and rotated at 4 °C for 1 h. CL4B resins were pelleted at 16,900*g* and 4 °C for 1 min, and the supernatants were then incubated with 200 µl M2 magnetic beads (Sigma-Aldrich, M8823) and immunoprecipitated at 4 °C for 16 h. Beads were then washed four times with wash buffer (50 mM Tris–HCl pH 7.4, 150 mM NaCl, 0.1% Triton X-100) and then twice with wash buffer lacking Triton X-100. ABHD11-Flag protein was then eluted in 600 µl 100 µg ml^−1^ 3X Flag peptide and rotated at 4 °C for 1 h. Elution was then repeated. Concentration and purity of ABHD11-Flag protein were then verified in eluates using Coomassie Simply Safe stain. Dependent on protein purity, pooled eluates were either dialyzed immediately or further purified using anion exchange chromatography.

Purified ABHD11-Flag was dialyzed (SnakeSkin dialysis tubing, 10 MWCO; Thermo Fisher Scientific) against one litre of TBS (50 mM Tris–HCl pH 7.4, 150 mM NaCl) with a magnetic stirrer at 4 °C for 16 h, followed by a second dialysis with a magnetic stirrer at 4 °C for 2 h. Purity of ABHD11-Flag protein was then verified in the dialyzed sample using Coomassie Simply Safe stain and the concentration compared with BSA standards. Dialyzed ABHD11-Flag was then concentrated, if required, using a centrifugal filter column (Vivaspin 2, 10,000 MWCO) at 700*g* and 4 °C for 3 min. After adding 10% glycerol, concentration and purity of ABHD11-Flag protein were verified by Coomassie Simply Safe stain and BSA standards. Aliquots were stored at −80 °C.

### Thioesterase assays

#### ABHD11 reconstitution assay

A total of 1 × 10^6^ HeLa cells treated with or without 2.5 µM ML226 for 6 h were lysed and immunoprecipitated as described. The OGDHc-E2 bound resins were washed in IP wash buffer (without IGEPAL CA-630) and resuspended in 70 µl TBS (50 mM Tris pH 7.4, 150 mM NaCl). A 10 µl aliquot of the resins was retained to verify the immunoprecipitation by immunoblot. For the assay, 20 µl of resin was aliquoted into three 1.5-ml Eppendorf tubes for each condition, with 20 µl of ABHD11-Flag or vehicle control added. Samples were incubated for 15 or 30 min at 37 °C on a shaking heat block. At each endpoint samples were placed on ice, centrifuged at 700*g* 4 °C for 30 s and 4 ml 6× SDS loading buffer was added. Samples were then heated at 90 °C for 5 min, centrifuged at 700*g* for 30 s, placed on a magnet for 1 min, and supernatants were collected for SDS–PAGE analysis. Lipoate and OGDHc-E2 levels were measured by immunoblot. The above procedure was also performed for the CoA/NAD^+^ supplementation assay by adding 3 mM CoA and 0.5 mM NAD^+^ in place of ABHD11-Flag. The major reagents used in this study are listed in Supplementary Table 1.

#### DTNB assay

Ten milligrams of unmodified OGDHc-E2, lipoyl OGDHc-E2, diglutaryl lipoyl and disuccinyl lipoyl OGDHc-E2 peptides were generated by Peptide Synthetics to >90% purity and reconstituted to 6.56 mM in DMSO. Glutaryl-CoA and succinyl-CoA were reconstituted in DMSO to 6.56 mM. Peptides, glutaryl-CoA or succinyl-CoA were subsequently diluted to 2.5 mM in assay buffer (50 mM Tris–HCl pH 7.4, 100 mM NaCl). DTNB (Sigma-Aldrich) was dissolved in 10× PBS (pH 7.4) to make a 4 mg ml^−1^ (10 mM) solution and subsequently diluted in assay buffer to a final concentration of 0.2 mM. A total of 100 µM of the peptides, glutaryl-CoA or succinyl-CoA were added to 100 nM ABHD11-Flag (50 mM Tris–HCl pH 7.4, 100 mM NaCl) and 0.2 mM DTNB reagent in a 96-well plate on ice. The 2.5 µM ML226 was added at the start of the reaction to experiments measuring ABHD11 inhibition. Absorbance was measured at 412 nm in a plate reader (CLARIOstar Plus Microplate, BMG LabTech) using kinetic mode with measurements every 45 s for 45 min at 37 °C. Michaelis–Menten kinetics were calculated by first obtaining the concentration of TNB formed under the reactions (*c* = *A*/*bE*), where *c* is the concentration, *A* is absorbance, *b* is the path length (1 cm) and *E* is the molar absorptivity (molar extinction coefficient of TNB is 14,150 M^−1^ cm^−1^ (ref. ^51^). The velocity (V, mM min^−1^) was calculated from the slope of the kinetic assays. The Michaelis–Menten model was plotted in GraphPad Prism v10.1.1, and the *K*_*M*_, *k*_cat_ and *k*_cat_/*K*_*M*_ calculated. The major reagents used in this study are listed in Supplementary Table 1.

### LC–MS metabolomic analyses

#### In vitro assay for succinate or glutarate release

Experiments were performed as for the DTNB assay, but without the addition of 0.2 mM DTNB reagent. A total of 10 mg of Lp_glu_ OGDHc-E2, Lp_succ_ OGDHc-E2 or K_glu_ OGDHc-E2 peptides and succinyl-CoA were reconstituted to 6.56 mM in DMSO. These substrates were subsequently diluted to 2.5 mM in assay buffer (50 mM Tris–HCl pH 7.4, 100 mM NaCl). A total of 100 nM ABHD11-Flag was added to 100 µM of the substrates (OGDHc-E2 peptides or succinyl-CoA) in assay buffer (50 mM Tris–HCl pH 7.4, 100 mM NaCl) in 1.5 ml Eppendorf tubes for each condition on ice. The 2.5 µM ML226 was added at the start of the reaction to experiments measuring ABHD11 inhibition. Samples were incubated for 45 min at 37 °C on a shaking heat block, centrifuged at 700*g* 4 °C for 30 s, and then placed on ice for immediate LC–MS analysis.

A Vanquish Horizon UHPLC system coupled to a Q Exactive Plus MS (both Thermo Fisher Scientific) was used to detect glutarate or succinate. The sample was prepared as follows: samples (20 µl) were quenched with 200 µl 4:1 methanol:water in Eppendorf tubes, vortexed, and centrifuged for 2 min at 21,300*g*. Supernatants (200 µl per sample) were then transferred to fresh Eppendorf tubes, dried in a SpeedVac vacuum centrifuge (Savant; Thermo Fisher Scientific, SC210A) and reconstituted in 20 µl 10 mM ammonium acetate in MilliQ water containing a mix of the following internal standards: universal ^15^N^13^C amino acid mix, succinate ^13^C_4_, AMP ^15^N_5_^13^C_10_, ATP ^15^N_5_^13^C_10_, putrescine D8 and dopamine D4. Samples were vortexed and, after a brief centrifugation, transferred to SureSTART 0.3 ml vials (Thermo Fisher Scientific) and fitted with SureSTART 9 mm screw caps (Thermo Fisher Scientific). A standard of glutarate (Merck) was used to confirm the retention time (RT) of glutarate. The succinate ^13^C_4_ internal standard confirmed the RT of succinate. Chromatography was performed by injecting samples (3.5 µl injection volume) onto an ACE EXCEL 2 C18-PFP 150 × 2.1 mm column (Avantor, EXL-1010-1502U), which was maintained at 30 °C in the column compartment. Mobile phase A was water (MilliQ) containing 0.1% formic acid (Thermo Fisher Scientific), whereas mobile phase B was acetonitrile (Thermo Fisher Scientific) containing 0.1% formic acid. The gradient used was as follows (at a flow rate of 0.5 ml min^−1^): 0% solvent B for 1.6 min, followed by an increase to 30% solvent B over 2.4 min, a further increase to 90% solvent B over 0.5 min, a hold at 90% solvent B for 0.5 min, a return to 0% solvent B over 0.1 min and equilibration at 0% solvent B for 1.4 min, for a total run time of 6.5 min per sample. The needle wash used was 1:1 acetonitrile:water. A full MS scan was performed at 55–825 *m*/*z*, with a resolution of 140,000 ppm, in negative ion mode. The auxiliary gas heater and capillary temperatures were 450 °C and 275 °C, respectively, with an ion spray voltage of 2.5 kV. The sheath, auxiliary and sweep gas flow rates were 55, 15 and 3 a.u., respectively, and the S-lens radio frequency was 50%. Targeted data analysis was performed using Thermo Scientific Xcalibur (version 4.1.31.9). Area ratios of the peak areas of glutarate (*m*/*z* 131.0350 [M–H]) to ^13^C_9_^15^N phenylalanine internal standard (*m*/*z* 174.0989 [M–H]), or succinate (*m*/*z* 117.0193 [M–H]) to ^13^C_4_ succinate internal standard (*m*/*z* 121.0328 [M–H]) were calculated. The targeted analysis of succinate and glutarate was always paired, as both metabolites could be detected by their distinct RT.

#### LC–MS metabolite quantification in HeLa cells

A total of 1 × 10^6^ treated HeLa cells or 1 × 10^6^ Cas9-expressing HeLa cells were collected, washed with cold PBS and metabolic activity quenched by freezing samples in dry ice and ethanol, and stored at −80 °C. Before extraction, 10 µl of the internal standard solution (d_4_-succinic acid, ^13^C_4_-glutaric acid and ^13^C_5_-2-hydroxyglutarate; 100 μg ml^−1^ each) was added to all samples. Metabolites were subsequently extracted by addition of 600 μl ice-cold LC–MS grade methanol to the cell pellets, followed by 30 min sonication (sweep mode). During sonication, ice was added to the ultrasound bath to maintain the temperature below 20 °C. Following sonication, samples were centrifuged (12000*g*, 15 min, 10 °C) and 200 µl of the supernatant was transferred to LC–MS vials for the subsequent analysis.

Targeted analyses were performed on a Waters Xevo TQ-Sµ Triple Quadrupole MS (Waters). An Atlantis Premier BEH Z-HILIC FIT Column (2.1 mm × 100 mm, 1.7 μm), equipped with an integrated guard column (Vanguard FIT), was used for the separation, with aqueous mobile phase consisting of 10 mM ammonium acetate (pH 9.1, adjusted with NH_4_OH) in double-deionized water and organic mobile phase consisting of 0.1% formic acid in acetonitrile. The column oven was set at 30 °C, and 1 µl was injected for each sample. The flow rate was set at 0.45 ml min^−1^ (the chromatographic gradient used is shown in Supplementary Table 2).

MS analyses were performed using electrospray ionization (ESI) operating in the negative mode ionization using the following parameters: capillary potential of 1.0 kV, source temperature of 150 °C and desolvation temperature of 600 °C. Compound identification was based on RT and at least one single reaction monitoring (SRM) transition that matched the standards used to build the calibration curve. The RT and the quantifier (Quant) and qualifier (Qual) SRM transitions for the reported compounds were as follows: 2-hydroxyglutarate (RT = 5.5 min, Quant = 147 > 57, Qual = 147 > 129), 2-oxoglutarate (RT = 5.4 min, Quant = 145 > 57), glutarate (RT = 5.5 min, Quant = 131 > 69, Qual = 131 > 113), pyruvate (RT = 1.7 min, Quant = 87 > 43, Qual = 87 > 32) and succinate (RT = 5.4 min, Quant = 117 > 73, Qual = 117 > 99). Raw data is included in the Source data.

#### LC–MS metabolite quantification in CD8^+^ T cells

For T cell metabolite profiling, cells were washed once with room temperature 1× PBS and once with room temperature NaCl (150 mM) and immediately quenched in liquid nitrogen. Metabolites were extracted using a methanol/water/chloroform (1:1:1) solution. The water-methanol phase containing polar metabolites was dried down and reconstituted with water/acetonitrile (1:1), with the volume normalized to cell number. The chloroform phase containing lipids was dried down and reconstituted with methanol/chloroform (9:1), with the volume normalized to cell number. Polar metabolites were separated using a Vanquish U-HPLC system (Thermo Fisher Scientific) coupled to a Q-Exactive HF-X mass spectrometer equipped with an HESI probe (Thermo Fisher Scientific), operating in negative ion mode. The LC system used a hydrophilic interaction liquid chromatography 150 × 2.1 mm iHILIC1-(P) Classic polymeric column (The Nest Group), along with a 2.1 × 20 mm iHILIC1-(P) Classic Guard column (The Nest Group). Buffer A consisted of 20 mM ammonium carbonate in water with 0.1% ammonium hydroxide, while buffer B was 100% acetonitrile. The flow rate was maintained at 0.15 ml min^−1^ with the following linear gradient: 0–23 min from 95% buffer B to 5% buffer B, 23–25 min hold at 5% buffer B, 25–25.5 min transition to 95% buffer B at 0.20 ml min^−1^, 25.5–32.5 min hold at 95% buffer B and 32.5–33 min at 95% buffer B with a 0.15 ml min^−1^ flow rate. The column was maintained at 25 °C, and the autosampler at 4 °C. MS acquisition was conducted in full scan mode over a mass range of 70–1,000 *m*/*z*, with a resolution of 60,000 at 200 *m*/*z*, an AGC target of 1 × 10^6^ and a maximum injection time of 100 ms. MS data processing, including targeted feature extraction and quantification, was performed using Skyline v24.1. Metabolites were identified based on the instrument’s resolution settings and RT, referenced against an in-house library of chemical standards. Peak area integration and metabolite identification were conducted using accurate mass and RT, curated with in-house standard library compounds. Statistical analysis of metabolomics data was performed using RStudio and GraphPad Prism v9.5.1. Nonpolar metabolites were separated using a Kinetex EVO C18 column (2.6 µm, 150 mm × 2.0 mm inner diameter; Phenomenex) coupled to an Agilent 6546 LC/Q-TOF system with a Dual AJS ESI source operated in positive ion mode. One ESI source was used for the sample, and the other was used for online calibration. Online calibration mix contained TFANH4, purine and hexakis. Buffer A (95% H_2_O, 5% methanol, 5 mM ammonium acetate—pH adjusted to 5.0 with acetic acid) and buffer B (95% isopropanol, 5% methanol) were used. The column was maintained at 40 °C, and the autosampler at 4 °C. MS acquisition was conducted in full scan mode over a mass range of 70–1,700 *m*/*z*. The sample was scanned with an acquisition rate of two spectra per second. For the separation, the chromatographic gradient used is shown in Supplementary Table 2.

MS data targeted feature extraction and quantification were performed on Agilent MassHunter Profinder 10.0. Metabolites were identified using exact mass with a 15 ppm tolerance and RT with reference to an in-house library of chemical standards. Peak area integration and metabolite identification were performed using accurate mass and RT curated with in-house standard library compounds. RStudio 2024.12.0 Build 467 and GraphPad Prism v9.5.1 were used for metabolomics statistical data. Differential metabolite abundance was assessed using linear models fitted separately for each metabolite (lm in R), with group as the predictor. *P*-values for the group effect were extracted from the model coefficients, and significance was determined using false discovery rate correction (Benjamini–Hochberg method), with adjusted *P*-value < 0.05 and absolute log_2_(fold change) > log_2_(1.25).

### Cytokine release assays

To quantify CD8^+^ T cell cytokine release, 200 µl of cell culture supernatant was collected 4–24 h postactivation, centrifuged at 2,000*g* for 2 min to remove remaining cells and stored at −20 °C until analysis. Samples were thawed at room temperature and cytokine concentrations (IL-2, IL-4, IL-6, IL-10, IL-17A, IFNγ, TNF, soluble Fas, soluble FasL, granzyme A, granzyme B, perforin and granulysin) was quantified using the LEGENDplex Human CD8/NK V02 Panel (BioLegend, 741187), according to the manufacturer’s instructions. Data were collected using an Aurora flow cytometer (Cytek Biosciences) and analyzed using the LEGENDplex Data Analysis Software Suite (Qognit). A minimum of two technical replicates were analyzed per biological replicate.

### Metabolic flux analyses

To measure oxygen consumption and extracellular acidification, a Seahorse XFe96 Extracellular Flux Analyzer (Agilent) was used. Here 1.5 × 10^5^ CD8^+^ T cells were plated in Seahorse XF cell culture plates coated with poly-d-lysine (Sigma-Aldrich, P7280) and pre-incubated in appropriate medium at 37 °C and in the absence of CO_2_ for 1 h prior to treatments. Three basal measurements were made at 3 min intervals, followed by three measurements per treatment. A minimum of four technical replicates were analyzed per biological replicate.

For mitochondrial stress tests, the pre-incubation medium consisted of Seahorse XF RPMI medium (pH 7.4; Agilent, 10376-100) supplemented with 2 mM glutamine (Thermo Fisher Scientific, 25030081) and 10 mM glucose (Thermo Fisher Scientific, A2494001). Treatments comprised 1 μM oligomycin (Sigma-Aldrich, 75351), 1.5 μM FCCP (Sigma-Aldrich, C2920) and 100 nM rotenone (Sigma-Aldrich, R8875) plus 1 μM antimycin A (Sigma-Aldrich, A8674). For glycolysis stress tests, the pre-incubation medium consisted of Seahorse XF RPMI medium (pH 7.4) supplemented with 2 mM glutamine. Treatments comprised 10 mM glucose, 1 μM oligomycin and 50 mM 2-deoxy-d-glucose (BioVision, B1048-100).

### Bulk RNA-sequencing

Human CD8^+^ T cells were isolated from PBMCs and activated and cultured with or without ML226 for 11 days as previously described. On day 11, cells were washed twice with ice-cold PBS and cell pellets were snap frozen. Cell pellets were later thawed and lysed with RLT Plus lysis buffer, and total RNA was extracted with the Qiagen RNeasy kit according to the manufacturer’s instructions (Qiagen, 74134). RNA was eluted in RNase-free water and sample concentrations and RNA quality were determined by nanodrop and bioanalyzer (Agilent 5400). Fast RNA-seq Lib Prep Kit V2 (RK20306) was used for library construction. Messenger RNA was purified from total RNA using poly-T oligo-attached magnetic beads. After fragmentation, the first strand cDNA was synthesized using random hexamer primers, followed by the second strand cDNA synthesis. The library was ready after end repair, A-tailing, adapter ligation, size selection, amplification and purification. The library was checked with Qubit and real-time PCR for quantification and bioanalyzer for size distribution detection. Quantified libraries were pooled and sequenced on Illumina platforms, according to effective library concentration and the amount of data.

An RNA-seq Snakemake (v8.25.5) workflow was used to analyze the data (10.5281/zenodo.10139567, https://github.com/niekwit/rna-seq-salmon-deseq2). After investigating the quality of the raw data using FastQC v0.12.1 (https://github.com/s-andrews/FastQC) and MultiQC v1.20 (ref. ^52^), sequence reads were trimmed to remove adaptor sequences and nucleotides with poor quality using TrimGalore v0.6.10 (10.5281/zenodo.5127898). Transcript quantification was performed with Salmon v1.10 (ref. ^53^) against the Gencode *Homo sapiens* reference transcriptome/genome (build 47) using a decoy-aware transcriptome index for accurate mapping. A mapping rate of ~90% was observed for all samples. Differential transcript analysis was performed with DESeq2 v1.44.0 (ref. ^54^). *P*-values were obtained with the Wald test and adjusted for multiple comparisons using the procedure of Benjamini–Hochberg. We modeled donor-specific effects by treating each donor as an independent batch in the design formula (~batch + treatment) during regression analysis. Genes with adjusted *P*-values < 0.05 and log_2_(fold change) > 0.5 or < −0.5 were called differentially expressed genes for each comparison. The volcano, PCA and enrichment plots were generated with a custom R script using the Cowplot v1.1.3 (https://wilkelab.org/cowplot/), DESeq2 v1.44.0, g:Profiler v0.2.3 (ref. ^55,56^) and Tidyverse v2.0.0 (10.21105/joss.01686) R packages. *P*-values for pathway enrichment were calculated and corrected from set intersections using the Set Counts and Sizes (g:SCS) method.

### Quantification and statistical analysis

Quantitative data are expressed as the mean of biological repeats ±1 s.d. or ±1 s.e.m. *P*-values were calculated using two-tailed Student’s *t*-tests, or analysis of variance (ANOVA). Data distribution was assumed to be normal, but this was not formally tested. Statistical analyses of the MS data are described in the relevant method sections. The number of biologically independent repeats is specified in figure legends. Data collection was not randomized due to the conditions of the experiments. LC–MS analyses were blinded. All other data collection and analysis were not performed blind due to the conditions of the experiments. Figures were prepared and statistical analyses performed using GraphPad Prism v9.5.1 or v10.1.1, or R v4.4.3. Software used in this study is indicated in Supplementary Table 1.

### Reporting summary

Further information on research design is available in the Nature Portfolio Reporting Summary linked to this article.

## Online content

Any methods, additional references, Nature Portfolio reporting summaries, source data, extended data, supplementary information, acknowledgements, peer review information; details of author contributions and competing interests; and statements of data and code availability are available at 10.1038/s41589-025-01965-6.

## Supplementary information

Supplementary Information Supplementary Figs. 1–7, Supplementary Tables 1 and 2 and Supporting immunoblots for Supplementary Figs. 1–7.

Reporting Summary

Supplementary Data 1 Proteomics raw data.

Supplementary Data 2 Statistical supporting data for Supplementary Fig. 1.

Supplementary Data 3 Statistical supporting data for Supplementary Fig. 2.

Supplementary Data 4 Statistical supporting data for Supplementary Fig. 3.

Supplementary Data 5 Statistical supporting data for Supplementary Fig. 4.

Supplementary Data 6 Statistical supporting data for Supplementary Fig. 5.

Supplementary Data 7 Statistical supporting data for Supplementary Fig. 6.

## Source data

Source Data Fig. 1 Unprocessed immunoblots.

Source Data Fig. 1 Statistical source data.

Source Data Fig. 2 Unprocessed immunoblots.

Source Data Fig. 2 Statistical source data.

Source Data Fig. 3 Statistical source data.

Source Data Fig. 4 Unprocessed immunoblots.

Source Data Fig. 4 Statistical source data.

Source Data Fig. 5 Unprocessed immunoblots.

Source Data Fig. 5 Statistical source data.

Source Data Fig. 6 Statistical source data.

Source Data Extended Data Fig. 1 Unprocessed immunoblots.

Source Data Extended Data Fig. 1 Statistical source data.

Source Data Extended Data Fig. 2 Statistical source data.

Source Data Extended Data Fig. 3 Unprocessed immunoblots.

Source Data Extended Data Fig. 3 Statistical source data.

Source Data Extended Data Fig. 4 Statistical source data.

Source Data Extended Data Fig. 5 Unprocessed immunoblots.

Source Data Extended Data Fig. 5 Statistical source data.

Source Data Extended Data Fig. 6 Statistical source data.

Source Data Extended Data Fig. 7 Unprocessed immunoblots.

Source Data Extended Data Fig. 7 Statistical source data.

Source Data Extended Data Fig. 8 Statistical source data.

Source Data Extended Data Fig. 9 Statistical source data.

Source Data Extended Data Fig. 10 Statistical source data.

## Data Availability

All data generated or analyzed during this study are included in the published article and supplementary files. Data from the lipoylation proteomics is shown in Supplementary Data 1. Immunoblots and all other raw data and data files from the metabolomics experiments are included in Source Data. RNA-seq data is available at the Gene Expression Omnibus (GEO): GSE292544. Data that support the findings of this study are also available from the corresponding authors upon request. Source data are provided with this paper.

## Code availability

All code used to analyze RNA-seq data and generate plots is publicly available at Zenodo (10.5281/zenodo.15051544) and GitHub (https://github.com/niekwit/2025_ML226).
