# Supplementary material for: Lipoyl deglutarylation by ABHD11 regulates mitochondrial and T cell metabolism
Source: Nat Chem Biol. 2025 Jul 15;21(12):1915–26. doi: 10.1038/s41589-025-01965-6 (PMC12643935; doi:10.1038/s41589-025-01965-6)

nature chemical biology

Supplementary information https://doi.org/10.1038/s41589-025-01965-6

Lipoyl deglutarylation by ABHD11 regulates mitochondrial and T cell metabolism

In the format provided by the authors and unedited

Supplementary Figures 1-7

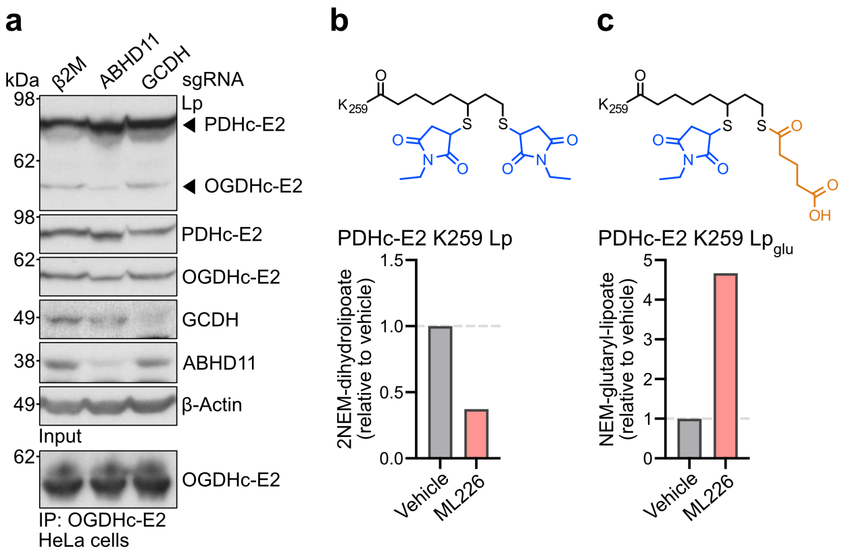

Supplementary Figure 1. Glutaryl-lipoyl adduct formation on ketoacid dehydrogenases. (a) Representative OGDHc-E2 immunoprecipitation in sgRNA-transduced HeLa cells for LC-MS/MS analysis of Lp modifications in **Fig. 1j**. **(b, c)** Effect of ABHD11 inhibition on PDHc-E2 lipoylation. HeLa cells were treated with 1 µM ML226 for 24 hr. PDHc-E2 was immunoprecipitated and K259 Lp **(b)** or Lp_{glu} **(c)** were quantified using LC-MS/MS. Peptide abundance was normalised to the total abundance of PDHc-E2 peptides and adjusted relative to the vehicle condition. *n = 1.*

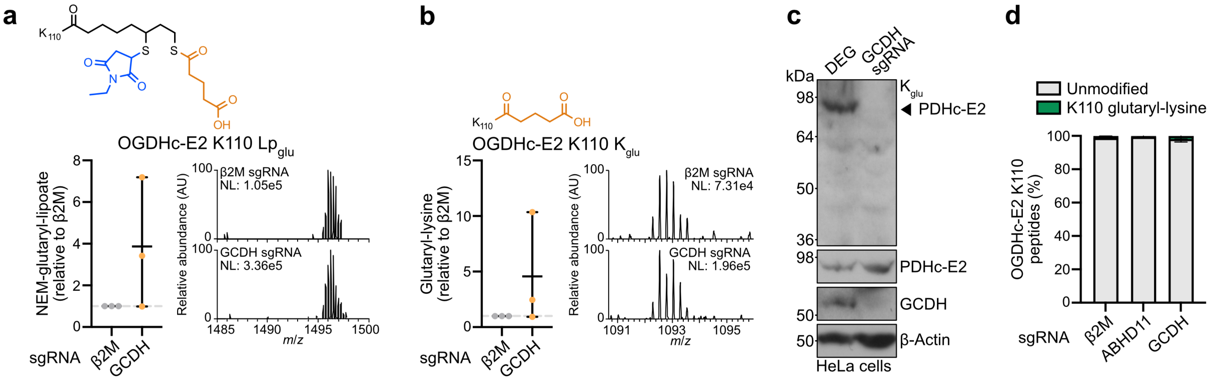

Supplementary Figure 2. Lysine glutarylation in GCDH-depleted cells. (a, b) Cas9-expressing HeLa cells were transduced with sgRNAs targeting β2M or GCDH for 11 days. OGDHc-E2 was immunoprecipitated and K110 Lp_{glu} **(a)** or K110 K_{glu} **(b)** were quantified using LC-MS/MS. Control conditions are the same as in **Fig. 1j** and **Extended Data Fig. 2b**. Peptide abundance was normalised to the total abundance of OGDHc-E2 peptides and adjusted relative to the β2M condition. Chromatograms are representative. *Mean ± SD; n = 3 independent experiments; unpaired t test, two-tailed*. **(c)** K_{glu} immunoblot in DEG-treated or GCDH-depleted HeLa cells. *Representative of n = 3 independent experiments.* **(d)** Percentage of OGDHc-E2 K110 modified by K_{glu} in **Extended Data Fig. 2b** and **(b)**. *Mean ± SD; n = 3 independent experiments.*

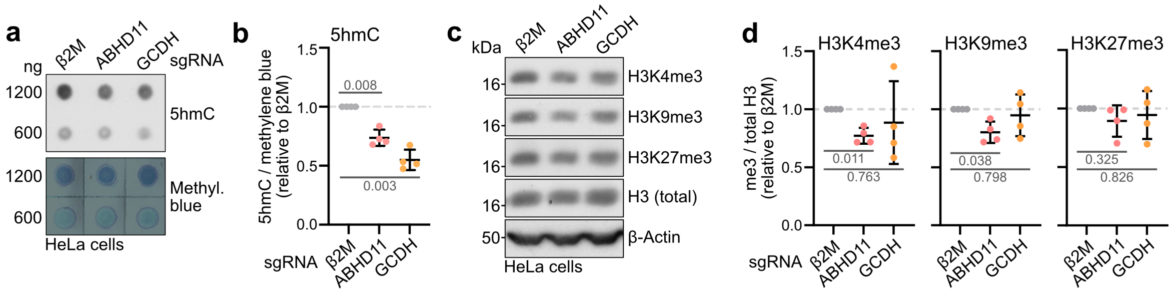

Supplementary Figure 3. Effect of ABHD11 depletion on DNA hydroxylation and histone methylation. Cas9-expressing HeLa cells were transduced with sgRNAs targeting β2M, ABHD11 or GCDH for 11 days. **(a, b)** 5hmC levels were determined using dot blotting of genomic DNA **(a)**, normalised to total DNA staining by methylene blue, and adjusted relative to the β2M condition **(b)**. *Mean ± SD; n = 4 independent experiments; one-way ANOVA and Dunnett's post-hoc test.* **(c, d)** H3K4me3, H3K9me, and H3K27me3 levels were determined using immunoblotting **(c)**. Methylation levels were normalised to total histone H3 levels and adjusted relative to the β2M condition **(d)**. *Mean ± SD; n = 4 independent experiments; one-way ANOVA and Dunnett's post-hoc test.*

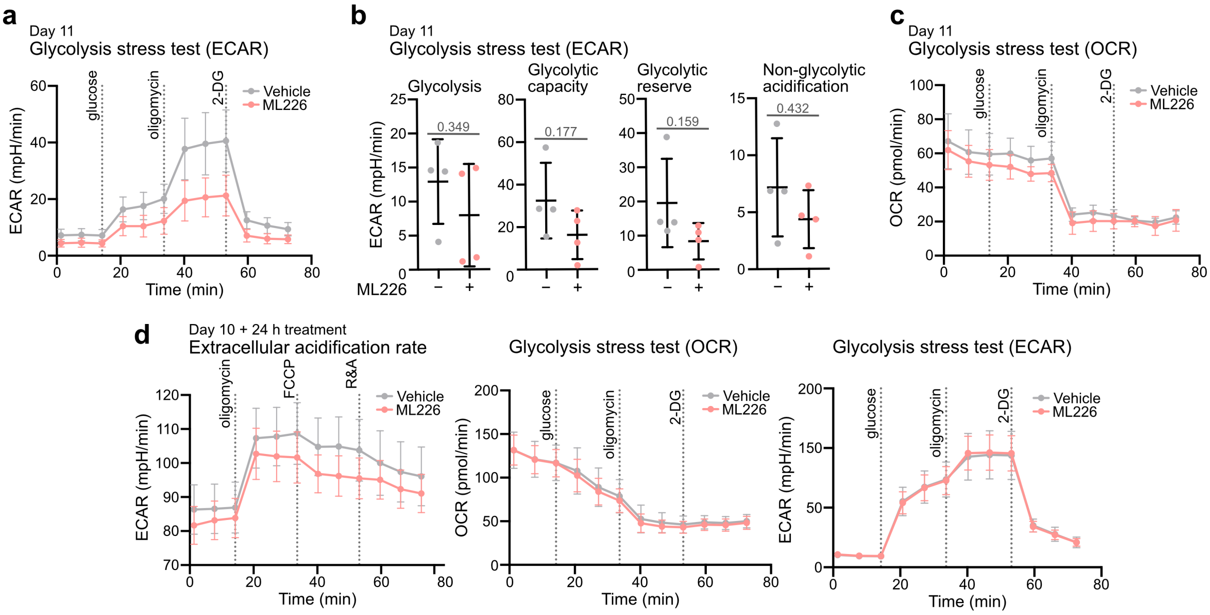

Supplementary Figure 4. Metabolic flux analysis of ML226-treated CD8+ T cells. (a-c) Human CD8$^+$ T cells were isolated from healthy donor peripheral blood mononuclear cells, activated with anti-CD3/CD28 Dynabeads, and continuously treated with 1 µM ML226 for 11 days. ECAR during a glycolysis stress test (10 mM glucose, 1 µM oligomycin, and 50 mM 2-deoxy-D-glucose (2-DG)) **(a)**. *Mean ± SEM; n = 4 donors*. Quantification of glycolysis, glycolytic capacity, glycolytic reserve, and non-glycolytic acidification **(b)**. Each data point represents one donor. *Mean ± SD; n = 4 donors; unpaired two-tailed t-test*. OCR during a glycolysis stress test **(c)**. *Mean ± SEM; n = 4 donors.* **(d)** Activated human CD8$^+$ T cells were cultured for 10 days and treated with 1 µM ML226 for 24 hr. ECAR during a mitochondrial stress test and OCR or ECAR during a glycolysis stress test were quantified. *Mean ± SEM; n = 4 donors.* All mitochondrial and glycolysis stress tests were quantified using a Seahorse XFe96 Extracellular Flux Analyzer.

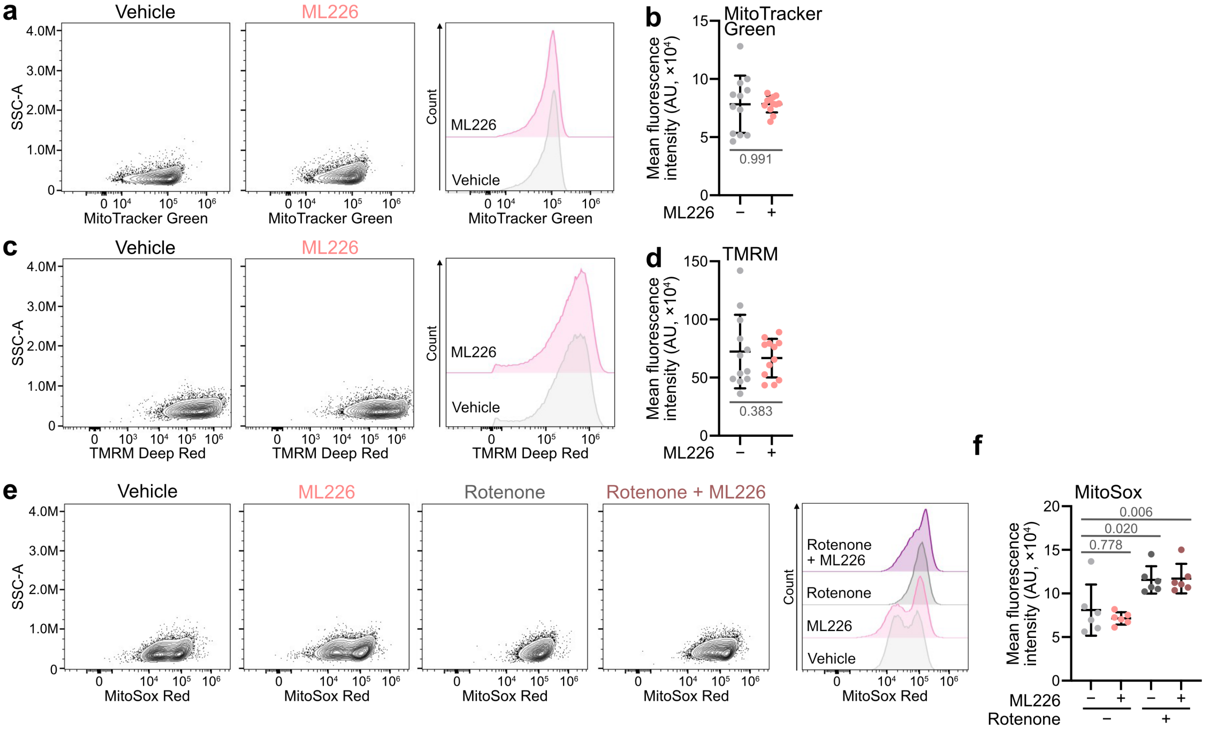

Supplementary Figure 5. Effect of ABHD11 inhibition on CD8+ T cell mitochondria. Human CD8$^+$ T cells were isolated from healthy donor peripheral blood mononuclear cells, activated with anti-CD3/CD28 Dynabeads, and continuously treated with 1 µM ML226 for 11 days. On Day 11, cells were loaded with mitochondrial florescent probes for 20 min at 37°C and analysed by flow cytometry. Each dot represents one donor. **(a, b)** MitoTracker Green staining, with representative contour plots and histogram **(a)** and quantified mean fluorescence intensity (MFI) **(b)**. *Mean ± SD; n = 12 donors*. **(c, d)** TMRM staining, with representative contour plots and histogram **(c)** and quantified MFI **(d)**. *Mean ± SD; n = 12 donors*. **(e, f)** Cells were treated with Rotenone at 37°C for 20 min prior to staining with MitoSox Red. Representative contour plots and histogram **(e)** and quantified MFI **(f)**. *Mean ± SD; n = 6 donors*; *one-way ANOVA and Dunnett's post-hoc test*.

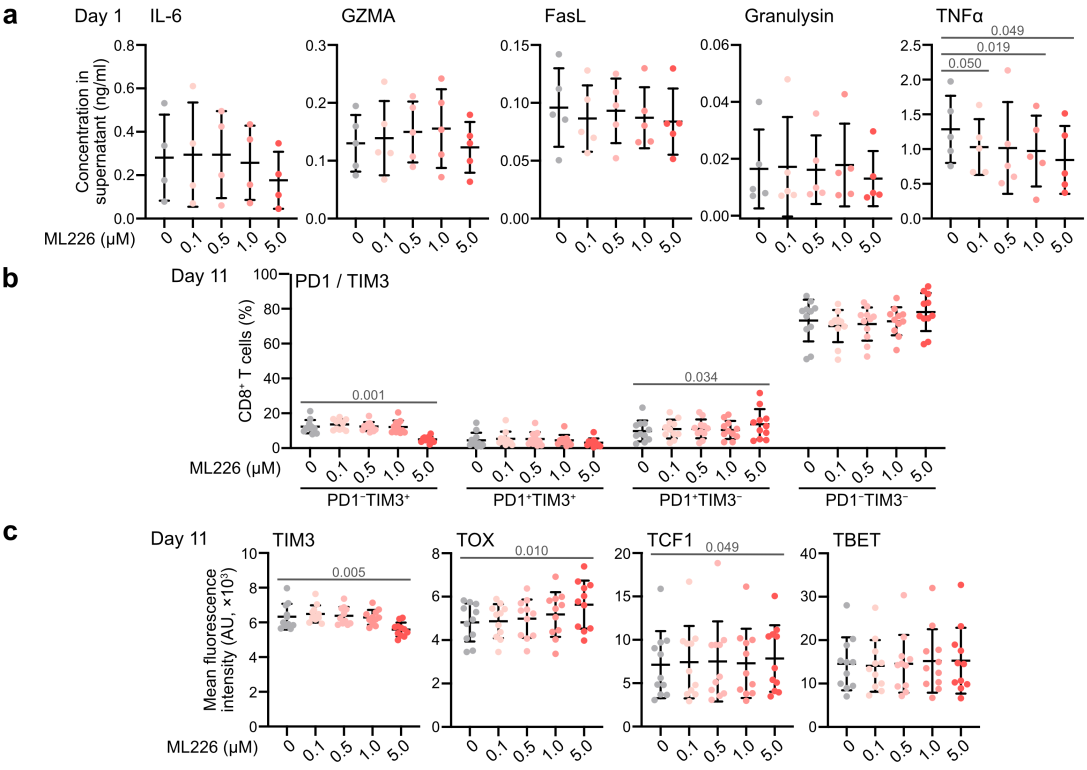

Supplementary Figure 6. Effect of ABHD11 inhibition on CD8+ T cell cytokine release and exhaustion markers. Human CD8$^+$ T cells were isolated from healthy donor peripheral blood mononuclear cells, activated with anti-CD3/CD28 Dynabeads, and continuously treated with ML226 for 11 days. **(a)** Levels of additional cytokines in cell culture supernatants from Extended Data **Fig. 9a** after 1 day were determined using a commercial multiplexed assay and flow cytometry. Each data point represents one human donor. *Mean ± SD; n = 5 donors; one-way ANOVA and Dunnett's post-hoc test.* **(b)** Percentages of PD1- and TIM3-expressing cells after 11 days of treatment with increasing doses of ML226 were determined using flow cytometry. Each data point represents one human donor. *Mean ± SD; n = 11 donors; one-way ANOVA and Dunnett's post-hoc test for each subpopulation.* **(c)** TIM3, TOX, TCF1, and TBET transcription factor levels in cells after 11 days of treatment with increasing doses of ML226 were determined using flow cytometry. Each data point represents one human donor. *Mean ± SD; n = 11 donors; one-way ANOVA and Dunnett's post-hoc test.*

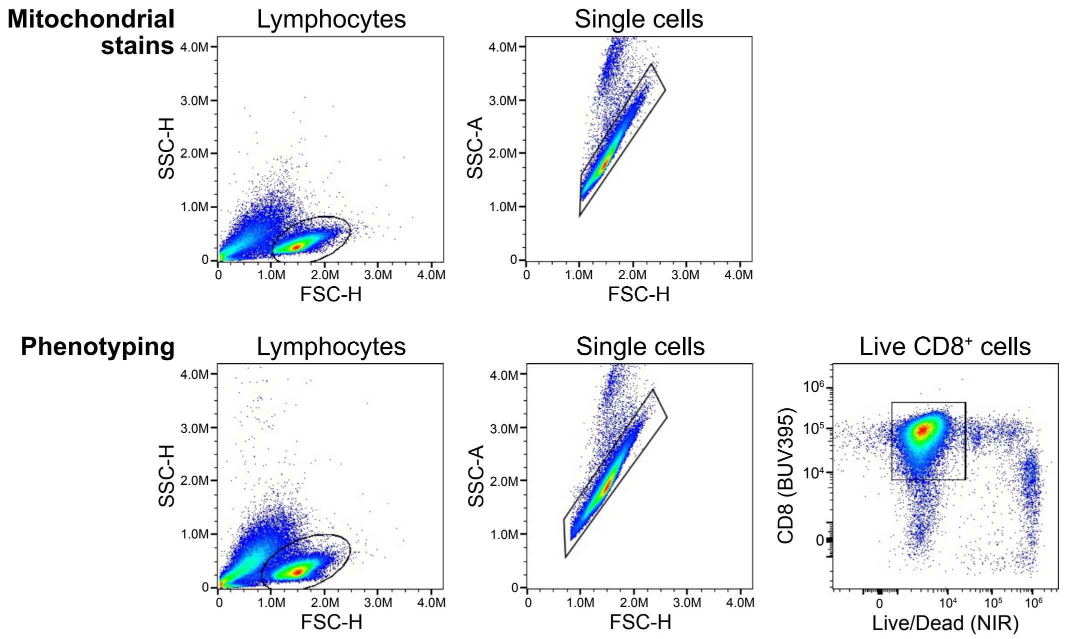

Supplementary Figure 7: Gating strategy. Representative gating strategy for the flow cytometry analysis of human lymphocytes.

Supplementary Table 1: Reagents, Antibodies, Oligonucleotide sequences and software.

Reagents

Reagent	Catalogue #	Source
ML226	Cat#25681-1mg-CAY	Cayman Chemicals
Diethylglutarate (DEG)	QB-1473	Combi-block
5,5′-dithiobis-(2-nitrobenzoic acid) (DTNB)	D8130-1G	Sigma
Glutaryl CoA (Glutaryl-CoA Salt)	G9510-5MG	Sigma
Succinyl CoA (Succinyl-CoA (sodium salt))	23297-1mg-CAY	Cayman Chemicals
CoA (Coenzyme A)	C3019-10mg	Sigma
NAD+ (β-Nicotinamide adenine dinucleotide sodium salt)	N0632-1G	Sigma
Dimethyl 2-oxoglutarate (DM-2OG)	349631-5G	Sigma
Dimethyloxalylglycine (DMOG)	71210-100 mg-CAY	Cambridge Bioscience
N-ethylmaleimide (NEM)	E3876-5G	Sigma
Nicotinamide	72340-100G	Sigma
Puromycin	100552-25MG	MP Biomedicals
(±)-3-Methyl-2-oxovaleric acid sodium salt	K7125	Sigma-Aldrich
Cycloheximide (CHX)	C7698	Sigma-Aldrich
VH-298	6156	Tocris Bioscience
(±)-3-Methyl-2-oxovaleric acid sodium salt (KMV)	K7125	Sigma-Aldrich
Dimethyl 2-oxoglutarate (DM-2OG)	349631	Sigma-Aldrich
MitoSox	M36008	ThermoFisher
TMRM	M7514	ThermoFisher
MitoTracker	T668	ThermoFisher

Rotenone	13885	Cayman Chemicals

SgRNA sequences

Target	sgRNA sequence
β2M	GGCCGAGATGTCTCGCTCCG
ABHD11	AAGATCTTGGCCCAGCAGAC
GCDH (1)<br>**GCDH (2)**<br>GCDH (3)	GAGGCCGTGAACACCTACGA<br>**CAGCAGAGAAACCATCTCGG**<br>GATAGGGTGCATGACGAGGG

Antibodies

**Antibodies for immunoprecipitation and immunoblotting**		
**Antibody**	**Source, Catalogue #**	**Working dilution**
β-Actin (mouse monoclonal)	Sigma-Aldrich A2228	1:20,000 in 2% BSA in PBST
5-hydroxymethylcytosine (rabbit serum)	Active Motif 39769	1:10,000 in 2% BSA in PBST
ABHD11 (rabbit polyclonal)	Enogene E11-14208C	1:2,000 in 2% BSA in PBST
Flag M2 (mouse monoclonal)	Sigma F3165	1:4,000 in 2% BSA in PBST
GCDH (rabbit polyclonal)	Proteintech 14930-1-AP	1:2,000 in 5% skim milk in PBST
Glutaryl-lysine (rabbit polyclonal)	PTM Bio PTM-1151	1:2,000 in 5% skim milk in PBST
H3 (D1H2) (rabbit monoclonal)	Cell Signaling Technology 4499	1:2,000 in 2% BSA in PBST
H3K27me3 (C36B11) (rabbit monoclonal)	Cell Signaling Technology 9733	1:1,000 in 2% BSA in PBST
H3K4me3 (C42D8) (rabbit monoclonal)	Cell Signaling Technology 9751	1:1,000 in 2% BSA in PBST
H3K9me3 (D4W1U) (rabbit monoclonal)	Cell Signaling Technology 13969	1:1,000 in 2% BSA in PBST
HIF1α (D1S7W) (rabbit monoclonal)	Cell Signaling Technology 36169	1:2,000 in 2% BSA in PBST
Hydroxy-HIF1α (Pro564) (D43B5) (rabbit monoclonal)	Cell Signaling Technology 3434	1:1,000 in 5% BSA in TBS + 0.1% Tween-20
Lipoic acid (rabbit polyclonal)	Sigma Aldrich 437695	1:2,000 in 2% fatty acid-free BSA in PBST
OGDHc-E2 / DLST (9F4BD5) (mouse monoclonal)	Abcam ab110306	10 μl per 50×10^6 cells for immunoprecipitation
OGDHc-E2 / DLST (D22B1) (rabbit monoclonal)	Cell Signaling Technology 11954	1:2,000 in 2% BSA in PBST

PDHc-E2 / DLAT (4A4-B6-C10) (mouse monoclonal)	Cell Signaling Technology 12362	1:2,000 in 2% BSA in PBST ; 10 μl per 50×10^6 cells for immunoprecipitation
Peroxidase Goat Anti-Mouse IgG	Jackson ImmunoResearch 115-035-146	1:20,000 in 2% BSA in PBST
Peroxidase Goat Anti-Rabbit IgG	Jackson ImmunoResearch 115-035-045	1:20,000 in 2% BSA in PBST
**Flow cytometry antibodies**		
**Antibody**	**Fluorophore, Source, Catalogue #**	**Working dilution**
CD8 (RPA-T8)	BUV395<br>BD Biosciences 563796	1:400
CD45RO (UCHL1)	BUV495<br>BD Biosciences 749888	1:200
CD25 (BC96)	BV510 Biolegend 302639	1:200
CD45RA (HI101)	BV650 Biolegend 304135	1:200
CD62L (DREG-56)	AF488 Biolegend 304816	1:200
CD62L (DREG-56)	PerCP/Cy5.5<br>Biolegend 304824	1:200
CCR7 (3D12)	PE/Cy7<br>BD Biosciences 560922	1:100
CD27 (LG.3A10)	AF700 Biolegend 124239	1:200
TIM3 (F38-2E2)	BV605 Biolegend 345017	1:100
PD1 (EH12.2H7)	AF488 Biolegend 329936	1:100
TCF1 (S33-966)	PE BD Biosciences 564217	1:100
LAG3 (11C3C65)	AF647 Biolegend 369304	1:100
Perforin (dG9)	Pacific Blue<br>Biolegend 308117	1:100
Granzyme B (AD2)	PerCP/Cy5.5<br>Biolegend 344013	1:100
TBET (4B10)	PE/Dazzle594<br>Biolegend 644828	1:100
TIGIT (A15153G)	PE/Cy7 Biolegend 372713	1:200
TOX (TXRX10)	eflour660<br>eBiosciences 50-6502-82	1:100
CCR7 (G043H7)	BV421 Biolegend 353208	1:100
CD45RA (H100)	BV605 Biolegend 304135	1:200
CD95 (DX2)	AF488 Biolegend 305615	1:200
CD95 (DX2)	Pe-Cy7 Biolegend 305621	1:200
CD28 (CD28.2)	AF647 Biolegend 302953	1:200
CD27 (M-T271)	PE Biolegend 356405	1:200

Quantitative PCR primers

Target gene	Forward primer	Reverse primer
ACTB	CTGGGAGTGGGTGGAGGC	TCAACTGGTCTCAAGTCAGTG

CAIX	GCCGCCTTTCTGGAGGA	TCTTCCAAGCGAGACAGCAA
VEGF	TACCTCCACCATGCCAAGTG	ATGATTCTGCCCTCCTCCTTC
BAP1	GGAGGTAGAGAAGAGGAAGAA	GAGCCAGCATGGAGATAAAG
DLST (OGDHc-E2)	AGATGAGGGCTCGGCACAAAGA	CCACCTCTTTGGTTGTGTCGTC

Software

Software	Source	Identifier
FlowJo v10.9, v10.10	BD Biosciences	RRID:SCR_008520
PEAKS 11	Bioinfor	https://www.bioinfor.com/peaks-11
PEAKS Studio v8.0	Bioinformatics Solutions Inc	RRID:SCR_022841
Prism v9.5.1, v10.1.1	GraphPad	RRID:SCR_002798
proMod3 v3.3.0	swissmodel.expasy.org	
PyMOL v3.0.4	Pymol	RRID:SCR_000305
Coot v0.9.8.8 (EL) CCP4i	https://www2.mrc-lmb.cam.ac.uk/personal/pemsley/coot/	RRID:SCR_014222
SHELX (CCP4i)	http://shelx.uni-ac.gwdg.de/SHELX/	RRID:SCR_014220
HADDOCK2.4	https://rascar.science.uu.nl/haddock2.4/	RRID:SCR_019091
Image Studio Lite v5.5	LI-COR Biosciences	RRID:SCR_013715
ImageJ v1.54g	ImageJ	RRID:SCR_003070
R v4.4.3	https://www.r-project.org	RRID:SCR_001905
R Studio 2024.12.0 Build 467	Posit PBC	RRID:SCR_000432
tidyverse v2.0.0	https://www.tidyverse.org	RRID:SCR_019186
Cowplot v1.1.3	https://wilkelab.org/cowplot/articles/introduction.html	RRID:SCR_018081
gprofiler2 v0.2.3	https://cran.r-project.org/web/packages/gprofiler2/index.html	RRID:SCR_018190
Snakemake v8.25.5	https://snakemake.github.io	RRID:SCR_003475
DESeq2 v1.44.0	https://bioconductor.org/packages/release/bioc/html/DESeq2.html	RRID:SCR_015687
Salmon v1.10	https://salmon.readthedocs.io/en/latest/salmon.html	RRID:SCR_017036
FastQC v0.12.1	https://github.com/s-andrews/FastQC	RRID:SCR_014583
MultiQC v1.20	https://github.com/MultiQC/MultiQC	RRID:SCR_014982

TrimGalore v0.6.10	https://github.com/FelixKrueger/TrimGalore	RRID:SCR_011847
MassHunter Profinder v10.0	Agilent	RRID:SCR_016657
Skyline v24.1	https://skyline.ms/project/home/software/Skyline/begin.view	RRID:SCR_014080

Supplementary Table 2: LC-MS chromatographic gradients

LC-MS chromatographic gradient for metabolite quantification in HeLa cells

Minute	%B	Flow rate (ml/min)
0.0	85	450
3.0	79	450
5.5	60	450
7.0	50	450
8.5	50	450
8.7	85	450
12.5	85	450

LC-MS chromatographic gradient for metabolite quantification in CD8+ T cells

Minute	%B	Flow rate (ml/min)
0.0	20	0.2
3.0	50	0.2
18.00	95	0.2
25.00	95	0.2
26.00	20	0.25
29.00	20	0.25
30.00	20	0.25

Supplementary source data: immunoblots for Supplementary Figures 1-7

Supplementary Figure 1a

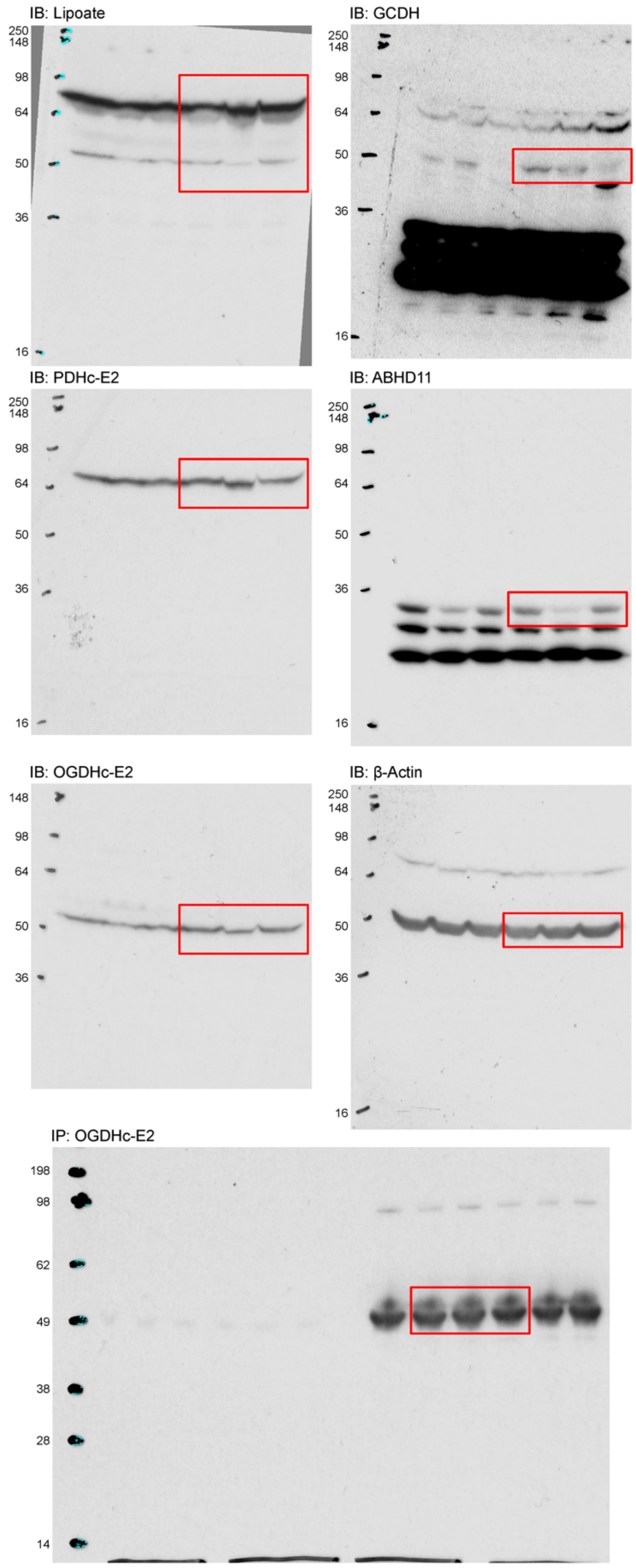

Supplementary Figure 2c

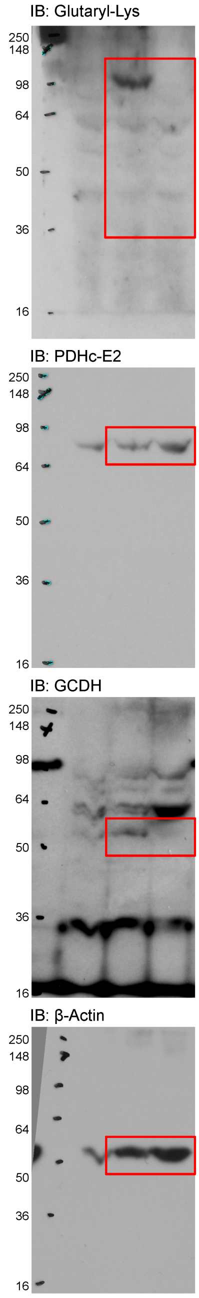

Supplementary Figure 3a

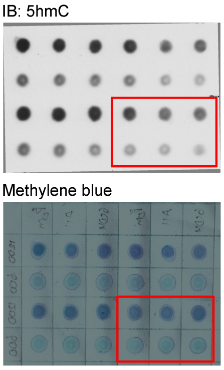

Supplementary Figure 3c

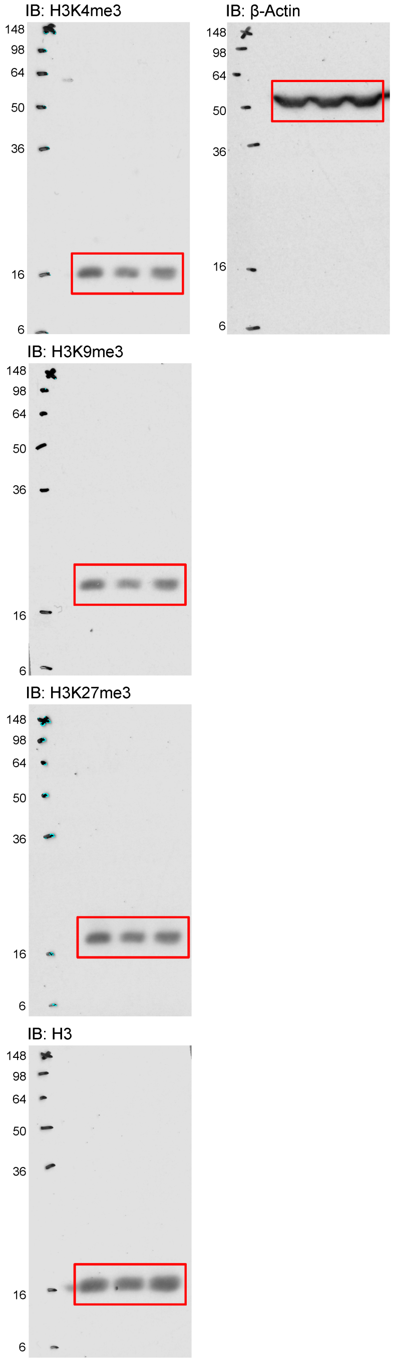

Supplement: Supplementary file 1 — Supplementary Figs. 1–7, Supplementary Tables 1 and 2 and Supporting immunoblots for Supplementary Figs. 1–7. [file 41589_2025_1965_MOESM1_ESM.pdf]
