# Supplementary material for: Lipoyl deglutarylation by ABHD11 regulates mitochondrial and T cell metabolism
Source: Nat Chem Biol. 2025 Jul 15;21(12):1915–26. doi: 10.1038/s41589-025-01965-6 (PMC12643935; doi:10.1038/s41589-025-01965-6)
Supplement: Supplementary file 2 — Reporting Summary [file 41589_2025_1965_MOESM2_ESM.pdf]

nature portfolio

Corresponding author(s): James A Nathan, Randall S Johnson

Last updated by author(s): May 23rd 2025

Reporting Summary

Nature Portfolio wishes to improve the reproducibility of the work that we publish. This form provides structure for consistency and transparency in reporting. For further information on Nature Portfolio policies, see our Editorial Policies and the Editorial Policy Checklist.

Statistics

For all statistical analyses, confirm that the following items are present in the figure legend, table legend, main text, or Methods section.

n/a	Confirmed	
☐	☒	The exact sample size (*n*) for each experimental group/condition, given as a discrete number and unit of measurement
☐	☒	A statement on whether measurements were taken from distinct samples or whether the same sample was measured repeatedly
☐	☒	The statistical test(s) used AND whether they are one- or two-sided<br>*Only common tests should be described solely by name; describe more complex techniques in the Methods section.*
☐	☒	A description of all covariates tested
☐	☒	A description of any assumptions or corrections, such as tests of normality and adjustment for multiple comparisons
☐	☒	A full description of the statistical parameters including central tendency (e.g. means) or other basic estimates (e.g. regression coefficient) AND variation (e.g. standard deviation) or associated estimates of uncertainty (e.g. confidence intervals)
☐	☒	For null hypothesis testing, the test statistic (e.g. *F*, *t*, *r*) with confidence intervals, effect sizes, degrees of freedom and *P* value noted<br>*Give P values as exact values whenever suitable.*
☒	☐	For Bayesian analysis, information on the choice of priors and Markov chain Monte Carlo settings
☒	☐	For hierarchical and complex designs, identification of the appropriate level for tests and full reporting of outcomes
☒	☐	Estimates of effect sizes (e.g. Cohen's *d*, Pearson's *r*), indicating how they were calculated

Our web collection on statistics for biologists contains articles on many of the points above.

Software and code

Policy information about availability of computer code

Data collection	Plate reader: CLARIOstar Plus (0430)<br>Auora (Cytex Biosciences); Waters Acquity UPLC system coupled to a Xevo-TQ-A mass spectrometer (Waters, Milford, MA, USA); Seahorse XFe bioanalyser (Agilent), Vanquish U-HPLC system (Thermo Fisher) coupled to a Q-Exactive HF-X mass spectrometer utilizing a HESI probe (Thermo Fisher), Kinetex evo C18 column (2.6 um, 150 mm × 2.0 mm I.D., Phenomenex) and coupled to an Agilent 6546 LC/Q-TOF
Data analysis	Immunoblot quantification: ImageJ 1.54g, Image Studio Lite v5.5 (LICOR Biosciences)<br>GraphPad Prism version v9.5.1 and v10.1.1<br>PEAKS 11<br>PEAKS Studio v8.0<br>proMod3 v3.3.0<br>PyMOL v3.0.4<br>Coot v0.9.8.8 (EL) CCP4i<br>SHELX (CCP4i)<br>HADDOCK2.4<br>FlowJo v10.9 and v10.10<br>tidyverse v2.0.0<br>Cowplot v1.1.3<br>g:profiler2 v0.2.3<br>Snakemake v8.25.5<br>DESeq2 v1.44.0<br>Salmon v1.10

FastQC v0.12.1
MultiQC v1.20
TrimGalore v0.6.10
R v4.4.3
R Studio 2024.12.0 Build 467
Agilent MassHunter Profinder v10.0
Skyline v24.1

For manuscripts utilizing custom algorithms or software that are central to the research but not yet described in published literature, software must be made available to editors and reviewers. We strongly encourage code deposition in a community repository (e.g. GitHub). See the Nature Portfolio guidelines for submitting code & software for further information.

Data

Policy information about availability of data

All manuscripts must include a data availability statement. This statement should provide the following information, where applicable:

- Accession codes, unique identifiers, or web links for publicly available datasets
- A description of any restrictions on data availability
- For clinical datasets or third party data, please ensure that the statement adheres to our policy

All data generated or analyzed during this study are included in the published article and supplementary files. Data from the lipoylation proteomics is shown in Supplementary Data 1. Immunoblots are available in the Source Data. All other raw data and data files from the metabolomics experiments are included in Source Data. RNA-seq data is available at the Gene Expression Omnibus (GEO): GSE292544. Data that support the findings of this study are also available from the corresponding authors upon reasonable request.

Human research participants

Policy information about studies involving human research participants and Sex and Gender in Research.

Reporting on sex and gender	Sex and gender of healthy volunteer blood donors was not obtained as per ethical approval requirements.
Population characteristics	Human peripheral blood mononuclear cells (PBMCs) were obtained from National Health Services (NHS) Blood and Transplant (NHSBT: Addenbrooke's Hospital, Cambridge, United Kingdom) and Boston Children's Blood Donation Clinic, Boston, MA, USA. All volunteers were healthy as defined by national blood donation standards and over the age of 18 years.
Recruitment	Human peripheral blood mononuclear cells (PBMCs) were obtained from National Health Services (NHS) Blood and Transplant (NHSBT: Addenbrooke's Hospital, Cambridge, United Kingdom) and Boston Children's Blood Donation Clinic, Boston, MA, USA from healthy volunteers.
Ethics oversight	Ethical approval was obtained from the East of England-Cambridge Central Research Ethics Committee (06/Q0108/281) nd Boston Children's Blood Donation Clinic, Boston, MA, USA.

Note that full information on the approval of the study protocol must also be provided in the manuscript.

Field-specific reporting

Please select the one below that is the best fit for your research. If you are not sure, read the appropriate sections before making your selection.

☒ Life sciences ☐ Behavioural & social sciences ☐ Ecological, evolutionary & environmental sciences

For a reference copy of the document with all sections, see nature.com/documents/nr-reporting-summary-flat.pdf

Life sciences study design

All studies must disclose on these points even when the disclosure is negative.

Sample size	No sample-size calculation was performed. Based on pilot experiments and their variability, the number of experiments was determined.
Data exclusions	Data points identified as outliers by independent statistical analysis were excluded. For the majority of datasets, data points identified as outliers were excluded based on the ROUT test (Q = 1%), the default method implemented in GraphPad Prism. This exclusion criterion was pre-established prior to analysis to minimize the impact of extreme values likely due to technical variation or measurement error, thereby ensuring more robust and reliable statistical comparisons. For the RNA-seq analysis, sample-level outliers were identified and excluded based on principal component analysis (PCA). Samples that clustered separately from their respective experimental groups and were inconsistent with expected biological variation were excluded prior to downstream analysis. This PCA-based exclusion was also defined as part of our standard quality control workflow before differential expression analysis.
Replication	All experiments were replicated in at least three independent studies (specific details provided in the methods under Quantification and statistical analysis).

Randomization	Human blood was provided at random selection by the blood donation clinic. Investigators were not provided information about the individual donors. Therefore, these samples were randomly allocated to be treated with or without ML226 in each experimental group. Biochemical experiments were not randomised as this was not possible.
Blinding	LC-MS analyses were blinded. All other data collection and analysis were not performed blind to the conditions of the experiments and comparisons between samples. Western blotting could not be blinded as it was necessary to know what the sample contained to allow comparisons by SDS-PAGE.

Behavioural & social sciences study design

All studies must disclose on these points even when the disclosure is negative.

Study description	*Briefly describe the study type including whether data are quantitative, qualitative, or mixed-methods (e.g. qualitative cross-sectional, quantitative experimental, mixed-methods case study).*
Research sample	*State the research sample (e.g. Harvard university undergraduates, villagers in rural India) and provide relevant demographic information (e.g. age, sex) and indicate whether the sample is representative. Provide a rationale for the study sample chosen. For studies involving existing datasets, please describe the dataset and source.*
Sampling strategy	*Describe the sampling procedure (e.g. random, snowball, stratified, convenience). Describe the statistical methods that were used to predetermine sample size OR if no sample-size calculation was performed, describe how sample sizes were chosen and provide a rationale for why these sample sizes are sufficient. For qualitative data, please indicate whether data saturation was considered, and what criteria were used to decide that no further sampling was needed.*
Data collection	*Provide details about the data collection procedure, including the instruments or devices used to record the data (e.g. pen and paper, computer, eye tracker, video or audio equipment) whether anyone was present besides the participant(s) and the researcher, and whether the researcher was blind to experimental condition and/or the study hypothesis during data collection.*
Timing	*Indicate the start and stop dates of data collection. If there is a gap between collection periods, state the dates for each sample cohort.*
Data exclusions	*If no data were excluded from the analyses, state so OR if data were excluded, provide the exact number of exclusions and the rationale behind them, indicating whether exclusion criteria were pre-established.*
Non-participation	*State how many participants dropped out/declined participation and the reason(s) given OR provide response rate OR state that no participants dropped out/declined participation.*
Randomization	*If participants were not allocated into experimental groups, state so OR describe how participants were allocated to groups, and if allocation was not random, describe how covariates were controlled.*

Ecological, evolutionary & environmental sciences study design

All studies must disclose on these points even when the disclosure is negative.

Study description	*Briefly describe the study. For quantitative data include treatment factors and interactions, design structure (e.g. factorial, nested, hierarchical), nature and number of experimental units and replicates.*
Research sample	*Describe the research sample (e.g. a group of tagged Passer domesticus, all Stenocereus thurberi within Organ Pipe Cactus National Monument), and provide a rationale for the sample choice. When relevant, describe the organism taxa, source, sex, age range and any manipulations. State what population the sample is meant to represent when applicable. For studies involving existing datasets, describe the data and its source.*
Sampling strategy	*Note the sampling procedure. Describe the statistical methods that were used to predetermine sample size OR if no sample-size calculation was performed, describe how sample sizes were chosen and provide a rationale for why these sample sizes are sufficient.*
Data collection	*Describe the data collection procedure, including who recorded the data and how.*
Timing and spatial scale	*Indicate the start and stop dates of data collection, noting the frequency and periodicity of sampling and providing a rationale for these choices. If there is a gap between collection periods, state the dates for each sample cohort. Specify the spatial scale from which the data are taken*
Data exclusions	*If no data were excluded from the analyses, state so OR if data were excluded, describe the exclusions and the rationale behind them, indicating whether exclusion criteria were pre-established.*
Reproducibility	*Describe the measures taken to verify the reproducibility of experimental findings. For each experiment, note whether any attempts to repeat the experiment failed OR state that all attempts to repeat the experiment were successful.*

Randomization

Describe how samples/organisms/participants were allocated into groups. If allocation was not random, describe how covariates were controlled. If this is not relevant to your study, explain why.

Blinding

Describe the extent of blinding used during data acquisition and analysis. If blinding was not possible, describe why OR explain why blinding was not relevant to your study.

Did the study involve field work? ☐ Yes ☐ No

Field work, collection and transport

Field conditions

Describe the study conditions for field work, providing relevant parameters (e.g. temperature, rainfall).

Location

State the location of the sampling or experiment, providing relevant parameters (e.g. latitude and longitude, elevation, water depth).

Access & import/export

Describe the efforts you have made to access habitats and to collect and import/export your samples in a responsible manner and in compliance with local, national and international laws, noting any permits that were obtained (give the name of the issuing authority, the date of issue, and any identifying information).

Disturbance

Describe any disturbance caused by the study and how it was minimized.

Reporting for specific materials, systems and methods

We require information from authors about some types of materials, experimental systems and methods used in many studies. Here, indicate whether each material, system or method listed is relevant to your study. If you are not sure if a list item applies to your research, read the appropriate section before selecting a response.

Materials & experimental systems

n/a	Involved in the study
☐	☒ Antibodies
☐	☒ Eukaryotic cell lines
☒	☐ Palaeontology and archaeology
☒	☐ Animals and other organisms
☒	☐ Clinical data
☒	☐ Dual use research of concern

Methods

n/a	Involved in the study
☒	☐ ChIP-seq
☐	☒ Flow cytometry
☒	☐ MRI-based neuroimaging

Antibodies

Antibodies used

Details of primary and secondary antibodies are also described in the Supplementary Data.
β-Actin Sigma-Aldrich A2228 1:20,000
5-hydroxymethylcytosine Active Motif 39769 1:10,000
ABHD11 Enogene E11-14208C 1:2,000
Flag M2 Sigma F3165 1:4,000
GCDH Proteintech 14930-1-AP 1:2,000
Glutaryl-lysine PTM Bio PTM-1151 1:2,000
H3 (D1H2) Cell Signaling Technology 4499 1:2,000
H3K27me3 (C36B11) Cell Signaling Technology 9733 1:1,000
H3K4me3 (C42D8) Cell Signaling Technology 9751 1:1,000
H3K9me3 (D4W1U) Cell Signaling Technology 13969 1:1,000
HIF1α (D1S7W) Cell Signaling Technology 36169 1:2,000
Hydroxy-HIF1α (Pro564) (D43B5) Cell Signaling Technology 3434 1:1,000
Lipoic acid Sigma Aldrich 437695 1:2,000
OGDHc-E2 / DLST (9F4BD5) Abcam ab110306 10 µl per 50×106 cells for immunoprecipitation
OGDHc-E2 / DLST (D22B1) Cell Signaling Technology 11954 1:2,000
PDHc-E2 / DLAT (4A4-B6-C10) Cell Signaling Technology 12362 1:2,000 or 10 µl per 50×106 cells for immunoprecipitation
Peroxidase Goat Anti-Mouse IgG Jackson ImmunoResearch 115-035-146 1:20,000
Peroxidase Goat Anti-Rabbit IgG Jackson ImmunoResearch 115-035-045 1:20,000
CD8 (RPA-T8) BUV395 BD Biosciences 563796 1:400
CD45RO (UCHL1) BUV495 BD Biosciences 749888 1:200
CD25 (BC96) BV510 Biolegend 302639 1:200
CD45RA (HI101) BV650 Biolegend 304135 1:200
CD62L (DREG-56) AF488 Biolegend 304816 1:200
CD62L (DREG-56) PerCP/Cy5.5 Biolegend 304824 1:200

CCR7 (3D12) PE/Cy7 BD Biosciences 560922 1:100
CD27 (LG.3A10) AF700 Biolegend 124239 1:200
TIM3 (F38-2E2) BV605 Biolegend 345017 1:100
PD1 (EH12.2H7) AF488 Biolegend 329936 1:100
TCF1 (S33-966) PE BD Biosciences 564217 1:100
LAG3 (11C3C65) AF647 Biolegend 369304 1:100
Perforin (dG9) Pacific Blue Biolegend 308117 1:100
Granzyme B (AD2) PerCP/Cy5.5 Biolegend 344013 1:100
TBET (4B10) PE/Dazzle594 Biolegend 644828 1:100
TIGIT (A15153G) PE/Cy7 Biolegend 372713 1:200
TOX (TXRX10) eflour660 eBiosciences 50-6502-82 1:100
CCR7 (G043H7) BV421 BioLegend 353208 1:100
CD45RA (HI100) BV605 BioLegend 304135 1:200
CD95 (DX2) AF488 BioLegend 305615 1:200
CD95 (DX2) Pe-Cy7 BioLegend 305621 1:200
CD28 (CD28.2) AF647 BioLegend 302953 1:200
CD27 (M-T271) PE BioLegend 356405 1:200

Validation

All antibodies were purchased from commercial companies as indicated. Antibodies were validated by sited companies. No unvalidated antibody was used in this study.
β-Actin, immunoblotting (human) - manuscript data
5-hydroxymethylcytosine, dot blotting (human) - manuscript data
ABHD11, immunoblotting (human) - manuscript data
Flag M2, immunoblotting (human) - manuscript data
GCDH, immunoblotting (human) - manuscript data
Glutaryl-lysine, immunoblotting (human) - manuscript data
H3 (D1H2), immunoblotting (human) - manuscript data
H3K27me3 (C36B11), immunoblotting (human) - manuscript data
H3K4me3 (C42D8), immunoblotting (human) - manuscript data
H3K9me3 (D4W1U), immunoblotting (human) - manuscript data
HIF1α (D1S7W), immunoblotting (human) - manuscript data
Hydroxy-HIF1α (Pro564) (D43B5), immunoblotting (human) - manuscript data
Lipoic acid, immunoblotting (human) - manuscript data
OGDHc-E2 / DLST (9F4BD5), IP, (human) - manuscript data
OGDHc-E2 / DLST (D22B1), immunoblotting (human) - manuscript data
PDHc-E2 / DLAT (4A4-B6-C10), immunoblotting and IP, (human) - manuscript data
Peroxidase Goat Anti-Mouse IgG Jackson ImmunoResearch 115-035-146, immunoblotting (human) - manuscript data
Peroxidase Goat Anti-Rabbit IgG Jackson ImmunoResearch 115-035-045, immunoblotting (human) - manuscript data
CD8 (RPA-T8) BUV395 BD Biosciences 563796, flow cytometry (human) - manuscript data
CD45RO (UCHL1) BUV495 BD Biosciences 749888, flow cytometry (human) - manuscript data
CD25 (BC96) BV510 Biolegend 302639, flow cytometry (human) - manuscript data
CD45RA (HI101) BV650 Biolegend 304135, flow cytometry (human) - manuscript data
CD62L (DREG-56) AF488 Biolegend 304816, flow cytometry (human) - manuscript data
CD62L (DREG-56) PerCP/Cy5.5 Biolegend 304824, flow cytometry (human) - manuscript data
CCR7 (3D12) PE/Cy7 BD Biosciences 560922, flow cytometry (human) - manuscript data
CD27 (LG.3A10) AF700 Biolegend 124239, flow cytometry (human) - manuscript data
TIM3 (F38-2E2) BV605 Biolegend 345017, flow cytometry (human) - manuscript data
PD1 (EH12.2H7) AF488 Biolegend 329936, flow cytometry (human) - manuscript data
TCF1 (S33-966) PE BD Biosciences 564217, flow cytometry (human) - manuscript data
LAG3 (11C3C65) AF647 Biolegend 369304, flow cytometry (human) - manuscript data
Perforin (dG9) Pacific Blue Biolegend 308117, flow cytometry (human) - manuscript data
Granzyme B (AD2) PerCP/Cy5.5 Biolegend 344013, flow cytometry (human) - manuscript data
TBET (4B10) PE/Dazzle594 Biolegend 644828, flow cytometry (human) - manuscript data
TIGIT (A15153G) PE/Cy7 Biolegend 372713, flow cytometry (human) - manuscript data
TOX (TXRX10) eflour660 eBiosciences 50-6502-82, flow cytometry (human) - manuscript data
CCR7 (G043H7) BV421 BioLegend 353208, flow cytometry (human) - manuscript data
CD45RA (HI100) BV605 BioLegend 304135, flow cytometry (human) - manuscript data
CD95 (DX2) AF488 BioLegend 305615, flow cytometry (human) - manuscript data
CD95 (DX2) Pe-Cy7 BioLegend 305621, flow cytometry (human) - manuscript data
CD28 (CD28.2) AF647 BioLegend 302953, flow cytometry (human) - manuscript data

Eukaryotic cell lines

Policy information about cell lines and Sex and Gender in Research

Cell line source(s)

HeLa (gift from Paul Lehner, purchased from ATCC), HEK293T (purchased from ATCC)

Authentication

All cells were authenticated by Eurofins as follows: genetic characteristics were determined by PCR-single-locus-technology. 21 independent PCR-systems Amelogenin, D3S1358, D1S1656, D6S1043, D13S317, Penta E, D16S539, D18S51, D2S1338, CSF1PO, Penta D, TH01, vWA, D21S11, D7S820, D5S818, TPOX, D8S1179, D12S391, D19S433 and FGA were investigated (Promega, PowerPlex 21 PCR Kit). In parallel, positive and negative controls were carried out yielding correct results.

Mycoplasma contamination

All cell lines tested negative for Mycoplasma contamination.

Commonly misidentified lines (See ICLAC register)	None

Palaeontology and Archaeology

Specimen provenance	*Provide provenance information for specimens and describe permits that were obtained for the work (including the name of the issuing authority, the date of issue, and any identifying information). Permits should encompass collection and, where applicable, export.*
Specimen deposition	*Indicate where the specimens have been deposited to permit free access by other researchers.*
Dating methods	*If new dates are provided, describe how they were obtained (e.g. collection, storage, sample pretreatment and measurement), where they were obtained (i.e. lab name), the calibration program and the protocol for quality assurance OR state that no new dates are provided.*

☐ Tick this box to confirm that the raw and calibrated dates are available in the paper or in Supplementary Information.

Ethics oversight	*Identify the organization(s) that approved or provided guidance on the study protocol, OR state that no ethical approval or guidance was required and explain why not.*

Note that full information on the approval of the study protocol must also be provided in the manuscript.

Animals and other research organisms

Policy information about studies involving animals; ARRIVE guidelines recommended for reporting animal research, and Sex and Gender in Research

Laboratory animals	*For laboratory animals, report species, strain and age OR state that the study did not involve laboratory animals.*
Wild animals	*Provide details on animals observed in or captured in the field; report species and age where possible. Describe how animals were caught and transported and what happened to captive animals after the study (if killed, explain why and describe method; if released, say where and when) OR state that the study did not involve wild animals.*
Reporting on sex	*Indicate if findings apply to only one sex; describe whether sex was considered in study design, methods used for assigning sex. Provide data disaggregated for sex where this information has been collected in the source data as appropriate; provide overall numbers in this Reporting Summary. Please state if this information has not been collected. Report sex-based analyses where performed, justify reasons for lack of sex-based analysis.*
Field-collected samples	*For laboratory work with field-collected samples, describe all relevant parameters such as housing, maintenance, temperature, photoperiod and end-of-experiment protocol OR state that the study did not involve samples collected from the field.*
Ethics oversight	*Identify the organization(s) that approved or provided guidance on the study protocol, OR state that no ethical approval or guidance was required and explain why not.*

Note that full information on the approval of the study protocol must also be provided in the manuscript.

Clinical data

Policy information about clinical studies

All manuscripts should comply with the ICMJE guidelines for publication of clinical research and a completed CONSORT checklist must be included with all submissions.

Clinical trial registration	*Provide the trial registration number from ClinicalTrials.gov or an equivalent agency.*
Study protocol	*Note where the full trial protocol can be accessed OR if not available, explain why.*
Data collection	*Describe the settings and locales of data collection, noting the time periods of recruitment and data collection.*
Outcomes	*Describe how you pre-defined primary and secondary outcome measures and how you assessed these measures.*

Dual use research of concern

Policy information about dual use research of concern

Hazards

Could the accidental, deliberate or reckless misuse of agents or technologies generated in the work, or the application of information presented in the manuscript, pose a threat to:

No | Yes
- [ ] [ ] Public health
- [ ] [ ] National security
- [ ] [ ] Crops and/or livestock
- [ ] [ ] Ecosystems
- [ ] [ ] Any other significant area

Experiments of concern

Does the work involve any of these experiments of concern:

No | Yes
- [ ] [ ] Demonstrate how to render a vaccine ineffective
- [ ] [ ] Confer resistance to therapeutically useful antibiotics or antiviral agents
- [ ] [ ] Enhance the virulence of a pathogen or render a nonpathogen virulent
- [ ] [ ] Increase transmissibility of a pathogen
- [ ] [ ] Alter the host range of a pathogen
- [ ] [ ] Enable evasion of diagnostic/detection modalities
- [ ] [ ] Enable the weaponization of a biological agent or toxin
- [ ] [ ] Any other potentially harmful combination of experiments and agents

ChIP-seq

Data deposition

- [ ] Confirm that both raw and final processed data have been deposited in a public database such as GEO.
- [ ] Confirm that you have deposited or provided access to graph files (e.g. BED files) for the called peaks.

Data access links<br>*May remain private before publication.*	*For "Initial submission" or "Revised version" documents, provide reviewer access links. For your "Final submission" document, provide a link to the deposited data.*
Files in database submission	*Provide a list of all files available in the database submission.*
Genome browser session<br>(e.g. UCSC)	*Provide a link to an anonymized genome browser session for "Initial submission" and "Revised version" documents only, to enable peer review. Write "no longer applicable" for "Final submission" documents.*

Methodology

Replicates	*Describe the experimental replicates, specifying number, type and replicate agreement.*
Sequencing depth	*Describe the sequencing depth for each experiment, providing the total number of reads, uniquely mapped reads, length of reads and whether they were paired- or single-end.*
Antibodies	*Describe the antibodies used for the ChIP-seq experiments; as applicable, provide supplier name, catalog number, clone name, and lot number.*
Peak calling parameters	*Specify the command line program and parameters used for read mapping and peak calling, including the ChIP, control and index files used.*
Data quality	*Describe the methods used to ensure data quality in full detail, including how many peaks are at FDR 5% and above 5-fold enrichment.*
Software	*Describe the software used to collect and analyze the ChIP-seq data. For custom code that has been deposited into a community repository, provide accession details.*

Flow Cytometry

Plots

Confirm that:

☒ The axis labels state the marker and fluorochrome used (e.g. CD4-FITC).

☒ The axis scales are clearly visible. Include numbers along axes only for bottom left plot of group (a 'group' is an analysis of identical markers).

☒ All plots are contour plots with outliers or pseudocolor plots.

☒ A numerical value for number of cells or percentage (with statistics) is provided.

Methodology

Sample preparation	To quantify protein levels in CD8+ T cells, single-cell suspensions were stained using the LIVE/DEAD Fixable Near-IR Dead Cell Stain Kit (Thermo Fisher Scientific 10119), followed by surface and intracellular staining with fluorochrome-labelled antibodies (Supplementary Data 1). Staining of cytoplasmic and nuclear antigens was performed using the Cytofix/Cytoperm Fixation/Permeabilization Kit (BD Biosciences 554714) and Transcription Factor Buffer Set (BD Biosciences 562725), respectively. Data were collected using an Aurora flow cytometer (Cytek Biosciences).<br>v10.9 (BD Biosciences).<br>For mitochondria analysis, cells were washed twice in RPMI media (no FBS) loaded with 10 nmol/mL Mitotracker Green (ThermoFisher), 25 nmol/mL TMRM (ThermoFisher), or 5umol/ml MitoSox Red for 20 min at 37°C. Probe fluorescence was then measured using an Aurora flow cytometer (Cytek Biosciences).<br>To profile the stability of the HeLa HRE-GFPODD cells, live cells were washed twice with cold PBS and immediately analysed using a LSR II flow cytometer (BD Biosciences). GFP signal was detected by a 488 nm laser with 530/30 filter. Data analysis were performed using FlowJo v10.9 (BD Biosciences).
Instrument	Aurora (Cytex Biosciences); LSR II flow cytometer (BD Biosciences).
Software	FlowJo v10.9 and FlowJo v10.10
Cell population abundance	Events were gated on live CD8+ cells prior to determining the expression of any markers. Events were gated on live HeLa HRE-GFPODD cells prior to determining the GFP signal. For mitochondrial analysis, events were gated on single-lymphocytes prior to determine fluorescent probe signal.
Gating strategy	FSC/SCC; FSC-A/FSC-H

☒ Tick this box to confirm that a figure exemplifying the gating strategy is provided in the Supplementary Information.

Magnetic resonance imaging

Experimental design

Design type	*Indicate task or resting state; event-related or block design.*
Design specifications	*Specify the number of blocks, trials or experimental units per session and/or subject, and specify the length of each trial or block (if trials are blocked) and interval between trials.*
Behavioral performance measures	*State number and/or type of variables recorded (e.g. correct button press, response time) and what statistics were used to establish that the subjects were performing the task as expected (e.g. mean, range, and/or standard deviation across subjects).*

Acquisition

Imaging type(s)	*Specify: functional, structural, diffusion, perfusion.*
Field strength	*Specify in Tesla*
Sequence & imaging parameters	*Specify the pulse sequence type (gradient echo, spin echo, etc.), imaging type (EPI, spiral, etc.), field of view, matrix size, slice thickness, orientation and TE/TR/flip angle.*
Area of acquisition	*State whether a whole brain scan was used OR define the area of acquisition, describing how the region was determined.*

Diffusion MRI ☐ Used ☐ Not used

Preprocessing

Preprocessing software	*Provide detail on software version and revision number and on specific parameters (model/functions, brain extraction, segmentation, smoothing kernel size, etc.).*

Normalization | *If data were normalized/standardized, describe the approach(es): specify linear or non-linear and define image types used for transformation OR indicate that data were not normalized and explain rationale for lack of normalization.*

Normalization template | *Describe the template used for normalization/transformation, specifying subject space or group standardized space (e.g. original Talairach, MNI305, ICBM152) OR indicate that the data were not normalized.*

Noise and artifact removal | *Describe your procedure(s) for artifact and structured noise removal, specifying motion parameters, tissue signals and physiological signals (heart rate, respiration).*

Volume censoring | *Define your software and/or method and criteria for volume censoring, and state the extent of such censoring.*

Statistical modeling & inference

Model type and settings | *Specify type (mass univariate, multivariate, RSA, predictive, etc.) and describe essential details of the model at the first and second levels (e.g. fixed, random or mixed effects; drift or auto-correlation).*

Effect(s) tested | *Define precise effect in terms of the task or stimulus conditions instead of psychological concepts and indicate whether ANOVA or factorial designs were used.*

Specify type of analysis: ☐ Whole brain ☐ ROI-based ☐ Both

Statistic type for inference (See Eklund et al. 2016) | *Specify voxel-wise or cluster-wise and report all relevant parameters for cluster-wise methods.*

Correction | *Describe the type of correction and how it is obtained for multiple comparisons (e.g. FWE, FDR, permutation or Monte Carlo).*

Models & analysis

n/a	Involved in the study
☐	☐ Functional and/or effective connectivity
☐	☐ Graph analysis
☐	☐ Multivariate modeling or predictive analysis

Functional and/or effective connectivity | *Report the measures of dependence used and the model details (e.g. Pearson correlation, partial correlation, mutual information).*

Graph analysis | *Report the dependent variable and connectivity measure, specifying weighted graph or binarized graph, subject- or group-level, and the global and/or node summaries used (e.g. clustering coefficient, efficiency, etc.).*

Multivariate modeling and predictive analysis | *Specify independent variables, features extraction and dimension reduction, model, training and evaluation metrics.*
