# Supplementary figures and images for: Lipoyl deglutarylation by ABHD11 regulates mitochondrial and T cell metabolism
Source: Nat Chem Biol. 2025 Jul 15;21(12):1915–26. doi: 10.1038/s41589-025-01965-6 (PMC12643935; doi:10.1038/s41589-025-01965-6)

**Figure 1b**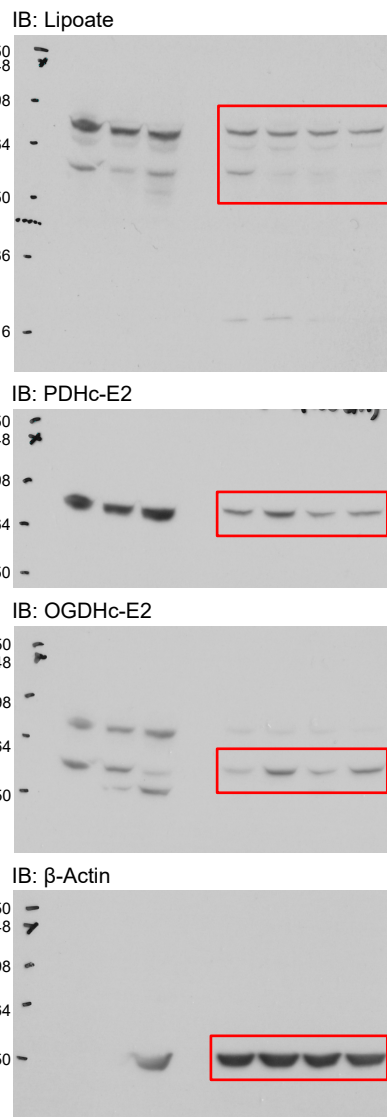**Figure 1d**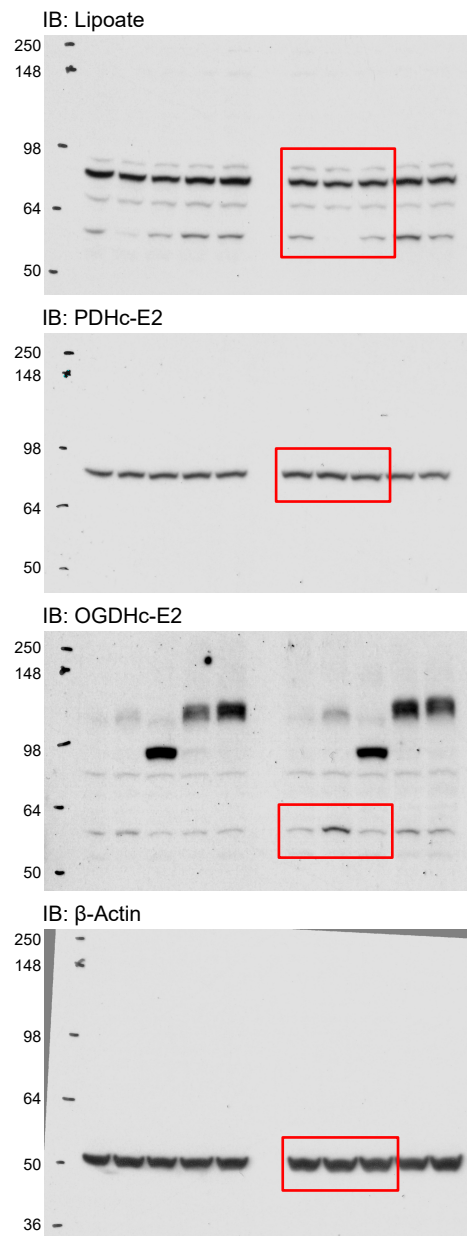**Figure 1f**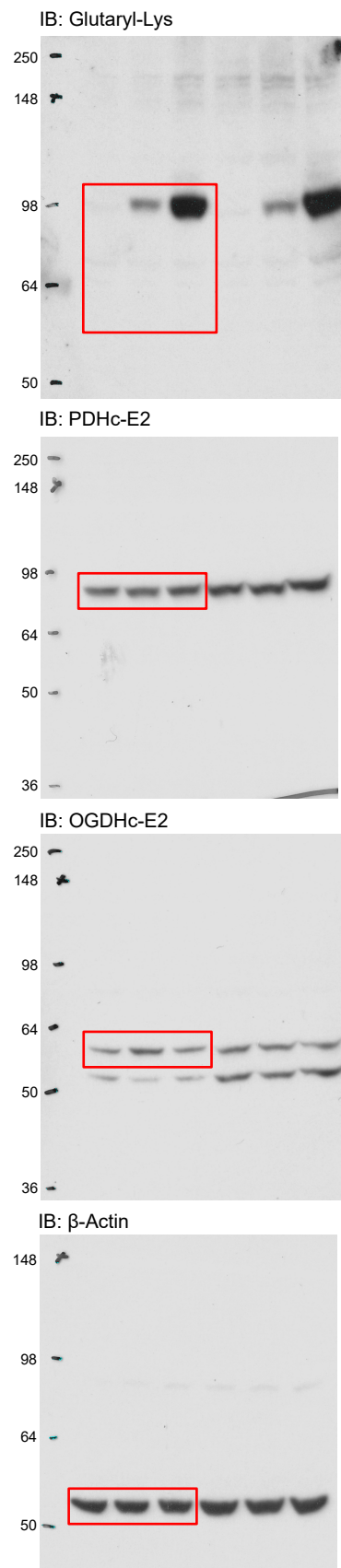

Supplement: Supplementary file 10 — Unprocessed immunoblots. [file 41589_2025_1965_MOESM10_ESM.pdf]

**Figure 4a**

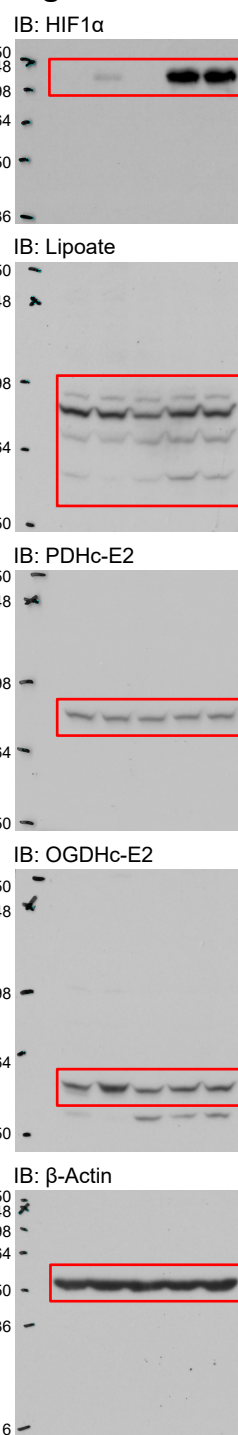

**Figure 4e**

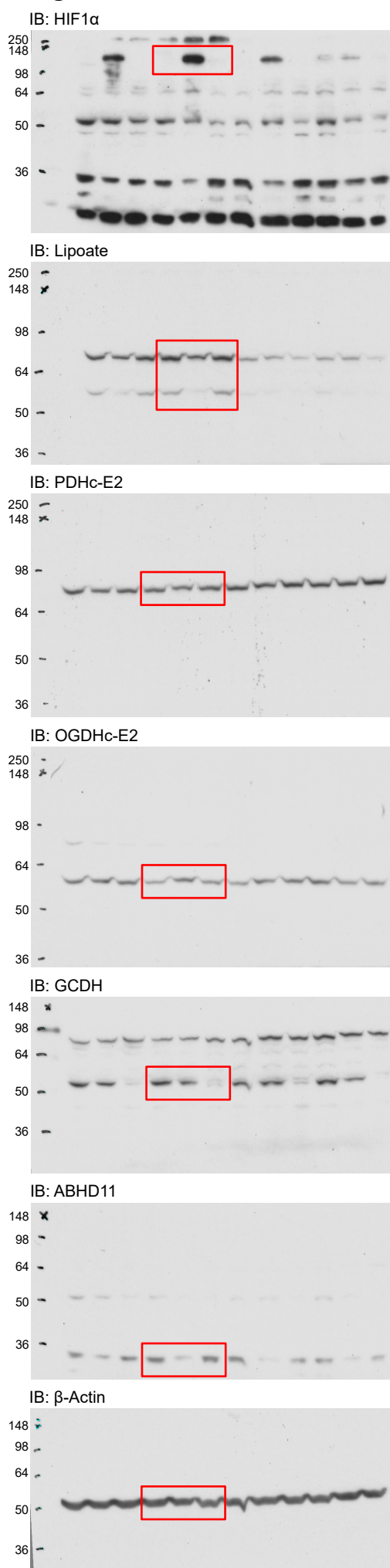

Supplement: Supplementary file 15 — Unprocessed immunoblots. [file 41589_2025_1965_MOESM15_ESM.pdf]

**Figure 5c**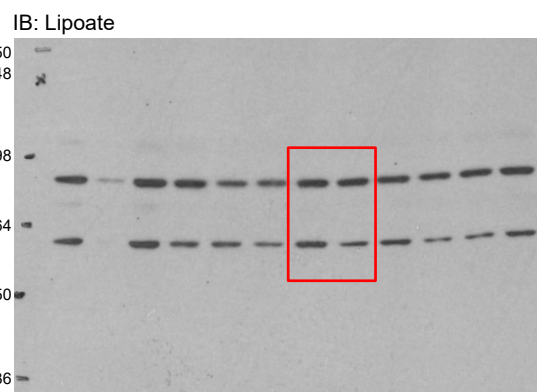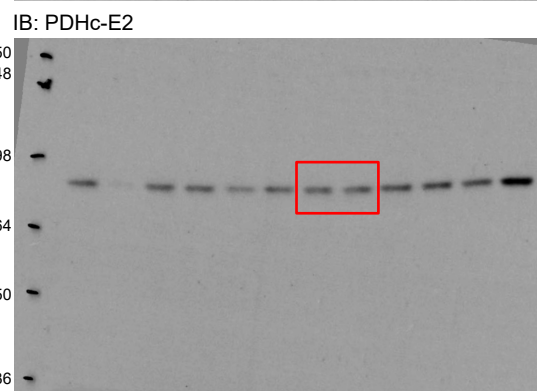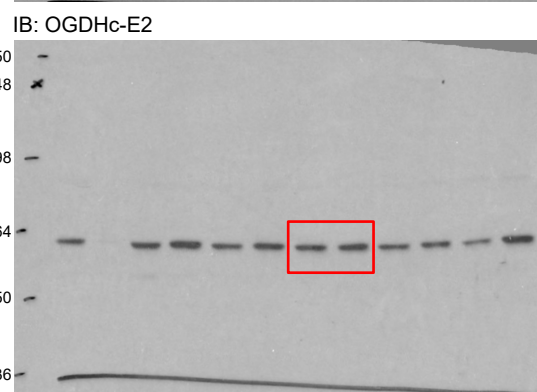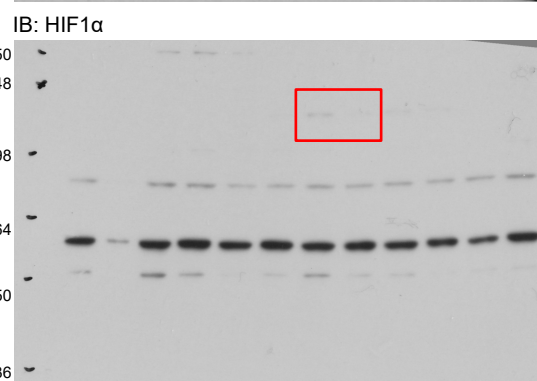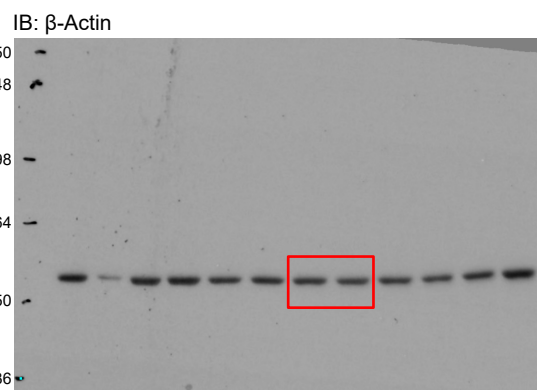**Figure 5d**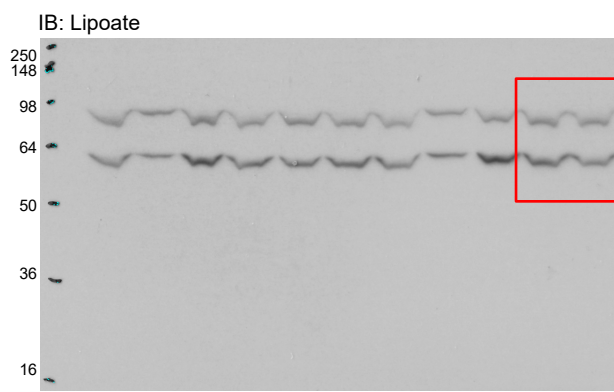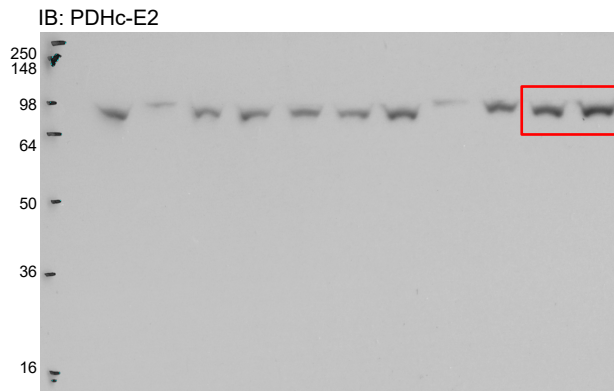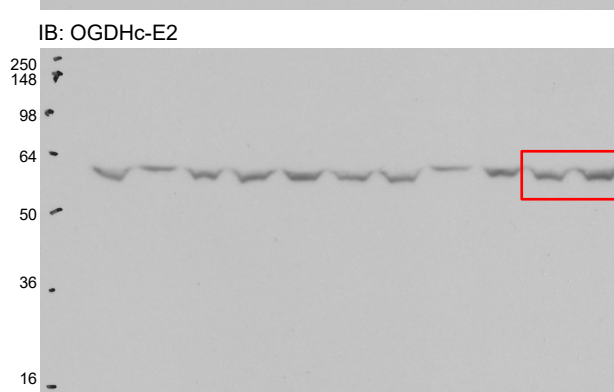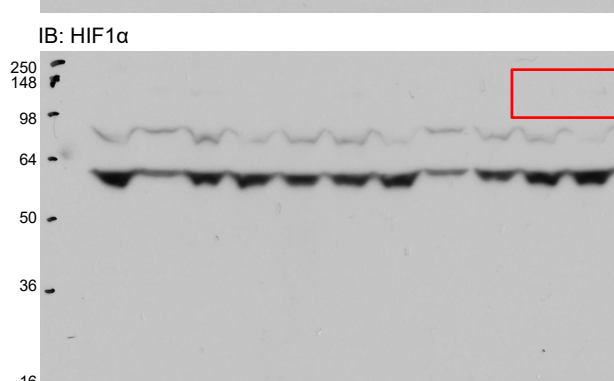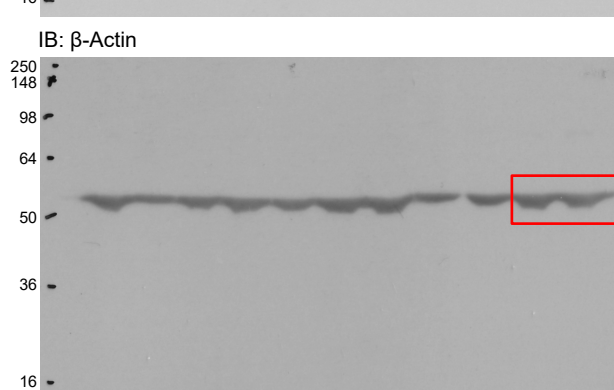

Supplement: Supplementary file 17 — Unprocessed immunoblots. [file 41589_2025_1965_MOESM17_ESM.pdf]

Extended Data Figure 1a

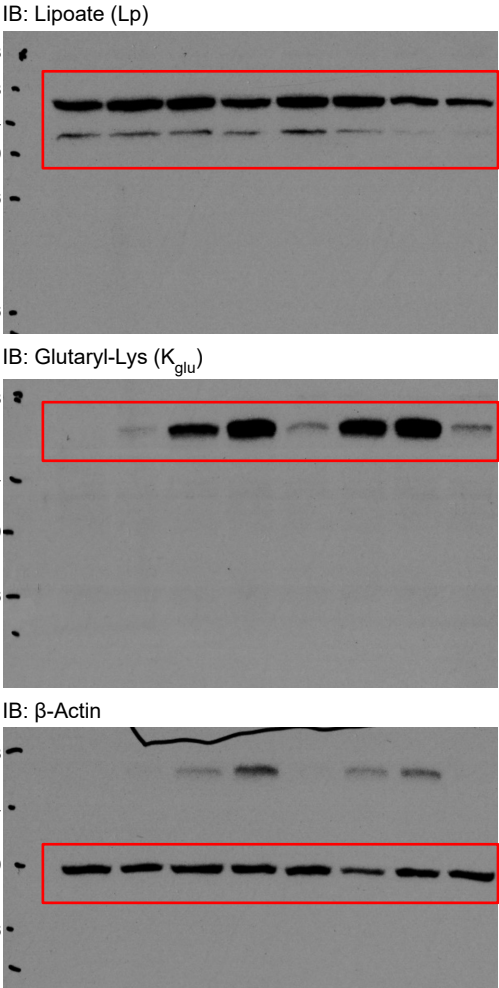

Extended Data Figure 1c

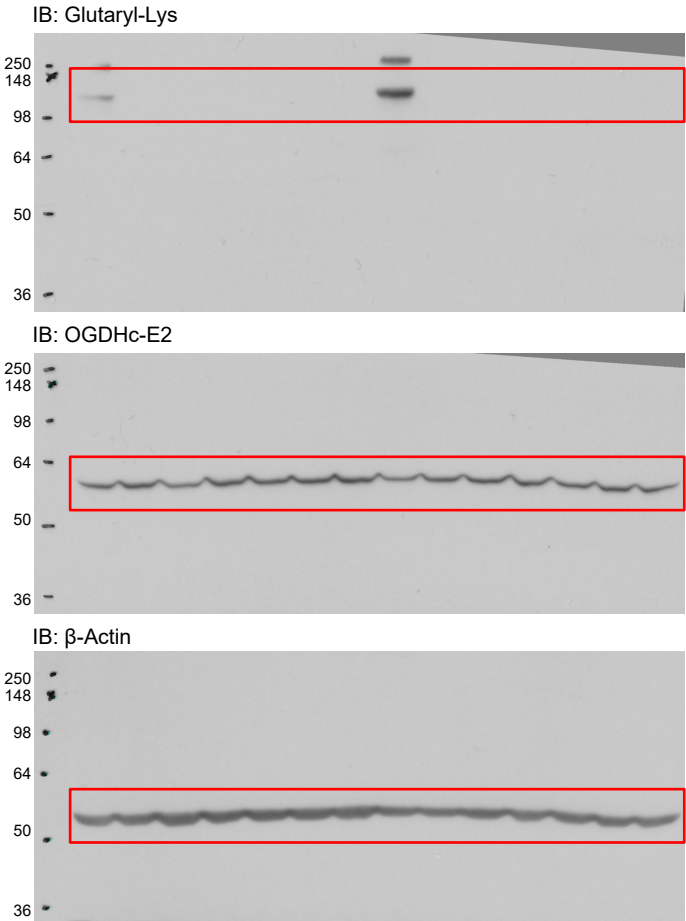

Extended Data Figure 1d

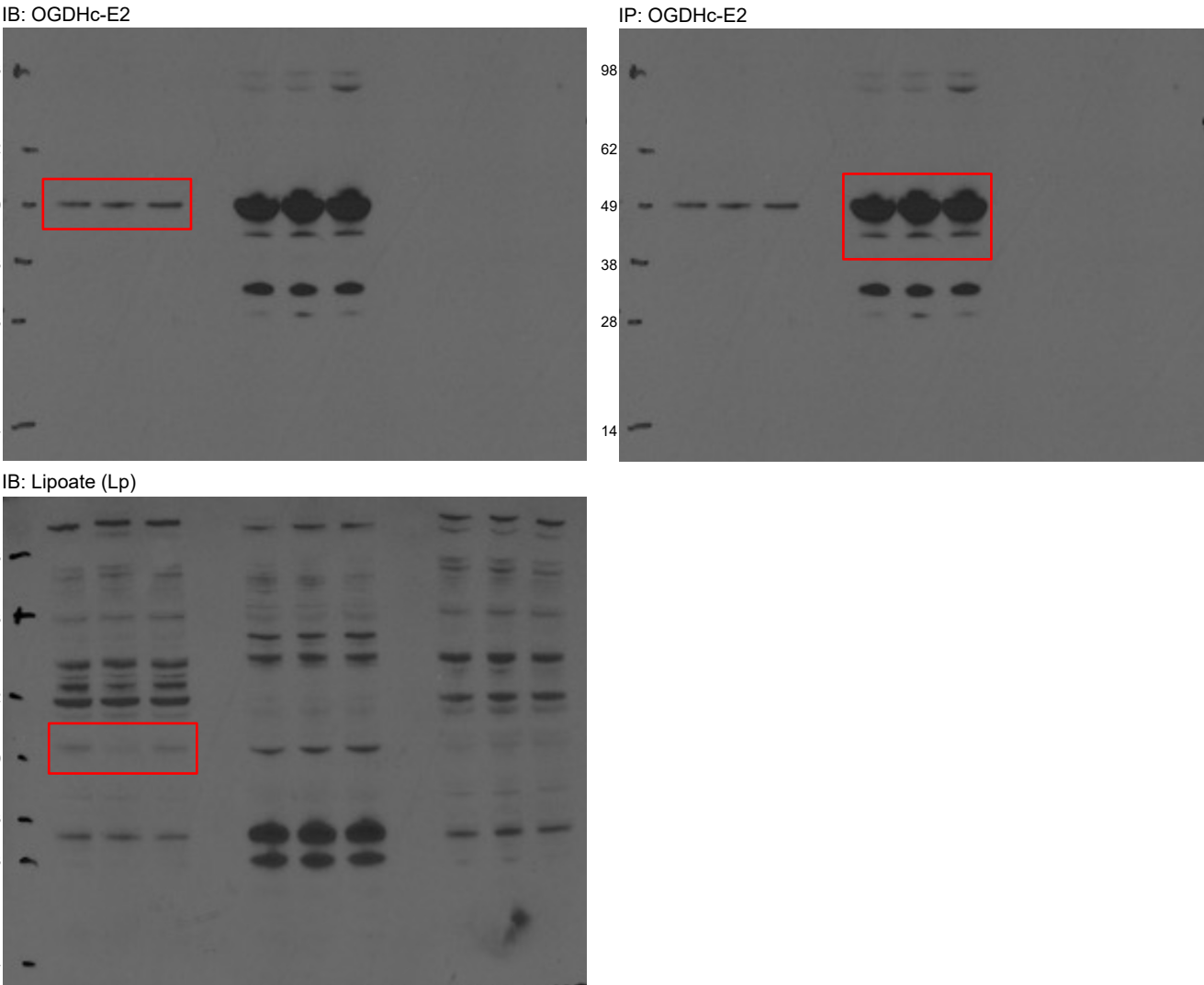

Supplement: Supplementary file 20 — Unprocessed immunoblots. [file 41589_2025_1965_MOESM20_ESM.pdf]

## Extended Data Figure 3b

IB: Lipoate (Lp)

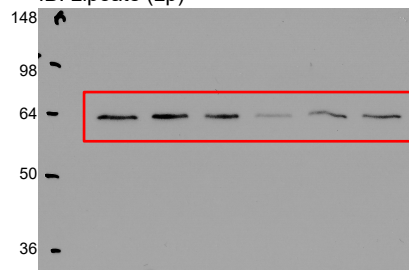

IB: OGDHc-E2

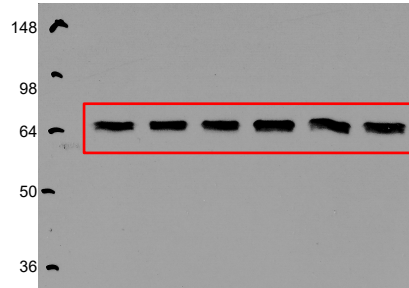

Supplement: Supplementary file 23 — Unprocessed immunoblots. [file 41589_2025_1965_MOESM23_ESM.pdf]

Extended Data Figure 5b

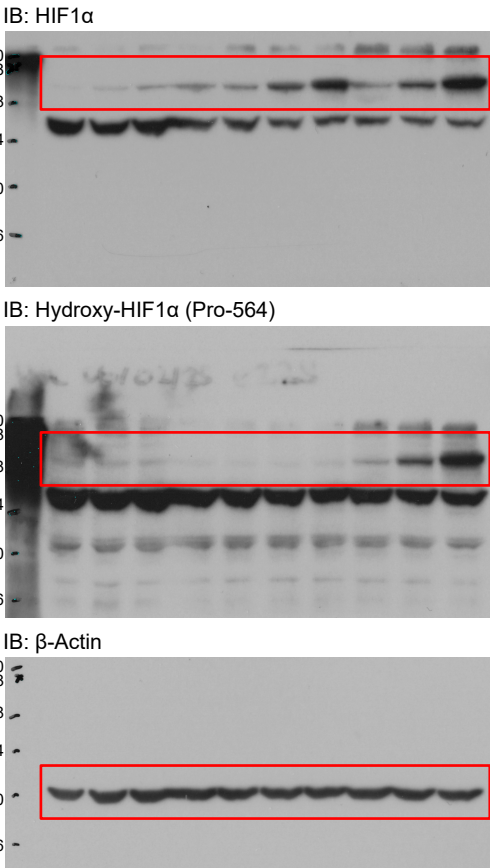

Extended Data Figure 5c

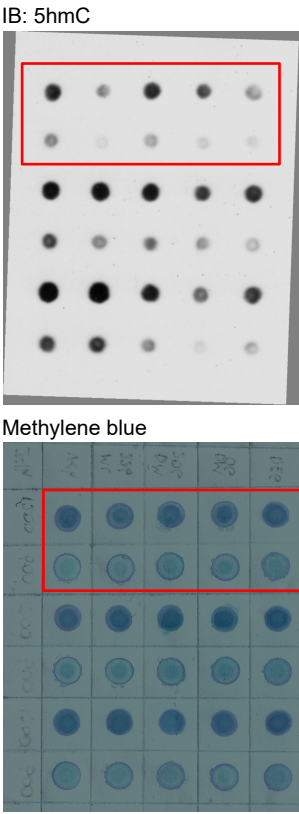

Extended Data Figure 5e

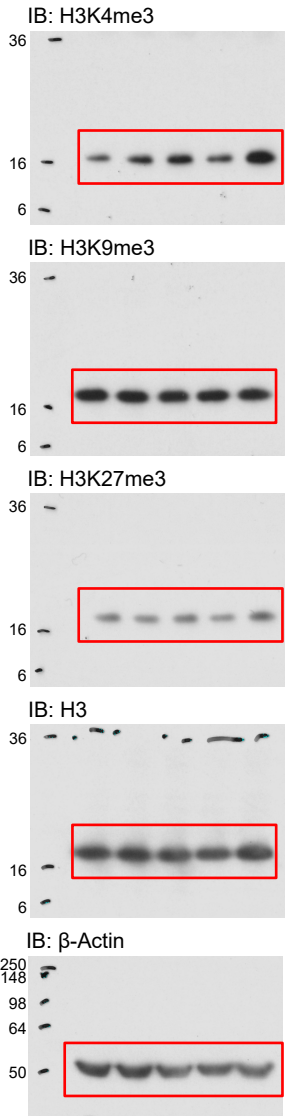

Supplement: Supplementary file 26 — Unprocessed immunoblots. [file 41589_2025_1965_MOESM26_ESM.pdf]

Extended Data Figure 7a

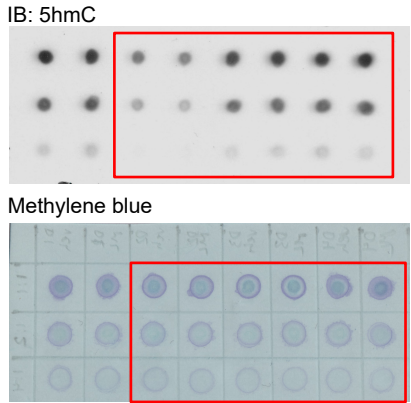

Extended Data Figure 7c

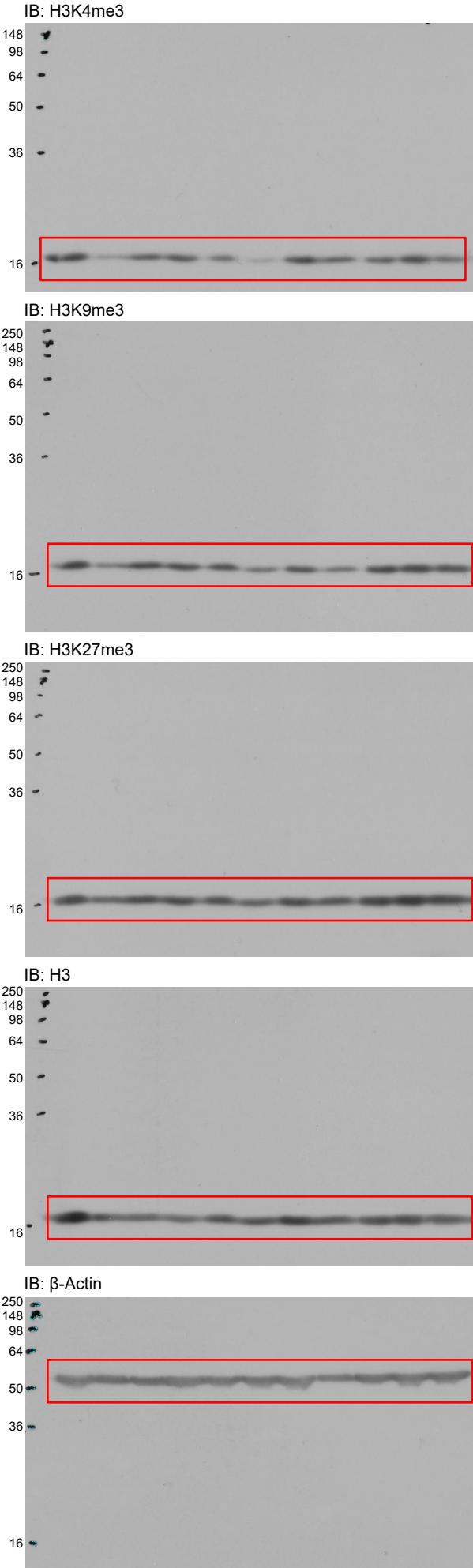

Supplement: Supplementary file 29 — Unprocessed immunoblots. [file 41589_2025_1965_MOESM29_ESM.pdf]
