# Supplementary material for: Lipoyl deglutarylation by ABHD11 regulates mitochondrial and T cell metabolism
Source: Nat Chem Biol. 2025 Jul 15;21(12):1915–26. doi: 10.1038/s41589-025-01965-6 (PMC12643935; doi:10.1038/s41589-025-01965-6)

Figure 2a

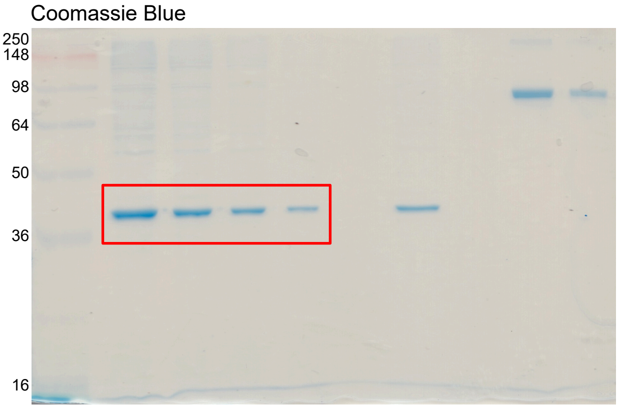

Figure 2d

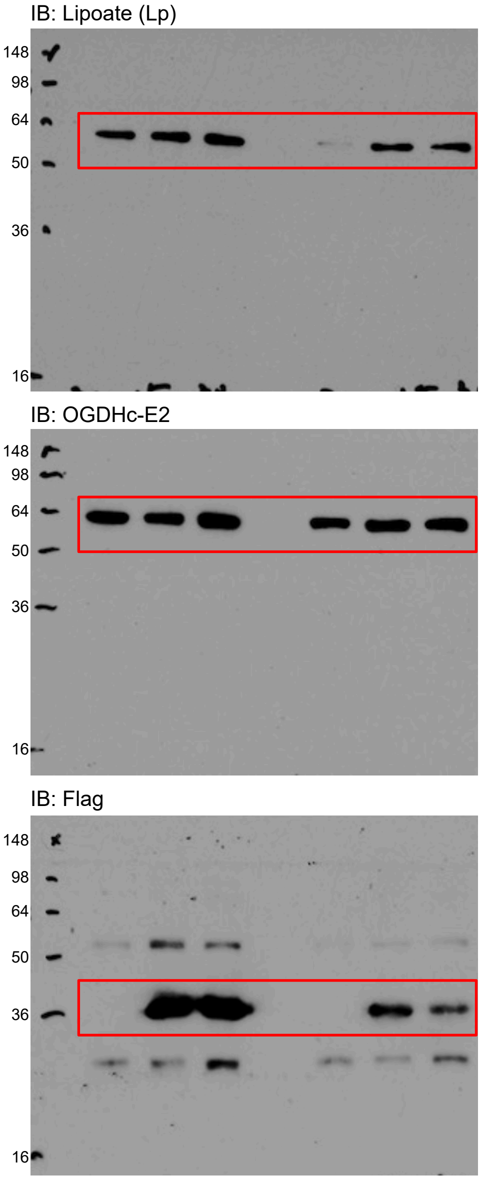

Figure 2f

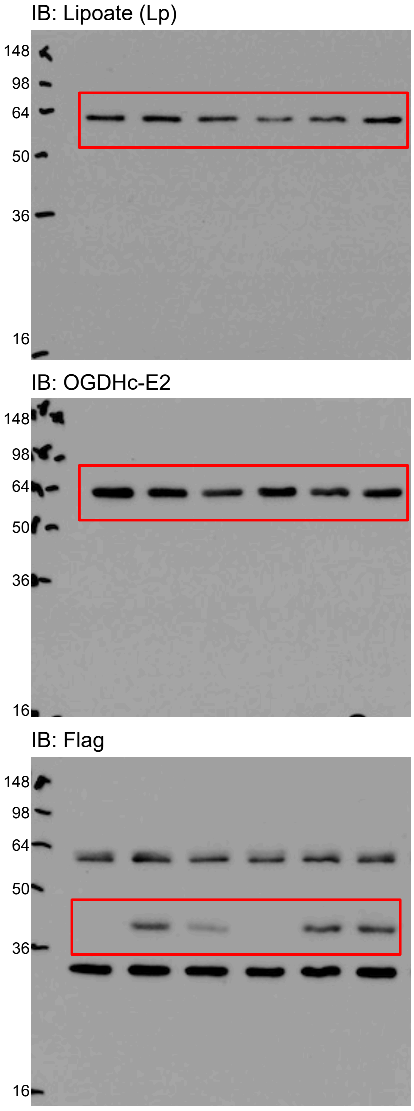

Supplement: Supplementary file 12 — Unprocessed immunoblots. [file 41589_2025_1965_MOESM12_ESM.pdf]
